# Prenatal attachment interventions: a comprehensive systematic review and meta-analysis

**DOI:** 10.1007/s00737-025-01630-w

**Published:** 2025-11-08

**Authors:** John J. Coté, Danielle B. Dilsaver, James Dimmock, Pooja Doehrman, Samantha Teague, Remington D. Coté, Brayden P. Coté, Riley Kilzer, Mahsa Omrani Sabbaghi, Jessica L. Borelli, Suena H. Massey

**Affiliations:** 1 Department of Obstetrics and Gynecology, Creighton University School of Medicine, Omaha, NE USA; 2 Department of Obstetrics and Gynecology, CommonSpirit Healthcare, Omaha, NE USA; 3 Department of Clinical Research and Public Health, Creighton University School of Medicine, Omaha, NE USA; 4 Department of Psychology, James Cook University, Townsville, QLD Australia; 5 Department of Obstetrics and Gynecology, Creighton University School of Medicine, Phoenix Campus, Phoenix, AZ USA; 6 Department of Obstetrics and Gynecology, University of Arizona School of Medicine, Phoenix, AZ USA; 7 SEED Lifespan Strategic Research Centre, School of Psychology, Faculty of Health, Deakin University, Geelong, Vic Australia; 8 Department of Biology, Creighton University, Omaha, NE USA; 9 Department of Psychology, Chestnut Hill College, Philadelphia, PA USA; 10 University of Nebraska Medical Center, Omaha, NE USA; 11 Creighton University School of Medicine, Omaha, NE USA; 12 Department of Psychological Science, University of California, Irvine, Irvine, CA USA; 13 Harvard Medical School, Boston, MA USA; 14 Brigham and Women’s Hospital, Department of Psychiatry, Boston, MA USA

**Keywords:** Maternal–fetal attachment, Paternal-fetal attachment, Prenatal attachment, Bonding, Meta-analysis, Meta-regression

## Abstract

**Purpose:**

Amidst a rapidly growing worldwide literature on non-invasive interventions to optimize parent–child relationships prenatally, the effectiveness of prenatal attachment intervention remains uncertain due to methodologic restrictions of prior systematic reviews. The current systematic review was aimed at capturing the diversity of study designs, intervention targets and methods employed reflective of this burgeoning literature. We then employed meta-regression to evaluate the impact of expected heterogeneity on estimated intervention effects.

**Methods:**

We searched MEDLINE/PubMed, Scopus, PsychINFO, Trip database, and Google Scholar for empirical prenatal attachment intervention studies published through August 11, 2025, with titles and abstracts written in English. Articles with main texts written in other languages were translated prior to analysis (PROSPERO ID CRD42021241199).

**Results:**

Prenatal attachment scores increased following intervention when examining all studies (p < . 001), randomized controlled trials only (p < .001), and studies of only male expectant parents (*p* = .017). Specific intervention methods found to be effective were touch and Leopold’s maneuver (*p* = .004), fetal movement counting (*p* < .001), music, lullaby and singing (*p* = .012), relaxation techniques (*p* = .014), cognitive therapies (*p* = .022), meditation (*p* = .003), breathing exercises (*p* = .001), and educational interventions (*p* < .001).

**Conclusions:**

While the prior systematic reviews of prenatal attachment interventions involving a total of 15 randomized controlled trials suggested equivocal effects of prenatal intervention, evidence from the current more inclusive review of non-randomized control trial (RCT) studies testing a wide range of intervention methods was substantially more compelling. More research on prenatal attachment in non-pregnant expectant partners and other caregivers and on promising but understudied interventions involving music and 3D fetal ultrasound images is recommended.

**Supplementary Information:**

The online version contains supplementary material available at 10.1007/s00737-025-01630-w.

## Article Highlights

- A variety of behavioral interventions were found to increase fetal attachment in expectant parents.
- Interventions involving music, relaxation, 3D ultrasonography are promising but understudied.
- Future comparative effectiveness trials are recommended to inform clinical guidelines and practice.

**Supplementary Information:**

The online version contains supplementary material available at 10.1007/s00737-025-01630-w.

## Introduction

The quality of interpersonal relationships in adulthood and the capacity for self-regulation in the context of these relationships are shaped by an individual’s earliest relationship(s) with primary caregiver(s) that begin to develop before birth (Bowlby et al. 1992; Salisbury et al. 2003). *Prenatal attachment*, which includes maternal- and paternal-fetal attachment,^1^ is a well-studied construct that describes expectant parents internalized mental representations of their future child, as evidenced by verbalized or reported perceptions, emotions, expectations, and behaviors related to the fetus during pregnancy (Pisoni et al. 2014). While the exact definition of prenatal attachment has been debated (Eichhorn 2012) there is a consensus that prenatal attachment predicts postpartum parenting behavior (Petri et al. 2018) and its role in children’s social and emotional development (Le Bas et al. 2021). Prenatal attachment could also influence conditions in the intrauterine environment mediated by health-related behavior (Alhusen 2008; Massey et al. 2015; Jussila et al. 2020).

There is also a consensus about the multi-faceted nature of prenatal attachment which is reflected in the variety of self-report questionnaires aimed at distinguishing between and quantifying these different facets.

For example, *cognitive attachment* involves conceptualizing the fetus as a person, attributing characteristics to the fetus, and wanting to know the fetus. *Affective attachment* involves interaction with the fetus and feeling pleasure when thinking about and interacting with the fetus. Finally, *altruistic attachment* involves maintaining proximity to the fetus, preparing for birth, and wanting to protect the fetus (Shieh et al. 2001). A number of adverse psychosocial and obstetric factors that are difficult to modify (i.e., prior miscarriages, obstetric complications, congenital defects, and intimate partner violence) can impair parent–child attachment (Cataudella et al. 2016; McNamara et al. 2019). However, other predictors of prenatal attachment are modifiable, and many scholars have attempted to intervene with these modifiable factors to induce changes in the construct (Darvishvand et al. 2018).

The variety of interventions aimed at increasing prenatal attachment has increased significantly over the past decade. Intervention methods include procedures common in routine obstetric care (i.e., counting fetal movements, ultrasound visualization of the fetus, or the assessment of fetal position through manual abdominal palpation called *Leopold’s maneuver*); common activities in a regimen of self-care (i.e., cognitive therapy, relaxation and breathing exercises, listening to music, meditation, and yoga); and childcare postpartum (i.e., singing lullabies to the fetus). However, the bulk of this research has yet to be synthesized and evaluated for translation to the clinical setting due to substantial between-study heterogeneity in study designs, outcome measures used, intervention methods tested, and the conventional restriction of systematic reviews and meta-analyses to homogeneity within each of these parameters. To illustrate, systematic reviews and meta-analyses of prenatal attachment interventions conducted since 2020 include only 15 studies and 3 intervention methods within them—counting of fetal movements, education, and ultrasound visualization of the fetus. Some reviews supported the utility of fetal movement counting (Al Amri and Smith 2022) and psychoeducation for increasing prenatal attachment (Yuen et al. 2022; Wang et al. 2023), while others found equivocal effects (Abasi et al. 2021). While interventions to improve prenatal attachment have increased, head-to-head RCT’s are scarce. Head-to-head trials may improve efficacy comparisons, improve patient care by directly comparing methods, can identify cost effectiveness, and may inform clinical practice guidelines.

The common practice of restricting meta-analyses to RCT’s provides the most rigorous estimate of a cause-effect relationships, if any, but substantially limits the generalizability of findings to relatively homogenous populations while limiting the scope of research synthesized to laboratories sufficiently funded to conduct RCT’s (Bothwell et al. 2016). We propose that the disadvantages of meta-analyses restricted to RCT’s must be considered within the context of the risk–benefit ratio of tested interventions. The risk of overestimating the effect of intervention is more dire for invasive interventions such as medication or surgery relative to non-invasive interventions such as those involving the very activities typically encountered in a regimen of self-care (i.e., relaxation, meditation, or breathing exercises), during pregnancy (i.e., singing lullabies to the fetus), or routine obstetric care (Leopold’s maneuver or fetal ultrasound imaging). In these cases, studies less costly than RCT’s such as controlled trials without randomization or *non-randomized controlled trials (NCT’s*), before-after comparisons of outcomes without a control group or *quasi-experimental studies*; and *cross-sectional comparisons* of an outcome after intervention versus treatment as usual, provide valuable information that can inform the direction of future research.

The overarching goal of the current systematic review and series of meta-analyses is to extend prior knowledge through the inclusion of a substantially expanded range of prenatal attachment intervention studies employing a variety of study designs and intervention methods, involving both pregnant and non-pregnant expectant parents. We evaluated the extent to which differences in estimated effects were attributable to these various types of expected heterogeneity using meta-regression. *Meta-regression*, which employs regression analysis to synthesize effect estimates from multiple studies by identifying sources of heterogeneity between studies, and adjusting for between-study differences, is particularly well-matched to the current task because it enables the inclusion of a much broader range of study designs, outcome measures, and intervention methods (Morton et al. 2010). Specifically, we conducted a series of meta-analyses inclusive of:

- All RCT’s, NCT’s, quasi-experimental, and cross-sectional studies;
- Only RCT’s
- Different methods of intervention
- Different measures of attachment
- Non-pregnant expectant parents.

We then employed meta-regression analyses to identify sources of heterogeneity between and within studies and to estimate intervention effects independent of these variables (Table 1).

**Table 1:** Meta-analyses (TOP) and meta-regression (BOTTOM) conducted in *N* = 107 studies reviewed

Meta-analyses	n	Design	Intervention method(s)	Outcomes assessed	Which expectant parent
	77	Any	Any	After intervention only	Pregnant or both
By study design	66	Any	Any	Before and after intervention	Pregnant or both
	40	RCT	Any	Before and after intervention	Pregnant or both
	13	Any	Leopold’s maneuver	Before and after intervention	Pregnant or both
	20	Any	Fetal movement counting	Before and after intervention	Pregnant or both
	7	Any	Ultrasound	Before and after intervention	Pregnant or both
	31	Any	Educational	Before and after intervention	Pregnant or both
By intervention methods(s)	17	Any	Music/lullaby/singing	Before and after intervention	Pregnant or both
	21	Any	Relaxation techniques	Before and after intervention	Pregnant or both
	6	Any	Cognitive therapies	Before and after intervention	Pregnant or both
	5	Any	Yoga	Before and after intervention	Pregnant or both
	8	Any	Meditation practices	Before and after intervention	Pregnant or both
	10	Any	Breathing exercises	Before and after intervention	Pregnant or both
By target	7	Any	Any	Before and after intervention	Non-pregnant only
Meta-regressions				Covariates included	
By intervention method(s) tested					
Single vs. multi-method intervention(s)			Participant age, number of interventions, follow-up, study design, gestational age, marital status, planned pregnancy, knowledge of fetal sex, income, employment status, level of education, history of abortion, religious affiliation, mode of conception, marital satisfaction, and primigravity		
By intervention duration					

## Materials and methods

### Search strategy

**Fig. 1:**
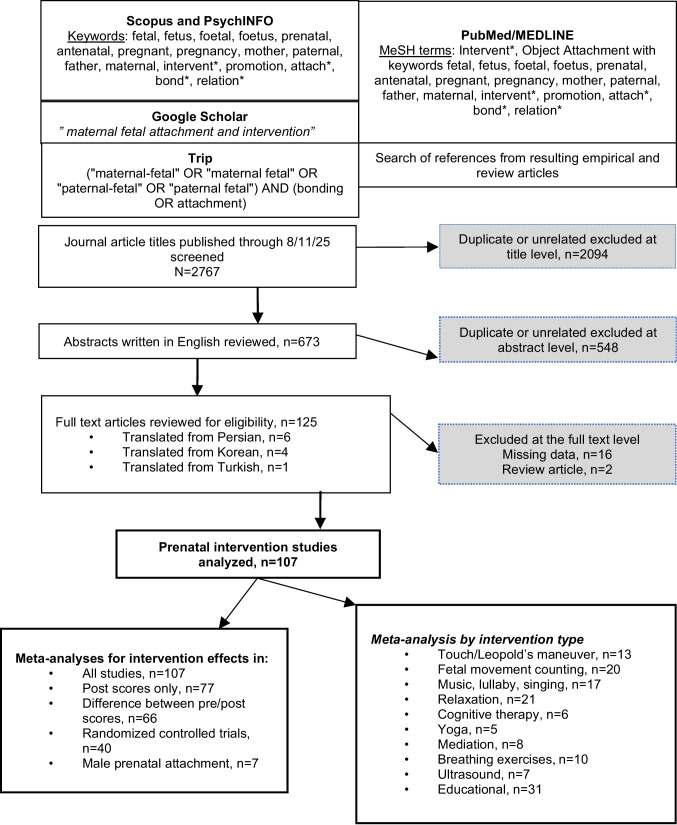
PRISMA search strategy, data extraction, and meta-analyses

The flow chart in Fig. 1 illustrates the systematic selection of studies in accordance with the aforementioned theoretical framework, and in accordance with the Preferred Reporting Items for Systematic Reviews and Meta-Analysis (PRISMA) reporting guidelines (PROSPERO registration: CRD42021241199) (Page et al. 2021). On August 11, 2025, electronic databases were searched using the specific keywords shown. Studies with missing data, review articles, conference abstracts, commentaries, letters to the editor, and other publications that were not peer-reviewed were excluded. References from published systematic reviews (Abasi et al. 2021; Skelton et al. 2022; Yuen et al. 2022; Wang et al. 2023) were additionally searched.

Following the removal of duplicates and unrelated articles, the remaining 673 abstracts were reviewed independently by two authors, JC and RC, with discrepancies resolved by JC, for studies that compared participants’ attachment scores before and after a described intervention with or without inclusion of a control group. Non-English language articles determined to be eligible based on its English abstract were translated to allow for review of the full text. Articles written in Persian were translated directly by one of the authors who is Persian literate (MO). Articles with main texts written in languages other than Persian or English were translated digitally using Google Translate (Translate 2023) or ChatGPT (Open 2023).

### Data extraction

A total of 125 full-text articles were reviewed for presence of the following data: (a) study design; (b) description of the intervention studied; (c) tool(s) used to quantify prenatal attachment; (d) the target of intervention (i.e., pregnant versus other expectant parents or both); (e) estimated effects of interventions on attachment score, using statistical tests appropriate to the study design; (f) demographic characteristics of study participants.

### Meta-analyses (Table 1, top and Supplemental Fig. 1)

A series of meta-analyses estimated the pooled difference in prenatal attachment scores between control and intervention groups. Specifically, as shown from top to bottom in Table 1, we first estimated effects of all study designs at post-intervention, regardless of pre-intervention assessments comparing control groups (no intervention) to intervention groups. Second, we estimated effects of all study designs comparing the difference between pre- and post-intervention. Third, we estimated effects of the most rigorous study design—RCTs—comparing the difference between pre- and post-intervention. We estimated effects of all study designs of 10 categories of intervention methods (touch/Leopold’s maneuver, fetal movement; music/lullaby/singing, relaxation, cognitive processing therapy or cognitive behavioral therapy, yoga, meditation, breathing, ultrasound, and education). Finally, we synthesized studies that enrolled exclusively non-expectant partners, by study design.

### Meta-regressions (Table 1, bottom and Supplemental Fig. 1)

To understand the impact of expected between-study heterogeneity, we conducted a series of meta- regressions aimed at evaluating the extent to which various study design and intervention methods tested contributed to heterogeneity in the estimated effects of intervention. Meta-regressions conducted to evaluate the effect of study-specific factors and patient-specific factors on heterogeneity are shown at the bottom of Table 1. *Study specific characteristics* examined were: (a) study design factors (randomization, follow-up time); (b) type of interventions; (c) single versus multiple interventions tested concomitantly; and (d) the duration of interventions. *Patient-specific characteristics* examined were participant and gestational ages at the time of intervention, marital status, planned pregnancy, knowledge of fetal sex, income, employment status, level of education, history of abortion, religious affiliation, natural conception, marital satisfaction, and primigravity.

### Assessment of risk of bias

#### Quality appraisal

Authors BC and JC assessed methodological quality of full text articles independently using the revised Cochrane risk of bias tool, or the risk of bias in non-randomized studies of interventions (Sterne et al. 2016; Sterne et al. 2019; Moola et al. 2020; Higgins et al. 2024). Any disagreement was discussed amongst the two authors until an agreement was made on the final quality assessment.

To account for between-study differences in prenatal attachment measures used, all meta-analyses estimated pooled Hedges’ g effect sizes that were based on study-specific sample sizes, standard deviations, and mean differences in prenatal attachment (Hedges and Olkin 2014). Between-study heterogeneity was quantified as low, moderate, and high heterogeneity using I^2^ with thresholds at 25%, 50%, and 75%, respectively (Higgins and Thompson 2002). Analyses with low heterogeneity (I^2^ < 25%) were estimated via fixed-effects meta-analysis with an inverse-variance estimator. Analyses with moderate and high heterogeneity (I^2^ ≥ 25%) were estimated via random-effects meta-analysis with a restricted-maximum likelihood estimator. Funnel plots were evaluated to assess small study/publication bias. Stata v. 18.5 (StataCorp LLC, College Station, TX) was used for all analyses. Statistical significance was indicated by two-tailed *p* < 0.05. All data can be found at 10.5281/zenodo.17021553.

## Results

### Description of included studies

As shown in Fig. 1, from the 125 full-text articles that were assessed for eligibility, 107 articles met inclusion criteria. There were 208 observations total (this included the effect of the same intervention at different timepoints (i.e., measuring the effect immediately after the intervention and 2 weeks later) as well as having more than one intervention in a study (i.e., doppler alone and doppler with mindfulness). Out of the 107 articles, there were 18 articles (with 21 observations) that only examined attachment post intervention, 9 articles with multiple independently measured interventions (i.e., music and lullaby, or 3D ultrasound and 3D printed model), 15 articles with multiple interventions measured as a combined intervention (i.e., Loving-Kindness and Compassion Meditation Program or nested Visual, Auditory, Read and Kinesthetic Strategies). Included studies were published across a total of 24 countries (Australia; *n* = 4, China; *n* = 2, Egypt; *n* = 3; Ethiopia; *n* = 1, Finland; *n* = 1, India; *n* = 7, Indonesia; *n* = 2, Iran; *n* = 29, Ireland; *n* = 1, Italy; *n* = 3, Japan; *n* = 1, Korea; *n* = 9, Netherlands; *n* = 2, Nigeria; *n* = 1, Norway; *n* = 1, Pakistan; *n* = 1, Portugal; *n* = 1, Romania; *n* = 1, Sweden; *n* = 1, Taiwan; *n* = 2, Thailand; *n* = 1, Turkey; *n* = 16, United Kingdom; *n* = 5, United States; *n* = 14) and inclusive of 4 languages (English; *n* = 98, Korean; *n* = 4, Persian; *n* = 6, Turkish; *n* = 1). (Tables 2, 3, 4, and 5).

**Table 2:** Summary of characteristics of included studies using the maternal fetal attachment scale (MFAS)

Name	Study Design	Intervention	Age Range	Gravidity	Control N	Intervention N	Control Mean	Intervention Mean
Abasi et al. 2010 (Iran)	Quasi-experimental	Fetal Kick Counts		Primigravida	42	41	3.42	3.52**
Abasi et al. 2010 (Iran)	Quasi-experimental	Fetal Kick Counts		Primigravida	42	41	3.42	3.96**
Abasi et al. 2013 (Iran)	RCT	MFA Education	18–35	Primigravida	43	40	3.42	3.96
Abasi et al. 2023 (Iran)	RCT	MFA Education	18–40	Both	51	49	3.21	3.75
Akbarzade et al. 2014 (Iran)	RCT	MFA Education	18–35	Primigravida		75		61.9*
Alhusen et al. 2021 (USA)	RCT	Cognitive Behavioral Therapy	24.5	Both	30	30	78.20	84.10
Amiri and Firouzabadi 2023 (Iran)	Quasi-experimental	Cognitive Behavioral Therapy	18–35	Primigravida	20	20	79.2	100.15
Arasteh et al. 2020 (Iran)	RCT	Cognitive Behavioral Therapy	28.66	Both		25		84.36*
Asari and Tiwari 2021 (India)	Longitudinal	Fetal Kick Counts	18–28	Primigravida		40		101.25*
Azogh et al. 2018 (Iran)	Quasi-experimental	Cognitive Behavioral Therapy	> 18	Multigravida	50	55	80.90	92.36**
Ezzat Abdel Gawad Badar et al. 2023 (Egypt)	Quasi-experimental	Fetal Kick Counts	20–35	Primigravida		60		60.32*
Baghdari et al. 2016 (Iran)	Quasi-experimental	MFA Education	18–35	Multigravida	27	28	60.81	75.75
Chang et al. 2004 (Korea)	Pretest/Posttest	Taegyo	24–40	Both		49		78.7*
Chang et al. 2015 (Taiwan)	RCT	Music	> 18	Both	151	145	95.60	100.96
Ekrami et al. 2020 (Iran)	RCT	MFA Education	15–49	Multigravida	39	37	76.50	96.60
Estevao et al. 2025 (Italy)	single-arm feasibility study	Yoga	33,	Multigravida		15		90.17*
Estevao et al. 2025 (Italy)	single-arm feasibility study	Yoga	33,	Multigravida		15		93.27*
Gheibi et al. 2020 (Iran)	RCT	Mindfulness	18–35	Both	20	18	83.60	95.39
Salemi Ghomshe et al. 2023 (Iran)	Quasi-experimental	Psychoeducation	30.4	Both	40	40	65.95	93.05**
Hasanzadeh et al. 2025 (Iran)	RCT	MFA Education	29.17	Primigravida	42	42	57.14	66.43
El-Sayed et al. 2021 (Egypt)	Quasi-experimental	MFA Education	18–35	Both		100	79.6	99.2*
Hoseini et al. 2020 (Iran)	RCT	MFA Education, Cognitive Behavioral Therapy	25–35	Both		47		99**
Jangjoo et al. 2021 (Iran)	RCT	MFA Education	18–35	Both	34	37	64.79	104.43
Jussila et al. 2020 (Finland)	RCT	Ultrasound	17–40	Both	28	41	4.20	4.10
Khalili et al. 2020 (Iran)	RCT	MFA Education	> 20	Both	50	50	65.50	80.36
Kim and Chun 2020 (Korea)	Quasi-experimental	MFA Education, Psychoeducation	33.79	Both	30	29	65.4	65.69
Koh et al. 2021 (Korea)	Pretest/Posttest	MFA Education, Psychoeducation	32.3	Primigravida		33		77.67*
Kordi et al. 2016 (Iran)	RCT	Relaxation Training	24.1	Primigravida	32	35	90.22	94.26
Lavi et al. 2015 (USA)	Pretest/Posttest	Psychoeducation	18–40	Both		64		85.25*
Lee et al. 2002 (Korea)	Nonequivalent experimental group quasi-experimental	Ultrasound			123	126	72.46	73.46**
Lee et al. 2023 (Korea)	RCT	Virtual Reality	35	Both	40	40	97.7	98.2
Mahmoudi et al. 2023 (Iran)	RCT	MFA Education	20–40	Both	32	29	84.43	90.2
Maleki et al. 2025 (Iran)	RCT	Mindfulness						
Marzouk and Nabil 2015 (Egypt)	Quasi-experimental	MFA Education, Touch/Leopold Maneuver	27.9	Primigravida	40	40	63.90	69.60
Mesgarzadeh et al. 2020 (Iran)	Semi-experimental	Fetal Kick Counts		Primigravida	55	55	3.84	4.06**
Mikhail et al. 1991 (USA)	RCT	Fetal Kick Counts	17–37	Both	88	125	2.97	3.80
Mojahed et al. 2019 (Iran)	RCT	Cognitive Behavioral Therapy	20–40	Multigravida	30	30	90.90	99.80
Mokaberian and Dehghanpouri 2021 (Iran)	RCT	Relaxation Training	18–35	Primigravida	30	30	86.30	97.40**
Mokaberian et al. 2021 (Iran)	RCT	MFA Education, Psychoeducation, Prenatal Education	< = 35	Primigravida	30	30	85.46	98.16
Monickaraj et al. 2020 (India)	RCT	Fetal Kick Counts		Both	31	31	76.94	87.23
Muzik et al. 2012 (USA)	Pretest/Posttest	Yoga, Mindfulness	32.41	Primigravida		18		95.5*
Nasab et al. 2023 (Iran)	RCT	Cognitive Behavioral Therapy	15–42	Both	30	30	80.16	94.06
Nwogu 2011 (Nigeria)	Prospective	Ultrasound	< 19- > 40	Multigravida		289		3.74*
Öhman and Waldenström 2010 (Sweden)	RCT	Art Therapy	15–44	Both	891	912	3.44	3.50
Park and Lee 2018 (Korea)	Quasi-experimental	Prenatal Education	25–41	Primigravida	29	28	63.93	81.75**
Parsa et al. 2016 (Iran)	RCT	MFA Education, Psychoeducation	18–37	Primigravida	55	55	98.20	102.82**
Salehi et al. 2017 (Iran)	RCT	Fetal Kick Counts	25.33	Primigravida	29	23	88.64	96.30
Sansone et al. 2024 (Australia)	prospective longitudinal observational study	Mindfulness	32	Multigravida		13		101.23*
Shokri Shams et al. 2021 (Iran)	Quasi-experimental	Music	20–35	Both	15	15	83.20	96.33
Shin and Kim 2011 (Korea)	Nonequivalent control group unsynchronized design	Music	< 30- >= 35	Both	116	117	65.73	64.81
Shreffler et al. 2019 (USA)	RCT	doppler	15–40	Both	9	8	88.22	96.57
Shreffler et al. 2019 (USA)	RCT	Mindfulness	15–40	Both	9	11	88.22	97.00
Shreffler et al. 2019 (USA)	RCT	doppler and Mindfulness	15–40	Both	9	6	88.22	97.83
Toosi et al. 2014 (Iran)	Interventional Clinical Trial	Relaxation Training	18–35	Primigravida	42	42	61.10	63.60
Toosi et al. 2017 (Iran)	Semi-experimental Clinical Trial	Relaxation Training	18–40	Primigravida	40	40	62.00	67.00
Yang and Kim 2010 (Korea)	Nonequivalent control group pretest/posttest	Taegyo	24–40	Primigravida	27	25	70.80	79.00**
Yuan et al. 2018 (China)	RCT	MFA Education, Psychoeducation	> 20	Primigravida	14	15	2.18	4.51

^*^ Pretest versus posttest (no control); **non-English language

**Table 3:** Summary of characteristics of included studies using the prenatal attachment inventory (PAI)

Name	Study Design	Intervention	Age Range/Mean	Gravidity	Control N	Intervention N	Control Mean	Intervention Mean
Akarsu and Rathfisch 2018 (Turkey)	RCT	Yoga	20–35	Primigravida	32	31	61.90	67.60
Ibıcı Akca et al. 2023 (Turkey)	RCT	Mindfulness	18–44	Both	46	48	43.83	54.65
Astuti et al. 2021 (Indonesia)	Cross-sectional	Yoga	20–35	Primigravida	65	65	64.38	69.23
Astuti et al. 2021 (Indonesia)	Cross-sectional	Ultrasound	20–35	Primigravida	5	125	59.2	67.11
Ayala et al. 2025 (USA)	Cross-sectional	Fetal Kick Counts	18–43	Both		51	56.6	10.3
Badem and Mucuk 2022 (Turkey)	RCT	Fetal Kick Counts	19–35	Primigravida	43	42	65.51	73.8
Baltaci and Başer 2022 (Turkey)	RCT	Lullaby	28.63	Both	30	30	54.36	66.7
Bellieni et al. 2007 (Italy)	RCT	MFA education	31.5	Both	41	36	59.90	65.50
Bilgin et al. 2020 (Turkey)	Prospective	Prenatal Education	27.5	Primigravida	100	115	70.1	71.7
Celik and Ergin 2020 (Turkey)	RCT	Touch/Leopold's	20–38	Both	50	50	67.10	76.10
Chetu 2015 (Romania)	quasi-experimental	Art Therapy	27–39	Primigravida	8	8	46.50	37.38
Delaram et al. 2018 (Iran)	RCT	Fetal Kick Counts	26.35	Primigravida	104	104	92.78	93.75
Estevao et al. 2025 (Italy)	single-arm feasibility study	Yoga	33	Multigravida		15		63.36
Estevao et al. 2025 (Italy)	single-arm feasibility study	Yoga	33	Multigravida		15		64.64
Fiskin and Sahin 2018 (Turkey)	RCT	Relaxation Training	19–35	Both	30	30	64.5	69.6
Kartal and Karaman 2018 (Turkey)	Semi-experimental	MFA education, Prenatal education	26.29	Both		44		71.88 * **
Kılıç and Dereli Yılmaz 2023 (Turkey)	RCT	Virtual Reality	23.52	Primigravida	63	63	67.33	71.09
Murphy-Tighe et al. 2025 (Ireland)	convergent parallel mixed-methods design	Music (Mums Using Music program)	32.75	Primigravida	5	4	55.5	61.33
Ozbek and Pinar 2023 (Turkey)	RCT	Touch/Leopold's	21–40	Primigravida	36	36	68.05	74.16
Persico et al. 2017 (Italy)	Concurrent cohort, quasi-experimental	Music	32.6	Both	85	83	65.40	65.60
Rincy and Nalini 2014 (India)	RCT	Fetal Kick Counts	18–27	Primigravida		50		64.38*
Saastad et al. 2011 (Norway)	RCT	Fetal Kick Counts	17–43	Both	473	478	59.54	59.34
Ağapınar Şahin and Bekar 2023 (Turkey)	RCT	Yoga	> 18	Both	42	43	63	70.69
Skelton et al. 2023 (UK)	Cross-sectional	Ultrasound	> 18	Both		235		46.77*
Skelton et al. 2024 (UK)	prospective observational cohort	Ultrasound	32.22	Primigravida		47		Combined with MRI 42.73*
Skelton et al. 2024 (UK)	prospective observational cohort	MRI	32.22	Primigravida		9		Combined with Ultrasound 42.73*
Sugishita and Kitagawa 2019 (Japan)	quasi-experimental	Music	n/a	Both	47	38	50.6	55.2
Wahyuni et al. 2024 (Indonesia)	quasi-experimental	spiritual-based stimulations		Primigravida	33	33	44.18	75.58
Williams 2015 (USA)	Longitudinal mixed methods	Yoga, Mindfulness	18–35	Primigravida		15		62.8*

^*^Pretest versus posttest (no control); **non-English language

**Table 4:** Summary of characteristics of included studies using the maternal antenatal attachment scale (MAAS)

Name	Study Design	Intervention	Gravidity	Intervention Age Range or mean	Control N	Intervention N	Control Mean	Intervention Mean
Arioli et al. 2025 (Italy)	RCT	Active Music and Passive Music	Both	35.69		250		Combined Active and Passive Music 76.8*
Baltaci et al. 2023 (Turkey)	RCT	Music	Both	27.67	40	40	71.45	74.92
Baltaci et al. 2023 (Turkey)	RCT	Lullaby	Both	29.92	40	40	71.45	78.07
Sabancı Baransel and Uçar 2023 (Turkey)	RCT	MFA Education	Both	> 20	77	77	74.14	78.46
Bhandari et al. 2025 (India)	RCT	Fetal Kick Counts	Primigravida	24.4	40	40	75.25	81.15
Briscoe et al. 2022 (UK)	Longitudinal Mixed Methods	Psychoeducation	Multigravida	21–29	36	36	68.25	77.02*
Carvalho et al. 2025 (Portugal)	quasi-experimental	Music	Both	35.87	9	10	62.85	63.12
Carvalho et al. 2025 (Portugal)	quasi-experimental	Vocal Training	Both	34.9	9	8	62.85	62.12
Coté et al. 2020 (USA)	RCT	Ultrasound	Both	19–45	48	45	76.81	81.75*
Coté et al. 2020 (USA)	RCT	Ultrasound 3D Printing	Both	19–45	48	48	76.27	84.96*
Coté et al. 2023 (USA)	RCT	3D Printing	Both	19–45	46	44	78.33	83.89*
Coté et al. 2023 (USA)	RCT	3D Picture	Both	19–45	49	49	79.71	83.41*
Côté-Arsenaultet al. 2014 (USA)	Mixed Methods	Fetal Kick Counts Relaxation Training Prenatal Education	Multigravida	22–41	11	12	4.04	3.99
Cox et al. 2021 (UK)	RCT	Psychoeducation	Both	30–39	4	3	79	87.67
de Jong-Pleij et al. 2013 (Netherlands)	Mixed Methods	2D Ultrasound	Both	24–39		67		78.7*
de Jong-Pleij et al. 2013 (Netherlands)	Mixed Methods	3D Ultrasound	Both	23–39		66		80.5*
Dhanalakshmi and Nalini 2022 (India)	RCT	MFA Education Psychoeducation Music Touch/Leopold's	Primigravida	18–35	123	128	66.43	77.89
Duanyai et al. 2023 (Thailand)	RCT	Ultrasound	Both	15–19	20	23	75	80.69
Estevao et al. 2025 (Italy)	single-arm feasibility study	Yoga		33		15		49.33*
Estevao et al. 2025 (Italy)	single-arm feasibility study	Yoga		33		15		48.55*
Guney and Ucar 2018 (Turkey)	RCT	Fetal Kick Counts	Both	19–40	55	55	72.25	78.41
Hajure et al. 2025 (Ethiopia)	quasi-experimental	Cognitive Behavioral Therapy	Both	26.12	54	51	21.96	45.9
Kim and Gim 2019 (Korea)	Mixed Methods	Yoga	Both		27	29	61.67	62.89
Kim and Gim 2019 (Korea)	Mixed Methods	MFA Education Psychoeducation	Both		27	27	61.67	68.64
Laurent et al. 2025 (USA)	RCT	Prenatal Education And Mindfulness	Primigravida			81		Combined Prenatal Education and Mindfulness 4.25*
Lee et al. 2023 (Korea)	RCT	Virtual Reality	Both	35	40	40	80.7	81.7
Loughnan et al. 2019 (Australia)	RCT	Cognitive Behavioral Therapy	Both	23–40	33	18	75.62	77.14
Mohapatra et al. 2021 (India)	RCT	Fetal Kick Counts	Primigravida	24–28	86	87	77.57	81.9
Sedgmen et al. 2006 (Australia)	Pretest/Posttest	Ultrasound	Primigravida	18 +		68		80.83*
Karataş Okyay and Güney 2025 (Turkey)	RCT	Touch/Leopold’s	Both	18 +	66	66	71.34	77.33
Thomas et al. 2014 (Austrailia)	Pretest/Posttest	MFA Education Psychoeducation Cognitive Behavioral Therapy	Both	21–45		30		72.2*
Weis and Ryan 2012 (USA)	RCT	Prenatal Education	Both	21–39	36	29	47.23	47.94
Westerneng et al. 2021 (Netherlands)	Pragmatic cluster RCT	Ultrasound	Both	31.58	211	539	77.52	78.23
Zhang et al. 2021 (China)	cross-sectional	Prenatal Education	Both	18–45	118	222	71.63	74.25

^*^Pretest versus posttest (no control)

**Table 5:** Summary of characteristics of included studies using unique tools

Name	Study Design	Intervention	Intervention Age Range	Gravidity	Control N	Intervention N	Control Mean	Intervention Mean
Huang et al. 2025 (USA)	prospective observational cohort	Fetal Kick Counts		Both		1147		.827**
Masroor et al. 2008 (Pakistan)	RCT	Ultrasound	< 40	Both	30	30	30.97	36.66
Sanli et al. 2022 (Turkey)	RCT	Music	25.02	Primigravida	35	35	14.428	13.971
Sanli et al. 2022 (Turkey)	RCT	Music	25.02	Primigravida	35	35	14.228	14.085
Senapati et al. 2023 (India)	Quasi-experimental	Fetal Kick Counts	20–40	Primigravida		40		108.7*
Senapati et al. 2023 (India)	Quasi-experimental	Fetal Kick Counts	20–40	Multigravida		40		97.7*
Shen and Chen 2021 (Taiwan)	RCT	Yoga	23–43	Primigravida	62	61	89.69	94.72
Shen and Chen 2021 (Taiwan)	RCT	Yoga	23–43	Primigravida	62	61	93.96	95.65

^*^Pretest versus posttest (no control) **Posttest only (no control)

Within the 107 articles, there were 77 articles (containing 98 observations) that compared a post-intervention score to a single no intervention score (i.e., a post-only intervention group vs. control-group comparison). These 77 articles included studies that reported the effect at different timepoints as well as having more than one intervention in a study. There were 66 articles that compared pre- and post-scores between an intervention group and a control group (i.e., comparing differences between control and intervention at pre-intervention and post-intervention). There were 55 RCT articles identified, but 5 articles had post-scores only and 5 articles compared two interventions without a non-intervention control group. Of the 55 RCT articles, 51 articles compared pre- and post-scores between an intervention group and a control group (i.e., comparing differences between control and intervention at pre-intervention and post-intervention). Intervention methods tested included fetal movement counting (*n* = 23 articles with 28 observations); touch/Leopold’s maneuver (*n* = 13 articles with 16 observations); music/lullaby/singing (*n* = 18 articles with 24 observations); relaxation techniques (*n* = 21 articles with 26 observations); cognitive therapies (*n* = 11 articles with 20 observations); yoga (*n* = 9 articles with 20 observations); fetal ultrasound visualization (*n* = 11 articles with 26 observations); and education (*n* = 24 articles with 51 observations). Eight articles included male expectant parents with 12 observations. Head-to-head comparisons of interventions were limited (*n* = 12 articles).

### Meta-analyses by study design (Only including pre/post studies with non-intervention control groups)

Post-intervention prenatal attachment scores (observations) were statistically higher (1.46 SD) in the intervention group compared to the control group, independent of study design (N: 107, Hedges g: 1.46, 95% CI: 0.89 to 2.03, p < 0.001, I^2^ = 99.46%). Additionally, the mean difference in prenatal attachment between control and intervention groups was 0.55 standard deviations higher post-intervention compared to pre-intervention, this difference was independent of study design and statistically significant (N: 55, Hedges g: 0.55, 95% CI: 0.36 to 0.73, *p* < 0.001, I^2^ = 96.47%).

**Fig. 2:**
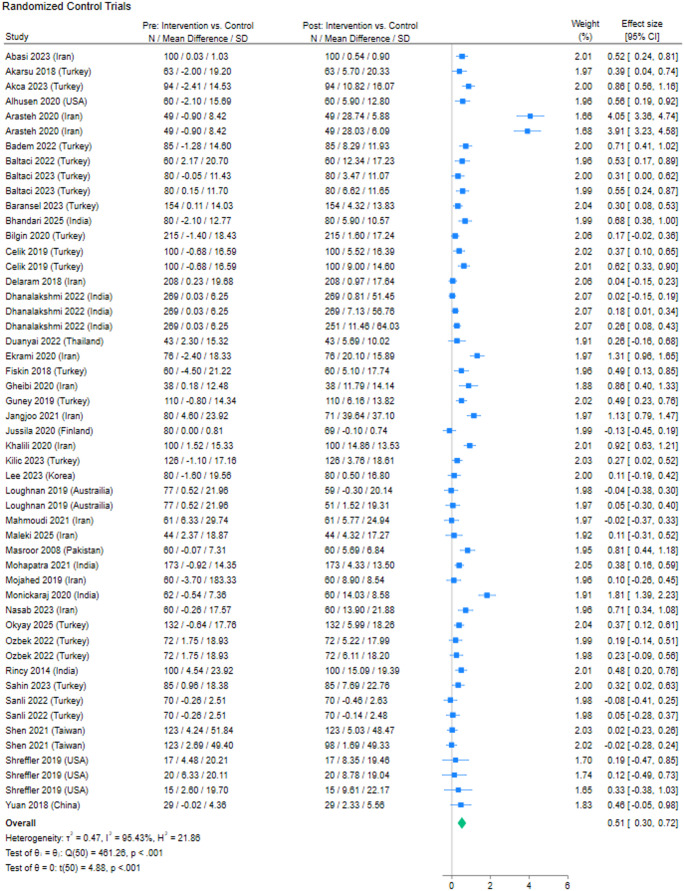
Forest plot: Prenatal attachment differences between the control and intervention groups from pre-intervention to post-intervention from randomized controlled trails

A forest plot showing estimated effect sizes of interventions from 51 RCT observations is shown in Fig. 2. Like the analysis inclusive of all study designs, the mean difference in prenatal attachment between control and intervention groups of the RCT observations was 0.51 standard deviations higher post-intervention compared to pre-intervention and this difference was statistically significant (N: 51, Hedges g: 0.51, 95% CI: 0.30 to 0.72, p < 0.001, I^2^ = 95.53%; Fig. 2).

### Meta-analysis by intervention method (Fig. 3A-E, Supplemental Fig. 2, and Table 6)

**Fig. 3:**
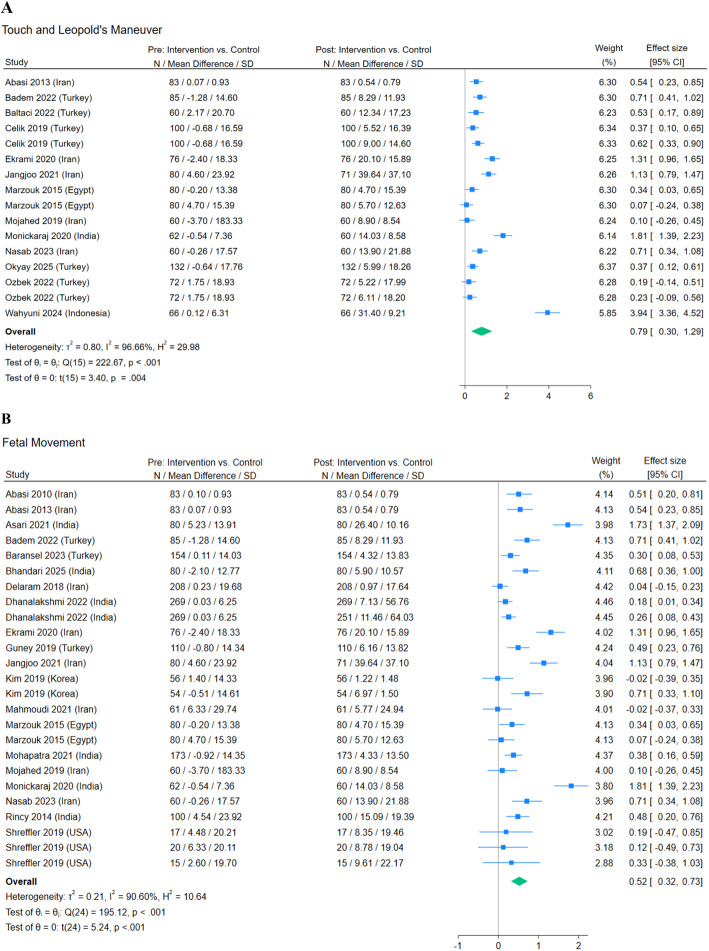

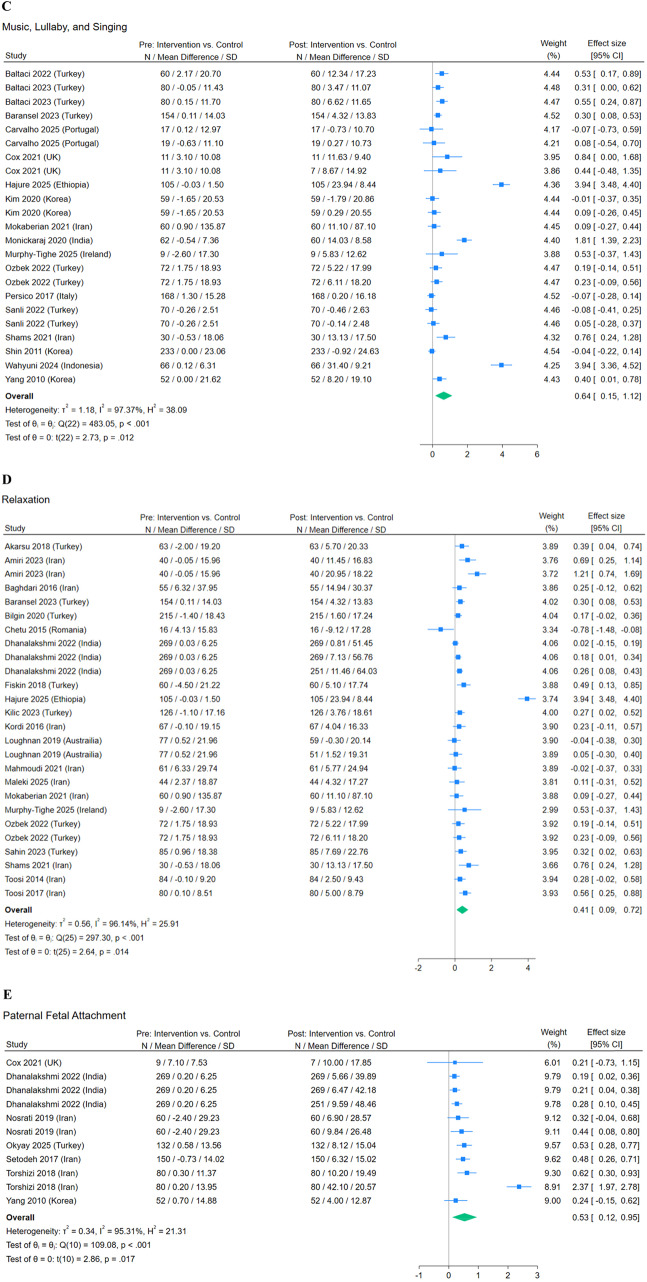
Forest plots: prenatal attachment differences between the control and intervention groups from pre-intervention to post-intervention by interventions

**Table 6:** Meta-analysis: maternal fetal attachment differences between control and intervention from pre-intervention to post-intervention by intervention type

	N	Hedges’ g [95% CI]	*p*	I^2^
Touch/Leopold's maneuver	16	0.80 [0.30, 1.29]	.004	96.66
Fetal movement	25	0.52 [0.32, 0.73]	<.001	90.60
Music/lullaby/singing	23	0.64 [0.15, 1.12]	.012	97.37
Relaxation	26	0.41 [0.09, 0.72]	.014	96.14
CPT/CBT	9	1.66 [0.31, 3.01]	.022	98.44
Yoga	7	0.28 [−0.03, 0.59]	.067	73.01
Meditation	11	0.42 [0.18, 0.65]	.003	57.56
Breathing	16	0.33 [0.16, 0.52]	.001	70.76
Ultrasound	8	0.20 [−0.09, 0.50]	.147	81.98
Education	41	0.74 [0.38, 1.11]	<.001	97.78

*CI* confidence interval. *CPT* cognitive processing therapy. *CBT* cognitive behavioral therapy

The methods, length, timing, and number of interventions within categories varied (see Supplemental Fig. 3). We separated each article into categories of interventions based on the methods described.

### Touch and Leopold’s maneuver

Thirteen articles (16 observations) that included teaching or performing Leopold’s maneuver or instructing a person to touch their abdomen were included in this category. In touch and Leopold’s maneuver interventions, the mean difference in prenatal attachment between control and intervention groups was 0.80 standard deviations higher post-intervention compared to pre-intervention; this difference was statistically (N: 16, Hedges g: 0.80, 95% CI: 0.30 to 1.29, *p* = 0.004, I^2^ = 96.66%; Fig. 3A, Table 6).

### Fetal movement

Twenty articles (25 observations) that included teaching, recording, paying attention to, or counting fetal movements were included in this category. Fetal movement interventions had a mean difference in prenatal attachment between control and intervention groups of 0.52 standard deviations higher post-intervention compared to pre-intervention; this difference statistically significant (N: 25, Hedges g: 0.52, 95% CI: 0.32 to 0.73, p < 0.001, I^2^ = 90.60%; Fig. 3B, Table 6).

### Music, lullaby and singing

Seventeen articles (23 observations) that included listening to, writing, or singing music, songs or lullabies were included in this category. Music, lullaby, and singing interventions had a mean difference in prenatal attachment between control and intervention groups of 0.64 standard deviations higher post-intervention compared to pre-intervention; this difference was statistically significant (N: 23, Hedges g: 0.64, 95% CI: 0.15 to 1.12, *p* = 0.012, I^2^ = 97.37%; Fig. 3C, Table 6).

## Relaxation

Twenty-one articles (26 observations) that included teaching or instructing any form of relaxation techniques were included in this category. Articles were included in the category if they specifically identified relaxation techniques within their methods. Relaxation interventions had a mean difference in prenatal attachment between control and intervention groups of 0.41 standard deviations higher post-intervention compared to pre-intervention; this difference was statistically significant (N: 26, Hedges g: 0.41, 95% CI: 0.09 to 0.72, *p* = 0.014, I^2^ = 96.14%; Fig. 3D, Table 6).

### Cognitive therapies (CPT/CBT)

Cognitive therapies are psychotherapies that challenge negative thoughts and/or help treat mood disorders (Beck and Dozois 2011). Six articles (9 observations) that categorized the intervention as a cognitive therapy were included in this category. None of the cognitive therapies were the same. CPT/CBT interventions had a mean difference in prenatal attachment between control and intervention groups of 1.66 standard deviations higher post-intervention compared to pre-intervention; this difference was statistically significant (N: 9, Hedges g: 1.66, 95% CI: 0.31 to 3.01, *p* = 0.022, I^2^ = 98.44%; Supplemental Fig. 2, Table 6).

### Yoga

Five articles (7 observations) identified teaching, performing, and tracking some type of yoga as a part of their intervention and were included in this category. Yoga interventions had a mean difference in prenatal attachment between control and intervention groups of 0.28 standard deviation higher post intervention compared to pre-intervention independent of study design; this difference was not statistically significant (N:7, Hedges g: 0.28, 95% CI: −0.03 to 0.59, *p* = 0.066, I^2^ = 73.01%; Supplemental Fig. 2, Table 6).

### Meditation

Meditation to direct concentration and focus of attention has been widely studied for a variety of health and behavioral outcomes (Matko and Sedlmeier 2019). Eight articles (11 observations) that identified meditation as one of the techniques within their intervention were included in this category. Meditation includes concentration and focus of attention for a variety of purposes. Meditation interventions had a mean difference in prenatal attachment between control and intervention groups of 0.42 standard deviation higher post-intervention compared to pre-intervention; this difference was statistically significant (N: 11, Hedges g: 0.42, 95% CI: 0.18 to 0.65, *p* = 0.003, I^2^ = 57.56%; Supplemental Fig. 2, Table 6).

### Breathing

Ten articles (16 observations) that identified teaching breathing techniques (i.e., diaphragmatic breathing) within their intervention were included in this category. While many interventions may utilize breathing techniques, we only included articles in this category if they specifically mentioned this in their methods. Breathing interventions had a mean difference in prenatal attachment between control and intervention groups of 0.34 standard deviations higher post-intervention compared to pre-intervention; this difference was statistically significant (N:16, Hedges g: 0.34, 95% CI: 0.16 to 0.52, *p* = 0.001, I^2^ = 70.76%; Supplemental Fig. 2, Table 6).

### Ultrasound

Seven articles (8 observations) included some type of fetal ultrasonography within their intervention and were included in this category. Ultrasonography interventions had a mean difference in prenatal attachment between control and intervention groups of 0.20 standard deviation higher post-intervention compared to pre-intervention independent of study design; this difference was not statistically significant (N: 8, Hedges g: 0.20, 95% CI: −0.09 to 0.50, *p* = 0.147, I^2^ = 81.98%; Supplemental Fig. 2, Table 6).

### Education

Thirty-one articles (41 observations) identified their intervention as training or educating pregnant patients and/or partners on attachment behavior. All of these “attachment education” interventions utilized multiple methods whose purpose was to improve bonding within the pregnancy. None of these education interventions were the same. Education interventions had a mean difference in prenatal attachment between control and intervention groups of 0.74 standard deviation higher post-intervention compared to pre-intervention; this difference was statistically significant (N: 41, Hedges g: 0.74, 95% CI: 0.38 to 1.11, *p* < 0.001, I^2^ = 97.78%; Supplemental Fig. 2, Table 6).

### Meta-analyses by intervention target

Following an intervention, attachment scores in non-pregnant expectant male partners were 0.88 standard deviations higher in the intervention cohort compared to the control cohort; this difference was statistically significant (N:12, Hedges g: 0.88, 95% CI: 0.22 to 1.52, *p* < 0.001, I^2^ = 95.94). The mean difference in scores between control and intervention groups was 0.53 standard deviations higher post-intervention compared to pre-intervention; this difference was statistically significant (N: 11, Hedges g: 0.53, 95% CI: 0.11 to 0.95, *p* = 0.017, I^2^ = 95.31%; Fig. 3E).

### Meta-regressions (Table 1, bottom, and Tables 7 and 8)

**Table 7:** Meta-regression: associations between study characteristics and maternal fetal attachment differences between control and intervention form pre-intervention to post-intervention

	N	Estimate [95% CI]	p	R^2^
Randomized Control Trial	84	−0.08 [−0.44, 0.29]	.678	0.00
Intervention				
Sole Intervention	84	−0.24 [−0.59, 0.12]	.195	0.60
Number of Interventions	84	0.09 [−0.06, 0.25]	.244	0.45
Duration of intervention	83	0.01 [0.00, 0.02]	.012	5.64
Follow-up time	82	0 [−0.00, 0.01]	.052	2.92
Maternal Age				
Pooled Average	54	0.01 [−0.03, 0.04]	.754	0.00
Difference between Intervention and Control	52	−0.08 [−0.24, 0.07]	.296	0.28
Estimated Gestational Age				
Pooled Average	35	−0.02 [−0.08, 0.04]	.500	0.00
Difference between Intervention and Control	35	0.03 [−0.20, 0.25]	.830	0.00
Married	44	1.92 [−0.27, 4.12]	.086	5.01
Planned	37	0.45 [−0.21, 1.11]	.178	5.21
Known Sex	14	0.39 [−0.80, 1.59]	.517	0.00
Insufficient Income	22	−0.05 [−0.25, 0.15]	.614	0.00
Employed	58	−0.51 [−0.90, −0.11]	.013	8.83
High School Educated	60	−0.34 [−0.81, 0.12]	.147	1.77
Abortion	4	0.29 [−1.23, 1.81]	.709	0.00
Religious	4	−0.29 [−1.15, 0.56]	.503	0.00
Natural	14	0.03 [−1.16, 1.22]	.965	0.00
Satisfied Spouse	10	0.10 [−1.85, 2.06]	.916	0.00
Primigravid	33	−3.90 [−6.89, −0.91]	.010	15.37

*CI* confidence interval

**Table 8:** Meta-regression by intervention: associations between sole intervention status and duration of intervention in maternal fetal attachment scores between control and intervention from pre-intervention to post-intervention

	*N*	Estimate [95% CI]	*p*	R^2^
Touch/Leopold's maneuver				
Sole Intervention	16	−0.42 [−1.59, 0.74]	.476	0.00
Duration of intervention	16	0.03 [0.01, 0.05]	.002	37.30
Fetal movement				
Sole Intervention	25	0.11 [−0.32, 0.55]	.613	0.00
Duration of intervention	25	−0.00 [−0.01, 0.01]	.558	0.00
Music/lullaby/singing				
Sole Intervention	23	−0.76 [−1.72, 0.20]	.121	6.10
Duration of intervention	23	0.02 [0.00, 0.04]	.015	18.66
Relaxation				
Sole Intervention	26	−0.08 [−0.92, 0.75]	.843	0.00
Duration of intervention	25	0.00 [−0.01, 0.02]	.522	0.00

*CI* confidence interval

Meta-regression results were presented in Table 7 and 8. Most study- and patient-specific characteristics were not associated with differences on prenatal attachment (Table 7). However, employment status showed a significant effect; a higher proportion of employed individuals in the intervention group was associated with a smaller difference in prenatal attachment compared to the control group. Specifically, an intervention group with twice as many employed than unemployed participants compared to an equally employed control group showed a 0.51 standard deviations smaller difference in prenatal attachment, this difference was statistically significant (N: 58, Estimate: −0.51, 95% CI: −0.90 to −0.11, *p* = 0.013, R^2^ = 8.83%; Table 7). Also, duration of intervention showed a significant effect; a longer intervention was associated with a greater difference in prenatal attachment between intervention and control groups. Specifically, a 1-day increase in intervention duration was associated with a 0.01 standard deviations larger difference in prenatal attachment (N: 83, Estimate: 0.01, 95% CI: 0.00 to 0.02, *p* = 0.012, R^2^ = 5.64%; Table 7). Primigravid status also showed a significant effect; a higher proportion of primigravid individuals in the intervention group was associated with a smaller difference in prenatal attachment compared to the control group. Specifically, an intervention group with twice as many primigravid patients showed a 3.90 standard deviations smaller difference in prenatal attachment (N: 33, Estimate: −3.90, 95% CI: –6.89 to −0.91, *p* = 0.010, R^2^ = 15.37%; Table 7).

Intervention-specific meta-regression analyses showed that sole intervention status was not associated with differences in prenatal attachment among touch and Leopold’s maneuver, fetal movement, music, lullaby, and singing, and relaxation interventions (Table 8). Meta-regression analyses for fetal movement and relaxation interventions revealed that duration of intervention did not have significant impact on prenatal attachment scores (Table 8). In contrast, for touch and Leopold’s maneuver, duration of intervention showed a significant effect with a longer intervention associated with a greater difference in prenatal attachment between intervention and control groups. Specifically, a 1-day increase in intervention duration was associated with a 0.03 standard deviations larger difference in prenatal attachment (N: 16, Estimate: 0.03, 95% CI: 0.01 to 0.05, *p* = 0.002, R^2^ = 37.30%; Table 8). Likewise, for music, lullaby, and singing interventions, duration of intervention showed a significant effect with a longer intervention associated with a greater difference in prenatal attachment between intervention and control groups. Specifically, a 1-day increase in intervention duration was associated with a 0.02 standard deviations larger difference in prenatal attachment (N: 23, Estimate: 0.02, 95% CI: 0.00 to 0.04, *p* = 0.015, R^2^ = 18.66%; Table 8).

## Risk of bias/publication bias

Estimates of risk of bias and publication bias are shown in Supplemental Figs. 4 A and 4B. We minimized publication bias by including multiple language articles, yet there was significant asymmetry across the board. The asymmetry throughout the funnel plots for different interventions may be related to methodological diversity or the degree of heterogeneity noted in the different methods of intervention.

The hierarchy of the risk of bias within studies decreases from RCTs, to non-RCTs to cohort studies and then cross-sectional studies (Sargeant et al. 2022). While many of the studies were RCTs, around 30% of those had overall low risk of bias and over 50% had high risk of bias due to deviations from intended interventions and selection of the reported results. None of the non-randomized studies of interventions had low risk of bias and less than 20% had high risk of bias.. The degree of bias especially within the RCTs should be interpreted carefully as the degree of heterogeneity was large. Despite this, random effects meta-analyses accounted for the heterogeneity in the overall meta-analysis and the meta-analyses by intervention.

## Discussion

Intense interest in the malleability of prenatal attachment as a preventive intervention has exceeded the capacity of traditional meta-analyses to synthesize findings. The current review complements and extends knowledge from prior reviews through the deliberate inclusion of intervention studies employing various study designs and testing a variety of intervention methods alone and in combination (Deaton and Cartwright 2018). Viewed collectively, intervention was associated with a significant increase in prenatal attachment scores among both pregnant and non-pregnant expectant parents of both sexes. Unfortunately, our conclusions need to be tempered due to the number of small studies, higher level risk of bias, variability in intervention protocols and our inability to completely explain the degree of heterogeneity.

### Impact of between-study differences on estimated effects

Our initial meta-analysis included all RCTs, non-RCTs and observational studies in the literature, yet this did not influence the associations between study characteristics and maternal fetal attachment differences between control and intervention groups from pre-intervention to post-intervention. Our inclusive approach showed that the clinical implications of the interventions remained consistent despite the high heterogeneity between studies. Importantly, results and clinical implications were similar between the meta-analysis using all study designs and the meta-analysis using only randomized control trails. Clinical implications remained consistent despite the high heterogeneity between studies or the implicit bias in non-RCT studies.

Meta-regressions aimed to clarify what characteristics did or did not contribute to potential heterogeneity in effect sizes. RCT versus other design, parental age, estimated gestational age at intervention, duration of intervention, number of interventions used concomitantly, time to follow-up, marital status, income, education, gravidity/parity, religion, history of abortion, fertility treatments or marital satisfaction did not contribute to the heterogeneity. Because observational research like case–control and cohort studies provide valuable insights on non-invasive highly acceptable interventions finding about these studies from this review inform obstetricians when addressing questions by patients about these activities. Additionally, because employment status may have been responsible for some of the heterogeneity this factor needs to be included in future research.

### Particularly promising interventions and recommendations for future research

While any intervention utilized to improve prenatal attachment may improve outcomes, true head-to-head randomized controlled trials are desperately needed to inform clinicians going forward. Despite a dearth of head to head comparisons, we found eight articles (Sedgmen et al. 2006; de Jong-Pleij et al. 2013; Kim and Gim 2019; Shreffler et al. 2019; Coté et al. 2020, 2023; Baltacı et al. 2023; Lee et al. 2023) that prospectively compared different interventions yet one of these was not a randomized controlled trial (Kim and Gim 2019). Despite the limitations on our findings, we have identified a few promising interventions that warrant further study.

### Fetal movement

Fetal movement counting in pregnancy is a frequently studied intervention. Utilizing fetal movement counting to improve prenatal attachment has its origins in the natural progression of prenatal attachment scores over the course of pregnancy. Fetal quickening (the time a pregnant patient starts feeling fetal movements) around 18–22 weeks correlates to a time when prenatal attachment scores naturally start to increase from the first into the second trimester (Close et al. 2020). While it is unclear if counting movements reduces perinatal mortality (Bellussi et al. 2020), our pooled intervention analysis did show improved prenatal attachment scores compared to control groups. As an intervention, in part or in total, fetal movements accounted for approximately one third of the articles reviewed.

A prior meta-analysis by Abasi and colleagues concluded that there was no significant impact on fetal movement counting in relation to prenatal attachment scores compared to not counting movements (MD = 0.36; 95% CI = − 0.23–0.95; p = 0.23); however, another meta-analysis by AlAmri and Smith (2022) did find a statistically significant improvement in prenatal attachment scores with fetal movement counting compared to not counting (SMD = 0.72; CI = 0.10–1.33; p = 0.02). Most fetal movement interventions encouraged once per day or multiple times a day tracking of the fetus, yet adherence to the intervention was rarely commented on, and none of the articles performed a per protocol versus intention to treat analysis. Regardless, the biologic probability and correlation to how recognizing fetal movements corresponds naturally to an increase in bonding over the course of a pregnancy necessitates a focus on this as an intervention going forward.

### Music, lullaby and singing

Music is universal and varies more within than between societies (Mehr et al. 2019). This fact clearly supports a focus on music as an intervention. Listening to music, singing, and playing instruments, evoke unique brain responses influenced by individual traits and musical attributes (Ding et al. 2024). While we found a statistically significant effects size within music interventions, the pooled effects were heterogeneous. Neither a sole intervention status nor duration of interventions had a significant impact on prenatal attachment scores or explained the heterogeneity. The medium or genre of music was different between the studies and could explain some of the heterogeneity. This necessitates head-to-head comparisons to evaluate the differences between the mediums of music (singing, listening, playing etc.) or genres of music (lullaby, classical etc.). Baltaci et al. (2023) did just that by comparing listening to lullabies to listening to music in general to a control group. Listening to lullabies and music improved prenatal attachment scores statistically more than the control group, however the lullaby intervention had higher prenatal attachment scores than the music intervention (78.07 ± 5.7 vs 74.92 ± 5.41). While this is encouraging, larger studies in more diverse populations will need to be performed before true conclusions can be made.

### Relaxation

Anxiety and stress have been shown to negatively impact prenatal attachment (Göbel et al. 2018; Şanlı and Akbağ 2022) and research has looked at ways to decrease these constructs through relaxation techniques. There are multiple relaxation techniques ranging from breathing exercises to mindfulness meditation to progressive muscle relaxation. Not only did relaxation techniques in our study show statistically significant higher prenatal attachment but they had the lowest amount of heterogeneity (albeit moderate) compared to all the other interventions. This may mean that it is the effect on the stress or anxiety which moderates the improvement in prenatal attachment. Either way, there were no head-to-head comparisons nor were there any comparisons between individuals who had high levels of anxiety or stress to those that did not. More research on relaxation interventions that focuses on head-to-head comparisons would be needed to be able to clarify these connections.

### Ultrasonography

A previous meta-analytic study was utilized to direct future research and identified 14 predictors of prenatal attachment in pregnant individuals. While gestational age had a moderate to substantial effect size, social support, and prenatal testing (ultrasonography) also had moderate effect sizes (Yarcheski et al. 2009).

While our study did not find a statistically significant effect size within the ultrasound intervention, this may be due to the ubiquitous nature of ultrasonography within pregnancy (patients universally undergo ultrasounds in pregnancy as a standard of care) and the difficulty of having a non-intervention control group. Additionally, the type of ultrasound performed (3D versus 2D) or the gestational age when it was performed may alter the effect size. In fact, many of the studies not included in the meta-analysis by intervention surrounded the use of ultrasonography.

Both Sedgman et al. (2006) and de Jong-Pleij et al. (2013) examined 2D versus 3D ultrasounds and found a statistically increased prenatal attachment score after both interventions yet did not find a statistically significant difference between the two interventions (i.e., 2D ultrasounds increased prenatal attachment scores equally to 3D ultrasounds). Interestingly, a small meta-analysis found that the standard mean difference in effects size for prenatal attachment scores of 3D versus 2D ultrasounds favored the 3D ultrasounds (p = 0.02) (Coté et al. 2023).

Some studies examined the impact of ultrasonography in addition to other technologies to enhance the experience. Lee et al. (2023) examined 3D ultrasonography with virtual reality (VR) in a phone app to 3D ultrasonography and the same phone app without VR images and did not find a statistically significant difference between the groups as far as global scores after the interventions (81.7 ± 7.3 versus 80.7 ± 7.3; p = 0.52). Although both groups appear to have higher MFA scores after the interventions, it is unknown if either group statistically increased those scores. Coté et al (2020), examined 3D printed models created from 3D ultrasonography to 3D ultrasonography alone, and found that the 3D-printed model group increased prenatal attachment more than the group with ultrasonography alone (p = 0.002) (Coté et al. 2020). The significant findings, universal applications of ultrasonography yet small numbers highlight the importance of focusing on ultrasonography in future trials moving forward.

### Paternal fetal attachment

The scarcity of intervention studies enrolling non-pregnant expectant parents also renders findings difficult to interpret. Prenatal attachment scores tend to be lower in male expectant parents relative to their pregnant counterparts and do not statistically increase over the course of a pregnancy independent of an intervention (Close et al. 2020). While there was a statistically significant mean difference in scores between control and intervention groups post-intervention compared to pre-intervention the degree of heterogeneity was high. Determination of the relative effectiveness of one intervention to another was impossible due to the small number of observations (*n* = 12) and studies (*n* = 8). Summarily, inclusion of fathers and non-pregnant expectant parents and other caregivers is strongly recommended in future research to reflect the heterogeneity in children’s early caregivers and extend evidence for the role of sex-specific hormones and spousal support in prenatal attachment (Coté et al. 2024; Unal and Senol 2024).

While prenatal attachment intervention research that includes non-pregnant expectant parents is minimal (de Waal et al. 2025), it was entirely absent for non-pregnant expectant parents who are female or non-binary, reflecting the resilience of conventionally assigned gender roles in scientific discourse (Vo et al. 2024). This is detrimental to all families since fathers, non-pregnant female expectant parents, and non-parent caregivers frequently share childrearing responsibilities (Suzuki et al. 2022; Ettenberger et al. 2024) and face similar psychological stressors as pregnant females (Schöch et al. 2024). Research on prenatal attachment that reflects the heterogeneity of early life caregivers is optimal for child and family health and is an important reason it should be focused on going forward.

### Other interventions

Many of the studies utilized interventions that were tangentially related or based on a guiding principle but would in themselves be unique to the individual study. For example, the Mothers and Babies Course (Alhusen et al. 2021) and the MUMentum Pregnancy program (Loughnan et al. 2019) utilized these unique CBT programs as interventions. This is an obvious contributor to the heterogeneity and enhances the need for more head-to-head randomized controlled trials. These examples highlight that psychological constructs like depression have been shown to negatively affect prenatal attachment (Testouri et al. 2023). We have not yet teased out the underlying connection that can separate if an intervention uniquely improves prenatal attachment or if improvement in prenatal attachment scores is a function of improving depression or other constructs. Additionally, while we applaud previous research involved in evaluating education interventions surrounding prenatal attachment, we feel that going forward a standardized protocol agreed upon by stakeholders in this field would optimize results, improve conclusions, and allow for easier adoption by clinicians going forward.

Finally, while we shouldn’t eliminate potential intervention candidates in the prenatal attachment space, the sheer number of possible programs that can be utilized should be narrowed down prior to any specific recommendation. For example, social support (Yarcheski et al. 2009) or mindfulness interventions (Sansone et al. 2024a, b; Borelli et al. 2023) have shown promise in improving attachment yet have either not been codified as a prenatal attachment intervention or have not been examined in an RCT head-to-head with other prenatal attachment interventions.

### Limitations

Findings should be interpreted within the context of study limitations. The first concerns the inconsistency in demographic data reported in studies. Second, we were unable to draw conclusions about the relative effectiveness of different types of studies because many studies tested multiple interventions concomitantly, there were few studies involving head-to head comparisons, and there was a high percentage of variation across studies that suggests inconsistency of studies’ results. Third, there was a paucity of studies that included non-pregnant expectant parents. Fourth, our search engines did not include any non-English databases, and limited databases that capture grey literature (Trip and Google Scholar). While no systematic review and meta-analysis can include every search engine available, the use of different search engines may affect the number of articles found and potentially the effect sizes shown with the included studies.. Fifth, with Artificial Intelligence (AI) translations or individual translators there can be inconsistencies that could be eliminated with evidence-based translation protocols. Finally, we are unable to evaluate mechanisms by which interventions influenced attachment scores since validation studies have not been conducted. Lack of clarity about change mechanisms is a pervasive short coming in intervention research not limited to research on attachment (Sheeran et al. 2017). Regardless, the far-reaching impact of prenatal attachment justifies future research that examines and validates change mechanisms to ensure the optimal allocation of public resources for public health benefit.

## Conclusion

In the context of promising findings about the malleability of prenatal attachment via a variety of non-invasive strategies, future research including head-to-head comparisons and non-pregnant expectant parents and non-parental caregivers are needed. The theoretical framework of attachment theory may help guide comparisons in the future and we should address differences between interventions with research. In particular, the mechanisms behind interventions may help guide the development of standardized evidence-based approaches. Our review suggests focusing on fetal movement counting, ultrasound pictures or models, relaxation, and music/lullaby/singing interventions. While this research suggests that multiple interventions can improve bonding, the low quality of evidence and degree of heterogeneity necessitates the need for future research to standardize the intervention utilized to allow for more reproducibility and accuracy when analyzing outcomes. Continued stimulus to an intervention may affect longevity but not the degree of the improvement and this needs to be a focus going forward. Finally, to create a transparent view of an interventions efficacy and effectiveness future studies need to present per protocol (for full adherence) and intention to treat (for preservation of randomization) results.

## Supplementary Information

Below is the link to the electronic supplementary material.

Supplementary file1 (DOCX 15 KB)

Supplementary file2 (DOCX 746 KB)

Supplementary file3 (DOCX 95 KB)

Supplementary file4 (DOCX 381 KB)

Supplementary file5 (DOCX 306 KB)

## Data Availability

All data used in analysis can be seen at 10.5281/zenodo.13484508.

## Footnotes

1 While the terms “maternal fetal attachment” and “paternal fetal attachment” are prevalent in existing literature, we attempted to adopt gender neutral terms as was practicable herein, to acknowledge the diversity of gender identities and roles independent of biological sex at birth. Prenatal attachment is used to refer to attachment to the fetus by an expectant parent with specification of the pregnant versus non-pregnant expectant parent as needed. We further emphasize that the term “parent” is imperfect, and not meant to conflate pregnancy with parental desire, role attainment, or identification, also known to show substantial variability between individuals, and within individuals between different pregnancies (Level et al. 2024). Finally, we use the term “prenatal attachment” to describe the parental fetal relationship, recognizing that some attachment researchers use bonding to the parental fetal relationship and reserve “attachment” for describing postpartum parent–child relationships (Brandon et al. 2009; Redshaw and Martin 2013).
