# Supplementary material for: Prenatal attachment interventions: a comprehensive systematic review and meta-analysis
Source: Arch Womens Ment Health. 2025 Nov 8;28(6):1447–71. doi: 10.1007/s00737-025-01630-w (PMC12702810; doi:10.1007/s00737-025-01630-w)
Supplement: Supplementary file 1 — Supplementary file1 (DOCX 15 KB) [file 737_2025_1630_MOESM1_ESM.docx]

**Supplemental Figure 1 Synthesis Measures**

**Primary MFA meta-analysis**

1. Difference in MFA between control and intervention at post-intervention only (all study designs)
2. Difference in MFA between control and intervention from pre- to post-intervention (all study designs)
   1. Meta-regression: RCT status, follow-up time, type of intervention (single or combined intervention technique), number of inventions, intervention duration, maternal age, gestational age, marital status, planned pregnancy, knowledge of baby sex, income level, employment status, education level, history of abortion, religious affiliation, natural concept, spouse satisfaction, and primigravid.
3. Difference in MFA between control and intervention from pre- to post-intervention in randomize control trials only (RCT only).

**Secondary MFA meta-analysis by type of intervention**

1. Difference in MFA between control and intervention from pre- to post-intervention for touch/Leopold’s maneuver interventions (all study designs)
   1. Meta-regression: whether for touch/Leopold’s maneuver was the sole intervention and the duration of the intervention.
2. Difference in MFA between control and intervention from pre- to post-intervention for fetal movement interventions (all study designs)
   1. Meta-regression: whether fetal movement was the sole intervention and the duration of the intervention.
3. Difference in MFA between control and intervention from pre- to post-intervention for music, lullaby, and singing interventions (all study designs)
   1. Meta-regression: whether for music/lullaby/singing was the sole intervention and the duration of the intervention.
4. Difference in MFA between control and intervention from pre- to post-intervention for relaxation interventions (all study designs)
   1. Meta-regression: whether for relaxation was the sole intervention and the duration of the intervention.
5. Difference in MFA between control and intervention from pre- to post-intervention for cognitive processing therapy and cognitive behavioral interventions (all study designs)
6. Difference in MFA between control and intervention from pre- to post-intervention for yoga interventions (all study designs)
7. Difference in MFA between control and intervention from pre- to post-intervention for mediation interventions (all study designs)
8. Difference in MFA between control and intervention from pre- to post-intervention for breathing interventions (all study designs)
9. Difference in MFA between control and intervention from pre- to post-intervention for ultrasound interventions (all study designs)
10. Difference in MFA between control and intervention from pre- to post-intervention education interventions (all study designs)

**PFA Analysis**

1. Difference in PFA between control and intervention at post-intervention only (all study designs)
2. Difference in PFA between control and intervention from pre- to post-intervention (all study designs)
