# Supplementary material for: Prenatal attachment interventions: a comprehensive systematic review and meta-analysis
Source: Arch Womens Ment Health. 2025 Nov 8;28(6):1447–71. doi: 10.1007/s00737-025-01630-w (PMC12702810; doi:10.1007/s00737-025-01630-w)

**Supplemental Figure 2** Prenatal attachment differences between the control and intervention groups from pre-intervention to post-intervention by interventions


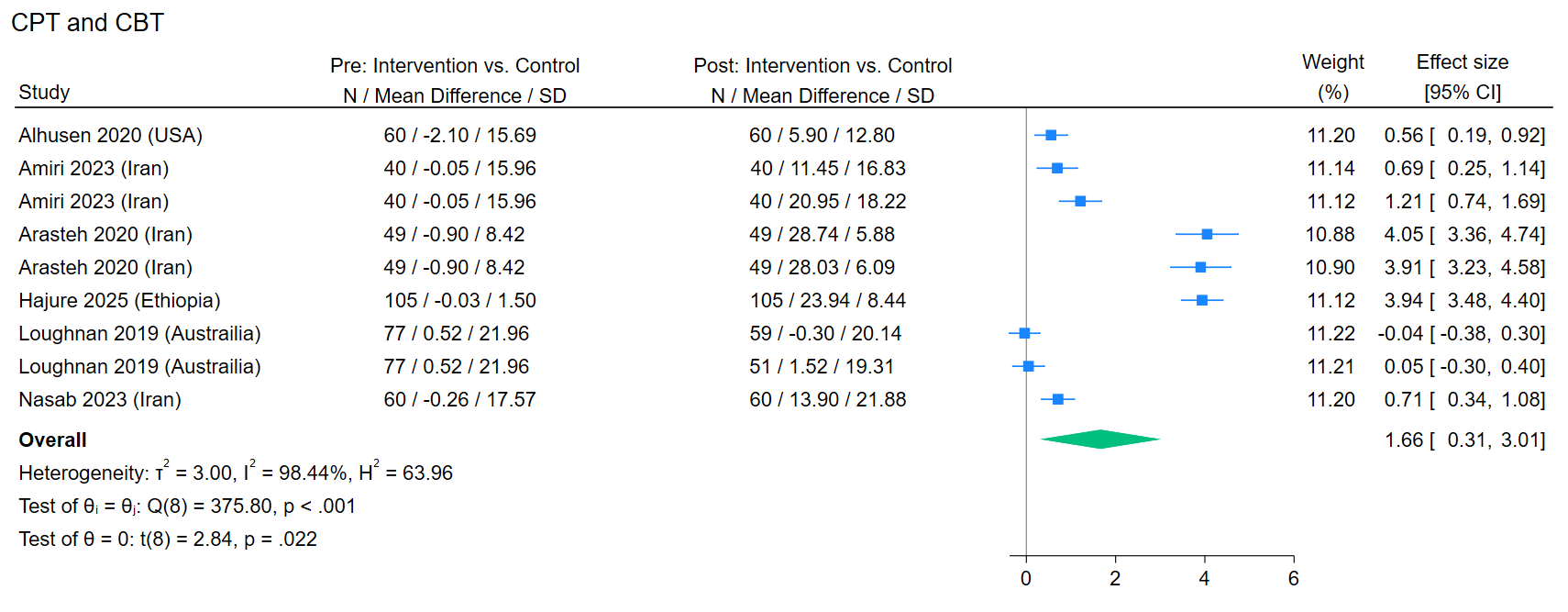


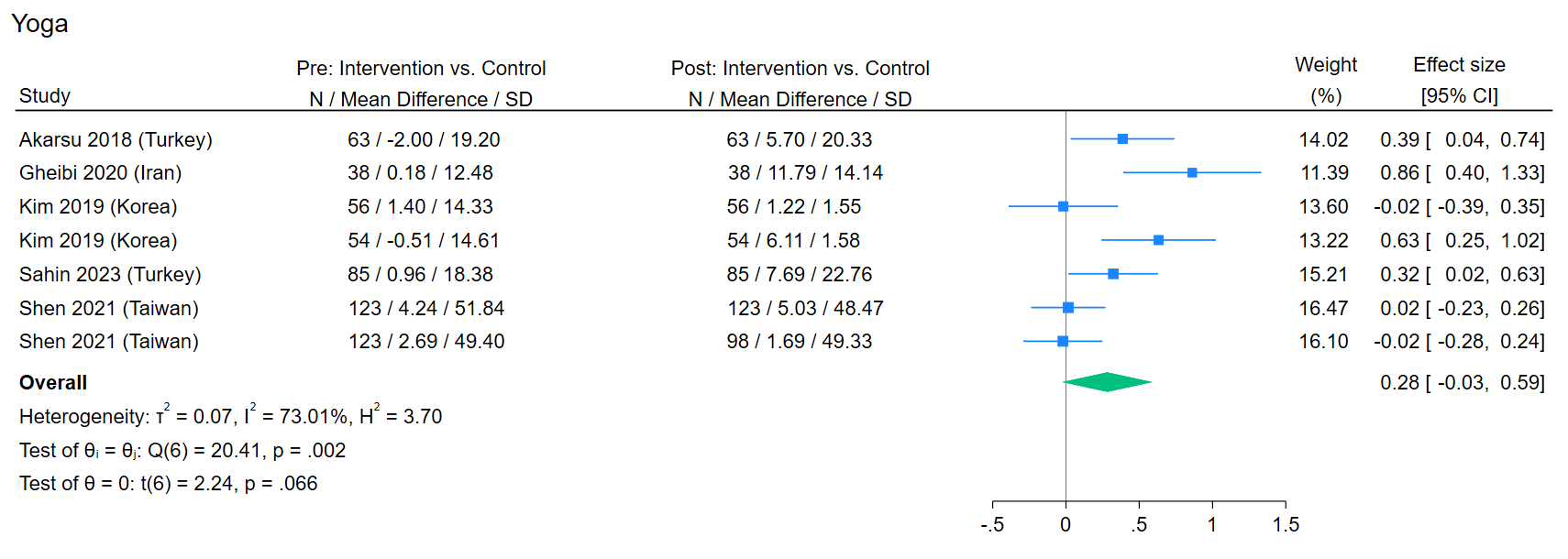


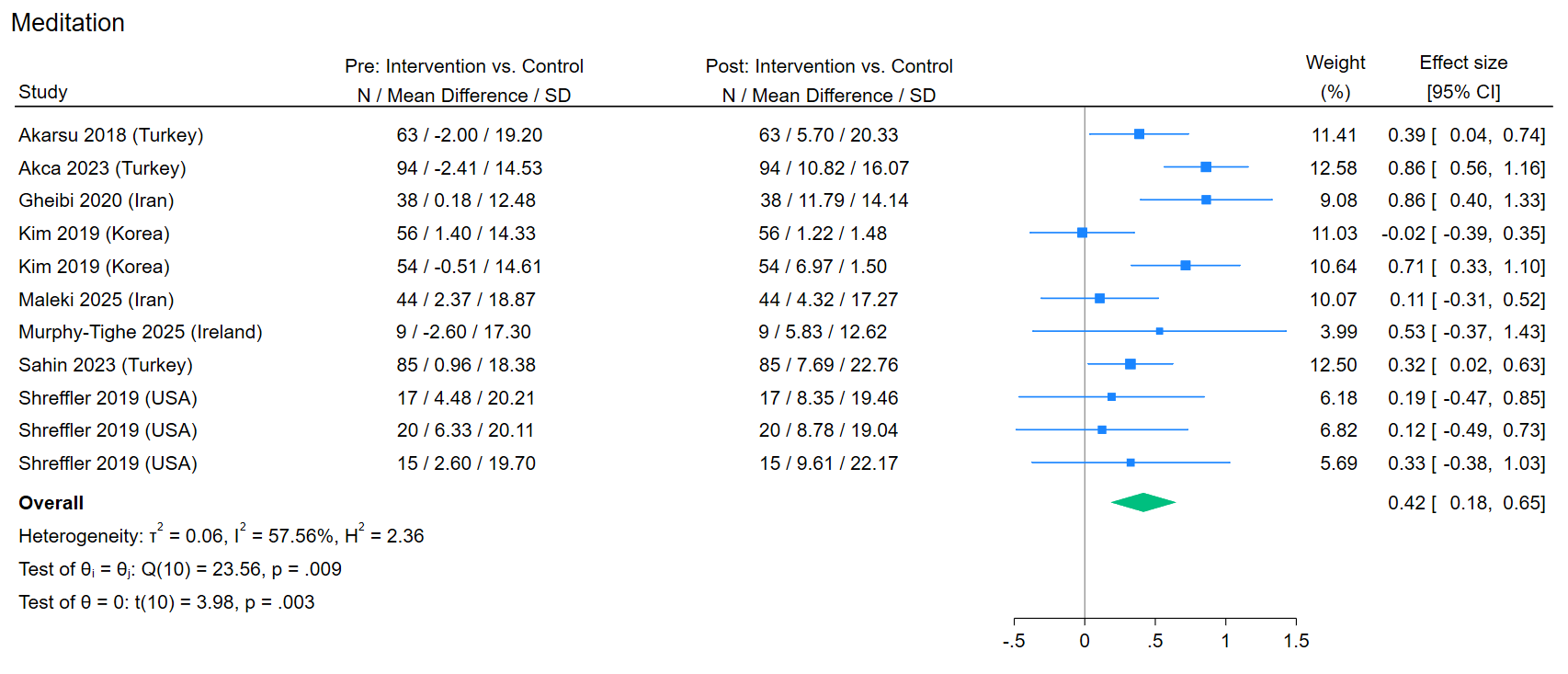


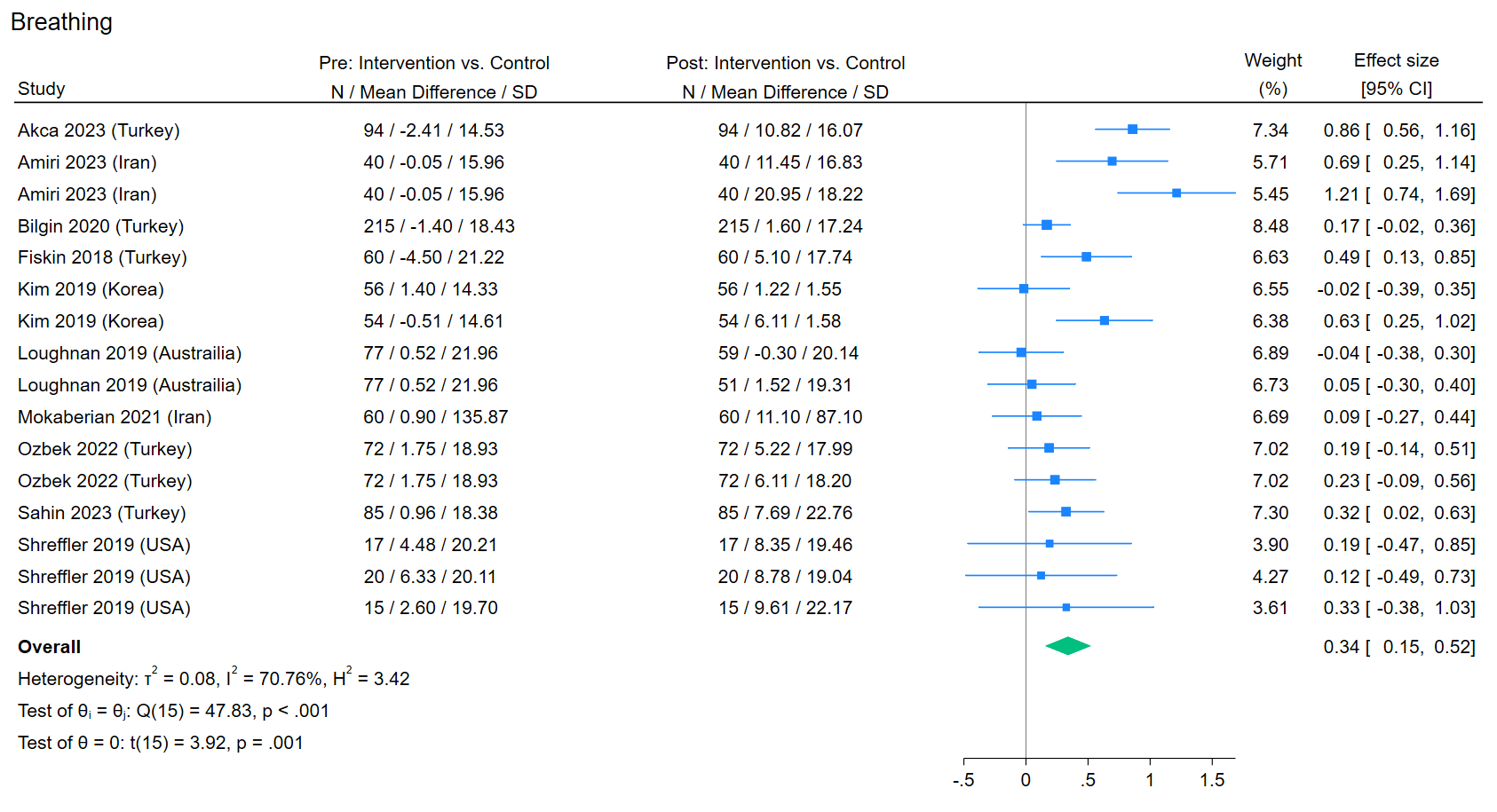


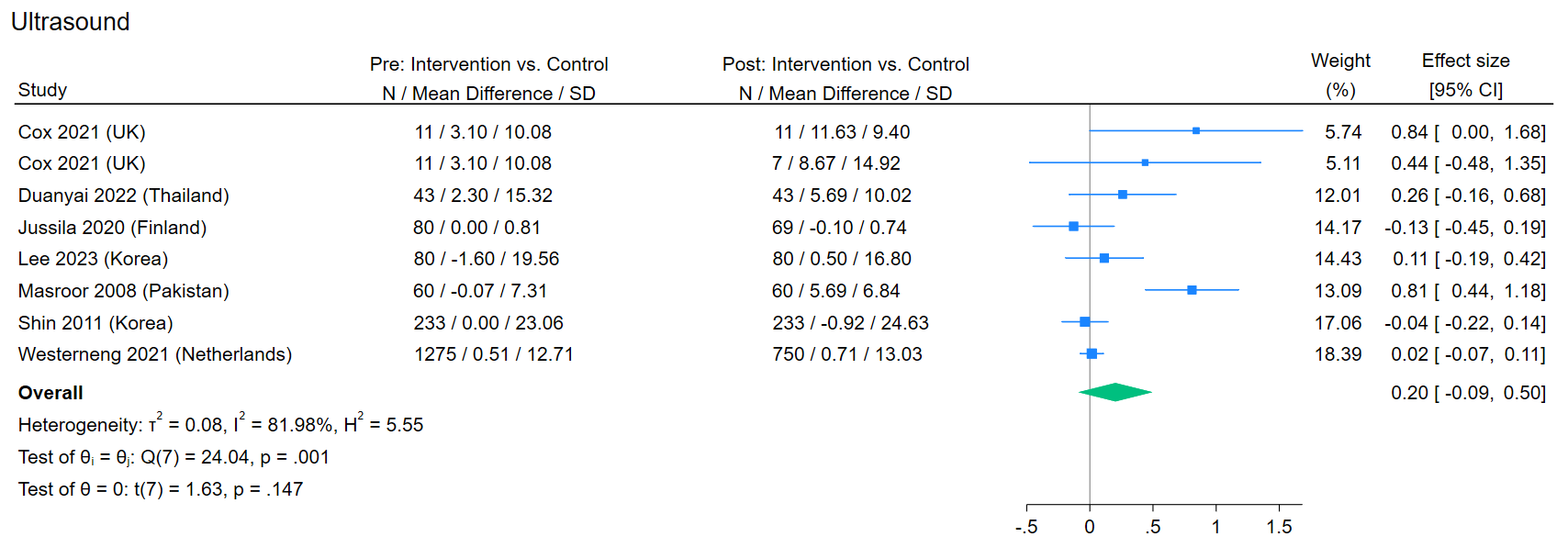


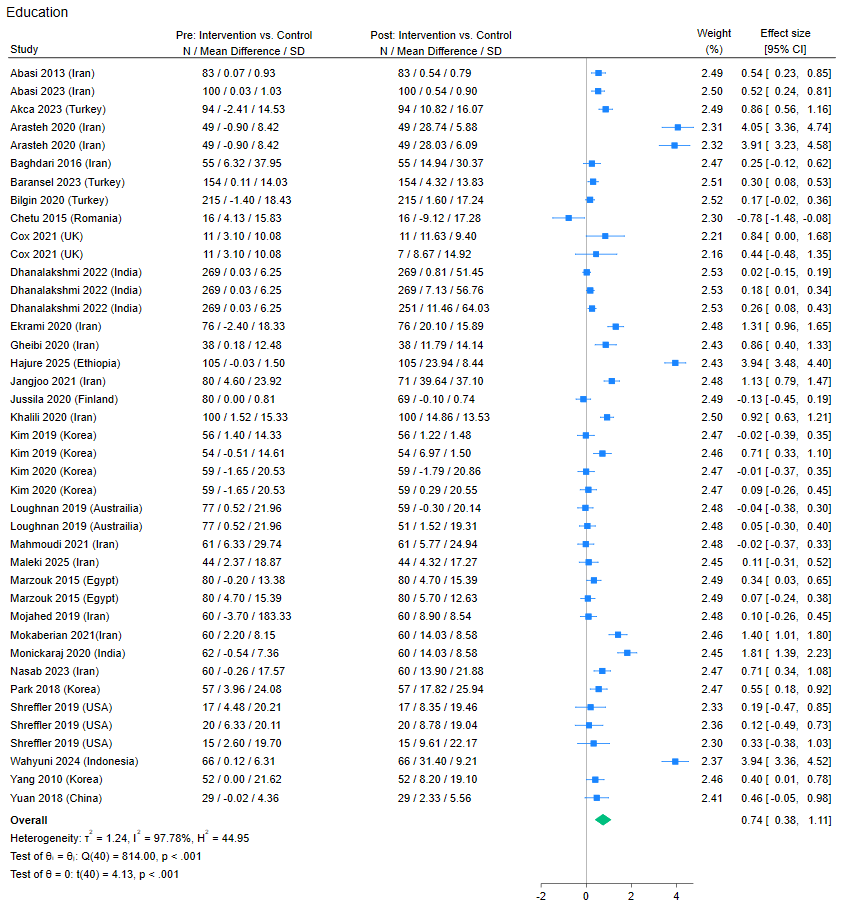

Supplement: Supplementary file 2 — Supplementary file2 (DOCX 746 KB) [file 737_2025_1630_MOESM2_ESM.docx]
