# Supplementary material for: Prenatal attachment interventions: a comprehensive systematic review and meta-analysis
Source: Arch Womens Ment Health. 2025 Nov 8;28(6):1447–71. doi: 10.1007/s00737-025-01630-w (PMC12702810; doi:10.1007/s00737-025-01630-w)
Supplement: Supplementary file 3 — Supplementary file3 (DOCX 95 KB) [file 737_2025_1630_MOESM3_ESM.docx]

**Supplemental Figure 3 Description of interventions**

**Abasi 2010 (Iran)** forms to record the number of fetal movements every morning after breakfast for one month.

**Abasi 2013 (Iran)** 4 sessions of 2 h were held weekly. They received education about MFA. In the first session, concepts such as attachment, MFA, benefits of attachment, and methods of performing attachment behavior were taught. These behaviors included counting fetus movements and recording them, positive imagination of fetus appearance, speaking to the fetus, imagining breastfeeding the baby, and touching the abdomen. Meanwhile, mothers were given forms to record these behaviors and were asked to complete them weekly. In the following sessions, how to practice these behaviors was discussed.

**Abasi 2023 (Iran)** The intervention group was divided into five groups ofm11 couples. Training materials were provided to people through WhatsApp and in the form of voice. For this purpose, the intervention group received four 30‑minute training voices through WhatsApp for 4 weeks, and it was recommended to perform a series of behaviors related to the content of each session to provide their feedback in the next meeting with the coordination of the group members, one hour per week was dedicated to discussing the training materials. The participants’ questions were also answered via text messages or phone calls. Textual behavioral recommendations were placed in the WhatsApp group for further emphasis. From the second session onwards, the mothers were asked about their spouses’ behaviors, and the feedback was reviewed.

**Ağapınar Sahin 2023 (Turkey)** The researchers conducted the laughter yoga practice with five experimental groups (the number of people in the group was 9, 8, 7, 9, and 10). They implemented laughter yoga in the experimental group after the participants received routine prenatal training at the pregnancy school. The researchers organized a 45-minute laughter session for the pregnant women in the experimental group and held eight 45-min sessions of laughter yoga in the experimental group twice a week for 4 weeks. In the laughter sessions, applied for 4 weeks, the pregnant women were provided with 30 minutes of deep breathing exercises and milk shake, shoulders anti-stress technique, and laughter exercises such as laughter with mobile phones, laughing even when angry, and crying and laughing, and 15 minutes of laughter meditation and relaxation. In the laughter yoga session specially adapted to pregnant women, the researchers used the shoulder anti-stress technique, and since crying and laughing have the same frequency, they combined these two exercises when using them in pregnant women. The researchers arranged the end of the session specifically for pregnant women.

**Akarsu 2018 (Turkey)** Each yoga class consisted of approximately 5-6 pregnant women. The yoga intervention was designed by a certified pregnancy yoga instructor. Each yoga intervention was held for approximately 4—45 min with yoga asana sessions lasting 30 min. A mixture of standing and seating positions were introduced and practiced. Approximately 9-10 position were repeated each week. Repetitions were consistent from week to week and linked one position to the other. Each yoga intervention ended with a 15 min deep relaxation period with the subject lying supine. Progressive relaxation and meditation techniques were introduced during this time. Yoga practice steps. -Breathing awareness study for 5 min. -Practice for twenty minutes -Mother communication and meditation for 5 min. One of the researchers held the yoga practice every day of the week. And pregnants were expected to participate in yoga practice twice a week on a regular basis for 8 weeks.

**Akbarzade 2014 (Iran)** fathers were trained regarding the attachment skills through four 60-90-minute sessions held once a week. The contents of the sessions were as follows: first session: parental attachment to the baby and attachment behaviors, second session: concepts of maternal-fetal and paternal-fetal attachment and their effects on the parents’ physical and mental health and fetal growth, parents’ anxiety during pregnancy and its reasons and impacts, and father’s role in reduction of anxiety, third session: how attachment is created, beginning of attachment and its signs, father’s role, and acceptance of father’s role, and fourth session: father’s role in attachment, effect of focusing on the fetus, considering the fetus as an independent being, getting familiar with the sensory abilities of the fetus, and father’s role in breastfeeding after birth. After the interventions, the fathers were followed up through telephone contacts and were asked to transfer their information to their wives. A reminder session was also held at the 38th week of gestation.

**Akca 2023 (Turkey)** Mindfulness-Based Stress Reduction (MBSR) program is designed in 8-week sessions and is one of the most well-known mindfulness interventions. The MBSR program includes body scans, gentle stretching, and yoga mindfulness exercises, as well as a wide range of holistic techniques and therapies to apply mindfulness to daily life experiences, including coping with stress. Two sessions per week for 1 month and home assignments given throughout the program. In all sessions, mindfulness exercises such as 3-min breathing, body and breathing exercises, body scanning meditation, sitting meditation, mountain meditation, and compassion meditation were applied to the participants. It was done in sessions and individual applications in an environment where pregnant women were not disturbed at any time of the day. The sessions of the MBSR program were based on the meditation techniques of Mindfulness Therapy. The live online MBSR program was provided via mobile communication tools. Sessions were conducted as individual or group sessions according to choice of high-risk pregnant women by determining the days and time when they were available online. Each session lasted an average of 40–60 min.

**Alhusen 2020 (USA)** Intervention participants received the 6-session version of the MB course, consisting of six 2-hour intervention sessions delivered weekly in a group format, with ten participants per group. The MB Course is a manualized cognitive-behavioral treatment (CBT) intervention, developed on the theoretical perspectives derived from attachment theory, developed to reduce the risk for perinatal depression among low-income, ethnically diverse women.

**Amiri 2023 (Iran)** The intervention program started in the 22nd-24^th^ weeks of pregnancy and was held during eight face-to-face counseling sessions including the structural dimensions of CBC, and one 90-minute session per week. Counseling program and process during sessions Overview of each session content. First Pretest introduction, presentation of plans, counseling, assignments, and introducing detailed and general goals. 1) Familiarity of members with each other 2) Establishing a positive relationship between group Members 3) Creating a sense of mutual trust and motivation to continue the sessions 4) Explanation of mental health Second A summary of the fetal development process and the evolution course until birth, questions, and answers, creating empathy, group activity, drawing of the fetus, homework. 1) Self-awareness as a strategy to increase mental health. A summary of the fetal development process and the evolution course until birth. Creating empathy. Drawing of the fetus aiming at creating interest and curiosity toward the fetus. Third Examining homework, explaining MFA (the nature of attachment, the importance of attachment, its effect on the postnatal mother-newborn relationship, and the formation of safe and healthy attachment), consulting techniques for increasing MFA and its implementation in the session, touching and recording the number of movements, recognizing the fetal position at home, and recording in a designed chart, homework. 1) Introducing the fear of childbirth. 2) Identifying mental errors and ways to reduce them. 3) Explanation and description of MFA (the nature of attachment, the importance of attachment, its effect on the postnatal mother/newborn relationship, and the formation of safe and healthy attachment. 4) Techniques to increase MFA. Fourth Examining homework, explaining, and examining the expectations, expectations of mothers' physical/emotional changes during pregnancy and after, training to maintain affective/emotional and physical health of mothers and fathers during and after pregnancy, homework. 1) Explaining mothers' physical changes during pregnancy. 2) Explaining mothers' emotional changes during pregnancy. 3) Teaching to maintain mothers' and fathers' emotional and physical health during and after pregnancy. Fifth Examining homework, group discussion about mothers' expectations of their husbands' role in pregnancy, performing techniques to increase MFA, training correct breathing. Group discussion about mothers' expectations of their husbands' role in pregnancy. Performing techniques to increase MFA. Sixth Examining homework, explaining tension, discussing, and counseling on ways to cope with tension, such as relaxation, deep breathing, mental imagery, counseling, and doing relaxation in class, during and after pregnancy. Description of tension. Discussing and counseling on ways to cope with stress during and after pregnancy. Teaching relaxation with mental imagery. Training deep breathing. Seventh Examining homework, counseling about the maternal role and role-playing, doing techniques to increase MFA, couple's attendance at the session, and counseling on parenting techniques. Counseling about the maternal role and role-playing. Performing techniques to increase mother-fetus attachments. Couple's attendance at the session and counseling on parenting techniques. Eighth Counseling on the importance of the paternal role during pregnancy, couple's presence, explaining depression and the coping ways during pregnancy, counseling on problem-solving skills, performing techniques to increase MFA, completing the questionnaire. Explanation of depression and the coping ways during pregnancy, counseling on problem-solving skills. Performing techniques to increase MFA. Sample assignments: drawing of the fetus, homework (As homework, mothers are asked to touch their stomachs, pay attention to the fetal movements, and write them down in a notebook). Education techniques to increase MFA and Performing techniques to increase MFA. Teaching to maintain mothers' and fathers' emotional and physical health during and after pregnancy. Teaching relaxation with mental imagery, training deep breathing.

**Arasteh 2020 (Iran)** The intervention group received eight weekly group cognitive therapy sessions. Each group consists of nine participants and each session lasts 60-90 minutes. The structure of intervention was developed based on psychological adaption to pregnancy model, and cognitive therapy. Session 1 Introducing participants to each other Psychoeducation on cognitive behavior therapy model Education about relationship between situation, negative automatic thoughts, emotions and physical reactions, and behaviors. Providing Thought Record Worksheet (TRW) Homework assignment: filling out TRW Review of the session. Session 2 Homework review. Education about relationship between situation, negative automatic thoughts, emotions and physical reactions, and behaviors focusing on pregnancy. Education on understanding and identification of negative thoughts /images about pregnancy

Homework assignment: filling out TRW about physical and emotional changes related to pregnancy. Session 3

Homework review. Psychoeducation on evaluation of thoughts and cognitive restructuring techniques (e.g., Evidence gathering, and cost benefit analysis) to form an alternative rationale thought. Homework assignment: filling out TRW and gathering evidence about their ability to accept the mother role. Session 4 Homework review. Psychoeducation on identifying thought error. Relaxation training. Talking about adaptation to pregnancy through empathy of the woman with her own mother. Homework assignment: filling out TRW focusing on thought errors, relationship with the husband, as well as relaxation exercise. Session 5 Homework review. Exercise on cognitive restructuring techniques and developing alternative rational thought. Talking about empathy and collaboration of spouse, reliability of the spouse, intimacy, interpersonal problem solving, and husband collaboration in caring for the baby. Homework assignment: filling out TRW focusing on labor and childbirth, and finding alternative rational thought, relationship with the husband, as well as relaxation exercise. Session 6 Homework review. Psychoeducation about differences between rationale thought and positive thought. Education about procedure of labor and childbirth.

Homework assignment: filling out TRW focusing on fear of pain during labor, inability, and loss of control during childbirth, as well as relaxation exercise. Session 7 Homework review. Talking about experimentation of new rational alternative thought. Education on designing of behavioral experiment. Education on cognitive and behavioral pain management techniques. Homework assignment: filling out TRW focusing on fear of pain during labor, inability, and loss of control during childbirth, as well as relaxation exercise and pain management techniques.

Session 8 Homework review. Talking about behavioral experimentations. Preparation for labor and childbirth. Trust in the medical staff, management of negative emotions during childbirth.

**Arioli 2025 (Italy)** Women were instructed to engage in the intervention activities starting from the 29th week of gestation, with a recommended frequency of at least 4 times per week, each lasting at least 15 min. They were provided with written instructions and a diary to record their activities and experiences. Along with background information on foetal development and research references, the diary contained a series of questions to be completed each week. These questions pertained to the frequency of intervention sessions carried out in the week, the specific musical tracks selected during each session, the level of engagement with the prescribed activities, and the emotional responses experienced during the sessions. The Passive Protocol consisted of listening to tracks to be selected from a playlist of relaxing sounds. The active protocol involved performing activities designed to provide multisensory stimulation to the foetus. Participants were requested to listen to songs (approximately 75–85 decibel Sound Pressure Level), hum along with the melodies, rhythmically tap their abdomen and gently bounce around in sync with the rhythm of the music.

**Asari 2021 (India)** participants of experimental group were guided and asked to record the FMC experienced by them for seven consecutive days, twice a day for 20 minutes.

**Astuti 2021 (Indonesia)** The manual for Prenatal Gentle Yoga trainers specifies that the yoga should be performed during 90-minute classes which consist of: centering (5 minutes); pranayama/breathing (5 minutes); warming up

(5 minutes); 60 minutes of exercises that include the Prenatal Sun-Salutation A/B, Soft Form Prenatal Gentle Yoga A/B, and asanas (standing, kneeling, and sitting poses); supine/restorative poses (5 minutes), finishing with 10 minutes of relaxation and affirmations. In this study, respondents in the yoga group attended Prenatal Gentle Yoga for a minimum of six sessions of 90 minutes each, once weekly.

**Ayala 2025 (USA)** Fetal movement was acquired using a Toitu MT-516 fetal actocardiograph. This device employs a single wide array transabdominal Doppler transducer to detect fetal movement, fetal heart rate, and uterine wall activity. Prior to fetal monitoring, participants were asked not to eat or drink within 1.5 h of the study. Participants sat in a quiet, dimly lit room in a semi-recumbent position with the Toitu MT-516 transducer attached to their abdomen. On average, there was a 30 min recording period across all participants.

**Azogh 2018 (Iran)** the intervention group received 4 sessions of cognitive behavioral training for 4 weeks.

**Badar 2023 (Egypt)** The pregnant women were divided into 10 groups and each group included 6 women. Each group was received three sessions; the duration of each session was 30-45 minutes including periods of discussion according to pregnant women's response. At the beginning the researcher gave the pregnant women the teaching booklet and introduced an orientation about the program including the general objectives. The researcher provided the pregnant women general knowledge about meaning of fetal movement, factors that increase fetal movement, the time of first sensation of fetal monitoring for primigravid's, the number of fetal movements felt during hour, the most appropriate position to count fetal movement, time of consulting obstetrician regarding fetal movement, the best time to measure fetal movement during the day, factors that preventing sensation of fetal movement, the pathological causes of absent sensation of fetal movement, the investigations of the fetal wellbeing, the actions that the pregnant women should do in case of decreasing fetal movement , the instructions that the pregnant women should know about fetal movement and the actions that the pregnant women should do when feeling the increase of fetal movement and the foods that increase the movement of the fetus . The second session: implied on the implementation of the practical part of the educational program and involved training the pregnant women's on counting the fetal movements using Cardiff method and record the fetal movement on fetal movement counting chart. The researcher used different teaching methods as demonstration and re demonstration. The pregnant woman should choose the time when the fetus is active and relax to feel the movements of the fetus, the pregnant woman should be lie on the left side, with the head comfortably supported by a pillow and that help to feel the movement more strongly. Placing the hand on the abdomen to feel the movement of the fetus while counting the movements, and the fetus is more active after eating meals or drinking a drink containing sugar, or after exercising. The pregnant woman should get a notebook or set up a schedule to start by writing the number of movements starting with the first movement and writing the time when the movement started. Upon reaching the ten movement, the time must be written and compared with the time of the first movement, and that helps the pregnant woman to know the time it took for the fetus to reach 10 movements. Before start calculating the movement of the fetus, write down the week of pregnancy as well as the time when the kicks began. Note how long it took the pregnant woman to reach ten movements. The fetus should move at least ten times in an hour. The third session: Each pregnant women was trained about prenatal attachment including differentiation from fetus, interaction with the fetus, attribution of characteristics of the fetus, give of self to the fetus such as maintaining a nutritional, adequate rest and sleep, regular exercise and regular prenatal car, fetal parts palpation, speaking to the fetus.

**Badem 2022 (Turkey)** In the first stage, the participants were given in-person training for counting the fetal movements and tracking the position of the fetus. The training was completed in 30–45 min. The training content included topics such as setting a comfortable environment and a suitable position for the pregnant women, how to count and how to evaluate the movements (kicking, turning, hiccups, strong and weak movements, etc.). Additionally, the women were taught to determine the position of the fetus and the Leopold maneuvers 1 and 2. The women were given a training brochure and a form to record the count of the fetal movements and the position of the fetus. In the second stage, the participants were counselled twice a week for 4 weeks by phone. Each phone call lasted between 5–10 minutes. In the phone call, the participants were asked whether they followed up regularly. They were asked to follow and record the movement and position of the fetus. They were asked to do this daily, at least once a day, for 15–20 min, at any time of the day when the fetus was active, and their questions were answered. They were able to contact the researcher via phone whenever as per their requirement.

**Baghdari 2016 (Iran)** Eight one hour weekly routine sessions of prenatal educational classes. In addition to the routine classes, the intervention group participated in four 60-minute sessions on adaptation to pregnancy. the subjects in the experimental group were given an educational booklet and a CD concerning the outline of the educational classes. The researcher called the mothers weekly to remind them to study the booklet and watch the

CD. 1 -Greeting, introducing the sessions’ facilitator and the students to each other; -anatomy and physiology of female reproductive system, fertilization, fetal growth, and development stages. 2 -Encouraging the women to discuss their husbands’ and families’ feelings about their pregnancy, pregnancy exercises, body image, physical and mental relaxation techniques; -question and answer on the participants and their families’ problems regarding the pregnancy. 3 -Group discussion on pregnancy adaptation problems; -question and answer and counseling facilitated by a psychologist. 4 - Physical and psychological changes due to pregnancy and the ways to adapt to them; -group discussion about mothers’ concerns regarding fetal health; -techniques of creating relationships with the husband and the family, and the strategies to deal with interpersonal problems.

**Baltaci 2022 (Turkey)** The IG listened to lullabies, and accompanied by lullabies touched their abdomen and thought about their babies. The lullaby recital consisted of 10 different lullabies with a mean duration of 2 min, for a total of 20 min. The lullabies preselected by the investigator were among the best-known ones from the Turkish culture: “His/her mother loves him/her too much,” “blue cradle,” “green apple,” “let me toss and catch you,” “may you have hives of honey,” “may he/she sleep and grow,” “boatman,” “dandini dandini dastana,” “sleep my little one, it is morning again,” and “ninnanna,” which belong to a CD named “Our Lullabies”. The content of these traditional lullabies is generally mother-baby love, sleeping-growing, good wishes, and good feelings. To provide a homogeneous intervention and to avoid bias, the personal lullaby preferences of the pregnant

women were ignored. The IG listened to the lullabies for 20 min per day, for 2 successive days using an MP3 player.

A duration of 20 min was determined, consistent with the research results and routine procedures in perinatology clinics. These lullabies, by its nature, had a slow rhythm (the beat varied from 60 to 70 beats per minute, measured by a metronome), in harmony with normal heart rate. The participants were instructed in the operation of the MP3 player. They could adjust the volume of the music. Ambient sounds were too low to be heard with earphones.

While they were listening to the lullaby, they were told to think about their babies, and feel them by touching the abdomen. This intervention was applied in single rooms during bed rest, when the participants were available and felt ready. The door and windows of the room were closed, and the participants were left alone to respect their privacy. They were ensured to have an empty bladder and turned off their cell phone and did not receive medical treatment during the intervention.

**Baltaci 2023 (Turkey)** The study included two intervention groups. The participants in the LG were instructed to only listen to the recording of lullabies selected by the researcher for 30 minutes every day for two weeks, at home. The participants in the MG were given recordings consisting of nature sounds, Western classical music, Turkish classical music, and lullabies that were arranged by the researcher and were instructed to select and listen to any of these at home for 30 minutes every day for two weeks. They were asked to listen to the same music every day and not change the selection. The participants in the music intervention groups were informed about the form and conditions of listening to the given music records at their homes. The music files were sent to the participants via mobile communication applications (e.g., Telegram, WhatsApp), so that they could listen to them on their own phones; they were also asked to download/save these files. The record of lullabies selected by the researcher consisted of 12 different lullabies, each of which was 2.5 minutes long on average, constituting approximately 30 minutes in total. The nature sounds record, consisting of water, wave, rain, wind, dolphin, bird, and forest sounds, and the Western classical music record, consisting of some of the most popular works of Beethoven, Vivaldi, Mozart, Chopin, and Bach. lullabies compatible with a normal heart rate (varying in the range of 60-70 bpm as

measured with a metronome) and with a soft melody as well as other pieces mentioned above were used. The participants were instructed to use their personal headphones in a quiet and dimly lit room to prevent the effects of ambient sounds while listening to the music, adjust the volume of the music to a level that they preferred and was appropriate, empty their bladder, and not be hungry. They were instructed to listen to the music records at a convenient time during the day, in a semi-prone position that would allow them to be comfortable, alone, and without falling asleep. To prevent the interruption of their music-listening process, they were told to download

the music records to their own phones, listen to these records on their phones, and activate the flight mode on their phones to avoid any intervening calls.

**Baransel 2023 (Turkey)** The ABIP includes (1) perceiving/counting fetal movements; (2) music therapy; (3) preparation for the baby; (4) writing notes/letters to the baby; and (5) watching images of the fetus/pregnancy. The program was completed in a total of 5 days, with one intervention per day as follows: solely sensing/counting fetal movements on day 1, music therapy and sensing/ counting fetal movements on day 2, preparation for the

baby and sensing/counting fetal movements on day 3, writing notes/letters to the baby and sensing/counting fetal movements on day 4, and watching images of the fetus/pregnancy and sensing/counting fetal movements on day 5. An ABIP kit, which contained the materials to be used by pregnant women during ABIP interventions, was provided to them by the researchers at the first meeting. ABIP intervention materials were included in the ABIP kit.

**Bellieni 2007 (Italy)** Prenatal education tries to ensure mother-fetus interaction through prenatal bonding and to cope with the mother’s psychological needs. It is based on favoring maternal-fetal bonding through participation in a series of lessons that develop an awareness of fetal development and response to parental stimulation. Women attended prenatal education courses (PEC). The PEC were composed of a series of five 1-hour lessons the one group attended during the second trimester of pregnancy. The syllabus included the basics of fetal physiology and development, singing sessions, dance sessions, and massage-through-the -womb sessions. The lessons were based on Veldman’s studies on haptonomy. Their main aim is to raise awareness of fetal presence and development and to interact with the fetus by stimulating it gently and perceiving its responses.

**Bhandari 2025 (India)** The interventional group underwent fifteen‑minute single sessions based on performing a daily fetal movement count. After this, they were provided with the daily fetal movement count chart to maintain the fetal movements perceived for the next two weeks.

**Bilgin 2020 (Turkey)** Education was conducted once a week for five weeks’ duration. Each course lasted 15 hours and was done with a maximum of 10-12 participants. Visual tools, models and video demonstrations were utilized in the education. Presentations and interactive education methods were also used. Breathing and relaxation exercises were conducted at the end of each 30-45 minutes session. Baby and birth-related affirmations were given to the participants. They were asked to repeat these exercises and affirmations at home daily. 1st week: Physiology of delivery, indications of birth, fear of birth, relaxation techniques, 2nd week: Stages of delivery, effects of hormones at birth, emotional and physical support, the importance of communicating with the baby on the mother's womb, the effects of relaxation on maternal and fetal. 3rd week: Non-drug relaxing techniques to cope with birth pain. 4^th^ week: Anesthesia at birth, interventions for delivery, making a birth plan. 5th week: Mother-infant relationship, baby care, breastfeeding.

**Briscoe 2022 (UK)** The Building Attachment and Bonds Service (BABS) is a specialist parent infant mental health service (PIMHS) which offers psychotherapeutic, parent-infant interventions which include: parent infant psychotherapy (PIP – Ghosts in the Nursery), video interaction guidance (VIG), systemic/family therapy interventions, mindfulness-based interventions, and attachment-based therapies.

**Carvalho 2025 (Portugal)** Participants were sequentially allocated into three groups: 1) control group (CG), without any participation in prenatal activities using singing or humming; 2) prenatal music therapy group (MTG); and 3) prenatal vocal training group (VTG) with prenatal singing sessions. Participants of MTG and of VTG were engaged in five weekly sessions of approximately 75 min. In the music therapy group, pregnant women were led by two music therapists who engaged participants using improvised humming, singing (lullabies and sentimental songs about motherhood), and songwriting. In addition, there were two individual online sessions to create a song for the unborn infant. In the vocal training group, pregnant women were guided by two singing teachers who led vocal exercises using a flowball device to enhance low-frequency components. In addition to group sessions, tutorial videos were added to provide individual support for participants' daily vocal training. Aim was to practice a daily vocal routine of breathing and semi-occluded vocal tract exercises tailored to enhance phonatory function towards the increase of low-frequency spectral components and flow phonation. These exercises included singing lullabies, phonatory breath management, intending to increase subglottal voice level and chest wall vibrations, and enhancing intrauterine sound transmission.

**Celik 2019 (Turkey)** Information about embryonal-fetal developmental stages was provided to the group. The pregnant women were given brochures indicating the use of the first and second Leopold's maneuvers. The

first and second Leopold's maneuvers were demonstrated and then the women were asked to try these maneuvers

on themselves and listen to the fetal heartbeat. during the 32nd and 36th week of pregnancy, abdominal examination was performed using Leopold's maneuvers and fetal heart sounds were heard using a manual Doppler.

**Chang 2004 (Korea)** The treatment of Taegyo-focused prenatal education was scheduled for 8 hours (2 hours a week for 4 weeks) and used lectures, demonstrations, practice, training, discussion, and sharing of experiences. The classes were held in health education rooms in the public health center. The Taegyo-focused prenatal class provided basic information like other prenatal classes plus the unique content of Taegyo (Table 1). Participants were

provided brochures containing a checklist, which listed items of Taegyo practices and so they were able to keep

records everyday throughout the participation. The practice of Taegyo promotes a variety of caring activities. These include control of the mind to be peaceful and joyful, maintenance of a graceful demeanor; selection of foods by the quality of the ingredients, recipe, and shape, use of discreet judgement regarding objects, pursuit of aesthetic activities to refine the emotions, identification with saints and great persons, and practice in control of, and protection from, sensory and sexual stimulation. Min 1 2 3 4 10 Breathing, Relaxation, B. R. & MPE B. R. & MPE B. R. & MPE & Maternal Physical 10 Exercise (B. R. & MPE) 10 MPE 10 Writing & Sharing: Sharing: Lecture:

Motivation & purpose Perceived Ideas about Understanding of the pregnancy childbirth 10 Watching a childbirth & caring for video the newborn 10 Lecture: Understanding 10 Milk Break childbirth 10 Lecture: Importance Milk Break of the internal environment of the fetus 10 Writing & Sharing: Milk Break A prayer letter to The newborn child 10 SMFR Milk Break 10 Hospital tour Taegyo (Delivery Rm., Meditation II Maternity unit, & Nursery) 10 Demonstration & Practice: Taegyo Declaration of love Strengthening the maternal Meditation I by the mother-fetal relationship (SMFR)

**Chang 2015 (Taiwan)** Participants in the experimental group received a prerecorded music compact disc (CD) to listen in addition to their routine prenatal care. Five types of prerecorded music compact discs (CD) were created for this study. Each CD contained approximately 30min of music, respectively, in five categories: crystal music, nature sounds, classical music, lullabies, and symphonic music. The tempo of the music selections was selected to mimic the human heart rate (60—80beats/min). Participants in the experimental group were given the prerecorded CD and asked to listen to the music at least 30min a day for 2 weeks, while they were at rest or at bedtime and on a self-regulated basis, that they would feel more relaxed. The participants listened to the music in their preferred category and were permitted to listen to the music either over speakers or through earphones.

**Chetu 2015 (Romania)** Conducted for 6 weeks. structured the attachment intervention for the pregnant women from the experimental group in 4 sessions. Each session starts with a check-up of the changes having occurred from one session to another in their relationship to their fetus and a brief introduction of that session, to carry on with a provocative exercise, specific to experiential psychotherapy, based on art-therapeutic techniques. At the end of each session, the participants are encouraged to speak about their here and now experiences to facilitate the identification of behavior patterns during pregnancy, their causes, and effects. Art materials used: Various items are placed on a table – shells, beans, crosses, colored feathers, little stars, coins, toys, rope fragments, dry lavender bags. Instructions: The participants are invited to look at the table filled with objects, explore them at their pace – look at them, handle them, touch them, etc. and to be aware which of them draw their attention particularly, which of them they spend more time exploring and which of them they look at without showing them any special interest. After these warmup exploration moments, they choose and put on a board at least 3 items to make a symbolic representation of their own relationship to their fetus. Art materials used: A3 sheets of paper, crayons, watercolors, brushes Instructions: Following a relaxation and guided imagery exercise, each pregnant woman expresses by drawing the shape of her body, that of her fetus in her body, and the areas of her body in which she feels its presence. The silhouette does not need to be realistic, nor anatomically correct. It is important that the image is representative for her as she is here and now. Session no. 3 – Title: Expressive connections Art materials used: Plasticine Instructions: We start by a guided imagery exercise performed while the expecting mothers use a piece of plasticine. During the exercise, the time is rewound to the time before they got pregnant, for them to recall images, thoughts, and emotions in the professional, family environment, the couple relationship, and the current context. Starting from this exercise, we ask the participants to give their fetus a symbolic shape. Session no. 4 – Title: Emotional Mandala Art materials: pictures and written words, crayons, watercolors, and brushes. Instructions: We start by a guided imagery exercise oriented towards the emotions and fears they experience throughout their pregnancy period, then the expecting mothers express all these through a collage mandala made using the art materials provided to them.

**Coté 2020 (USA)** An ultrasonographer performed a 20-minute 3D/4D ultrasound examination. The ultrasonographer

had neutral interactions with the participants and specifically targeted the fetus’s face. All participants watched the screening in real time on a large-screen television. Neither group was permitted to take pictures, nor did they receive

any thermal printed images. After participants in the ultrasonography plus 3D print group left the clinic, we extracted the digital image and communications in medicine data from the 3D ultrasound machine and segmented, cleaned, and converted the data to a stereolithography (STL) file. The image was printed by a 3D printer with biodegradable plastic, polylactic acid.

**Coté 2023 (USA)** Ultrasonographer performed a 20-minute 3D/4D ultrasonography examination that specifically

targeted the fetus’s face. We emphasized neutral interactions and limited discussion between the ultrasonographer and the participants. We did not allow recordings, and participants did not receive any images at the time. After the ultrasonography examination, participants left the clinic knowing they would need to return in

1 week to receive an image of the fetus. One week after the initial ultrasound examination,

participants returned to the clinic and received a small shadow box that contained the 3D-printed model of the fetus or a foldable card that contained a 3D printed picture of the fetus. For the 3D-printed model group, we extracted deidentified data from the ultrasonography machine, which we segmented and converted into a stereolithography file. The image was printed by a 3D printer with a biodegradable plastic. For the 3D printed picture group, we

produced a standard 3D printed image from the ultrasonography machine.

**Côté-Arsenault 2014 (USA)** The intervention goal was to provide a safe, supportive environment, reduce anxiety and depression through normalizing the PAL experience, promote prenatal attachment, and teach skills known to reduce stress and anxiety. This entailed getting to know each woman, her pregnancy and her loss story, focusing on being with her and where she was emotionally in her current pregnancy (including prenatal attachment to her baby), offering anxiety-reducing coping skills, encouraging use of her pregnancy diary, providing information on topics of interest or concern, and continually maintaining belief in her ability to focus on positive events, reduce anxiety, and make it through the pregnancy. The intervention consisted of HV, pregnancy diary, and anxiety-reducing skills teaching. The content of the HV, based on evidence of women PAL, included commonly experienced emotions, physical milestones, and social aspects of pregnancy as they change across pregnancy. Conversation included all the women's children living, deceased, and the unborn. Contact with the APN between HV was encouraged. each woman was provided a Guided Pregnancy Diary, developed by the PI for this study, to record pregnancy events and personal entries, as well as to reinforce the content from HV and skill practice. Four skills, expected to affect anxiety in pregnancy, were taught: relaxation, problem solving, daily fetal movement records, and “I” message training. Women were assessed for their current skill level and their need for skills. Anxiety-reducing skills were demonstrated by the APN, with immediate return demonstrated by the mother. Skill practice and utilization were noted in the pregnancy diary by the mother. Skills were reinforced in subsequent HV as needed.

**Cox 2021 (UK)** The 60 min ‘Baby CHAT’ intervention comprised psychoeducational material. 1. Welcome 10 Introductions ●Overview of Baby CHAT ● Confidentiality ● Participants share something they are looking forward to about meeting their baby. ● Baby CHAT booklet given 2. Social and unique baby 15 ● Presentation of information about social development of a baby post-birth. ● Video of a 6-week-old baby interacting with her parents (displaying early baby ‘chat’) ● Video material showing reciprocal parent–baby interactions highlighted and

group discussion encouraged. ● Parents asked to consider when baby’s social development occurs, for example,

before or after birth. ● Presented with information about fetal development during pregnancy. 3. Four-Dimensional

scan research 15 ● Video clip ‘Your unborn baby and you’ containing 4Dultrasound images of fetuses mouthing sounds presented outside the womb. ● Discussion encouraged, to help parents think about social development of

babies from 32-weeks. ● This could enable parents to consider unborn babies as individuals, with their own experiences to encourage reflective thinking. ● Think about baby getting ready for when they meet them. 4. Getting to know your baby 5 ● Parents were asked to think about and visualize characteristics of their own baby for example, routine in the womb, likes and dislikes, bump name. ● What have you learnt about your baby so far?

● What do you think their personality and temperament is like? ● What do you think baby is doing in the womb?

● This was to encourage RF by asking parents to think about the experience of their baby (RF) and to further develop feelings of connection (bonding). 5. Baby CHAT (doing things together) 10 ● Discussion around activities parents can do with baby before birth, for example: ● singing ● reading ● talking ● playing music ● mindfulness

● Thinking about good times to do these activities for example, choosing more upbeat music if baby is awake and moving around, more sedate activities when baby is less active. ● Aim to encourage parents to think about developing a stronger connection with baby (bonding) and about their baby’s experience when choosing activities

(RF). ● Plan when/where/how parents will have a go at one or more of these activities. 6. Ending 5 ● Ask everyone to write something positive they will take away from the session about their baby’s development. ● Aim to reinforce key messages and leave parents with positive feelings at the end of the group.

**de Jong-Pleij 2013 (Netherlands)** In both groups, a basic US examination was performed including assessment of the amniotic fluid volume, placental location, fetal position, biometry, and an anatomical survey. Approximately 5min was spent looking at the face when it could be best visualized during the examination. Three-dimensional volumes were collected starting from the mid-sagittal, coronal, and axial planes, with display of the multiplanar mode. Finally, the render mode was activated, and moving surface rendered images of the face were shown to the mother. In group 2, an equal time was spent showing images of the face, aiming at obtaining clear mid-sagittal, coronal, and axial views of the face. In both groups, the maximum examination time was 30min. All ultrasonographers were instructed to create a friendly and reassuring atmosphere with positive and friendly verbal feedback. Special attention was paid to explain the specific age-related appearances of the fetuses and effects of artefacts.

**Delaram 2018 (Iran)** An information brochure was given to the women who were in the intervention group, and in this brochure, they were trained how to count the fetal movements daily at morning from 28 weeks of gestation to 37 and record them on their chart. The intervention group began to count the fetal movements daily from 28 to 37 weeks of gestation. To ensure the fetal movement counting in the intervention group, one person of the research team telephoned the women every two weeks.

**Dhanalakshmi 2022 (India)** Steps/weeks of gestation Interventions Modality Strategy used Step I (24th week) Antenatal exercises Video-assisted teaching cum demonstration Visual, auditory, kinesthetic strategy Step II (32ndweek) Kick chart monitoring. Lecture cum demonstration. Visual, auditory, read, kinesthetic strategy.

Preggy imitator. Lecture, demonstration, and simulated practice. Visual, auditory, kinesthetic strategy. Step III (33–34 weeks) Reinforcement of VARK strategies. three interventions (antenatal exercises, kick chart monitoring, and use of a preggy imitator) along with routine education to the experimental group.

**Duanyai 2022 (Thailand)** In the interactive feedback group, the participants viewed the ultrasound monitor and were given a running description of fetal anatomy by the sonographer.

**Ekrami 2020 (Iran)** Six group counselling sessions (the first session for introduction and acquaintance for 30 minutes and the remaining five sessions for 90 minutes) were planned and held by the researcher for the intervention group in groups of 7 to 10 at one-week intervals for six consecutive weeks. An individual counselling session was also held for each participant of the intervention group, upon the request of some of whom the number of individual

counselling sessions was increased to 3. Counselling principles and techniques were observed and utilized in all sessions to establish an effective communication and the sessions were held in a respectful, intimate and confidence-building environment to facilitate individual participation in group discussions. The counseling sessions centered

around such topics as expressing physiologic, anatomic and hormonal changes during pregnancy, understanding fetal development during various stages of pregnancy, the complications of unplanned pregnancy on the mother and fetus and continued postpartum complications, the way maternal-fetal attachment is established, the significance of maternal-fetal attachment during pregnancy and strategies for greater adaptation during pregnancy, the effect of nutrition and prenatal care on maternal and fetal health, and pregnancy risk signs and symptoms and the ways of addressing them. Also, attachment skills and behaviors such as counting fetus movement, speaking to the fetus, fetal parts palpation and positive imagination of fetus appearance were taught.

**Estevao 2025 (UK)** The intervention was delivered biweekly in a yoga studio for 8 weeks. The classes were adapted to pregnancy and for all ability levels; lessons included loosening exercises (warm-up exercises), simple postures/asanas (standing, balancing, sitting, kneeling, supine, and restorative poses), and breathwork/pranayama exercises, meditation, and yogic counseling, as per the yoga module designed.

**Fiskin 2018 (Turkey)** Pregnant women in the experimental group were informed about how to perform diaphragmatic breathing exercises by researchers. After ensuring that they had learned the technique, the pregnant women were asked to perform this for 5 min every morning before they got out of the bed. The researchers distributed a brochure demonstrating the breathing exercises to pregnant women. The researchers wanted intervention group to note how long they performed the exercises and were invited to submit their notes and to fill their questionnaires twice a month. Firstly, pregnant women were asked to rest for 5 min in the supine position and lay on their back on a flat surface with pillows under their head and knees for support. One hand was placed on the abdomen and the other on the upper part of the chest wall. When inhaling, the hand on the abdomen moved upward, while the other hand remained as still as possible. When exhaling, the hand on the abdomen moved downwards,

while the other hand remained as still as possible again. Breathing was performed quickly, deeply and without causing tiredness. The breaths should be inhaled through the nose and exhaled through the mouth. Pregnant women were told to slowly let all the air out using controlled expiration. Precaution was taken to avoid hyperventilation.

During the training the researcher constantly emphasized to the pregnant women to relax and concentrate on the breathing. After the breathing exercise pregnant women were asked to rest for 5 min. After the technique had been learned, the exercises could also be performed while sitting.

**Gheibi 2020 (Iran)** Women in the experimental group participated in an MBCP program. Eight 2-h group sessions and one 3-h session of silent meditation between weeks 6 and 7. At the beginning of the first session, women received a compact disc of the program for the sessions. They were encouraged to commit to the practices of each session at least six days a week at home. All sessions, except the last one, ended with a review of homework assignments, home practices, and goals for the upcoming week. The subsequent sessions started with a discussion about women’s progress and barriers to reaching their goals in the previous week. Also, their homework assignments

and home practices were checked by a researcher. At the end of the final session, women were encouraged to continue their practices before childbirth, during labor, and after childbirth. Week Summary of the program

One Greetings, background of mindfulness-based childbirth and parenting, awareness of breathing meditation, being with baby meditation, and raisin meditation. Two Body scan meditation, eating meditation, awareness of breathing

meditation, and being with baby meditation. Three Body scan meditation, mindfulness exercises for activities of daily living, awareness of breathing meditation, being with baby meditation, introduction of pleasant events calendar, and pain meditations using pain-coping strategies. Four Yoga, awareness of breathing meditation, formal pain meditation, stress relief meditations using stress-coping strategies, informal pain meditation, being with baby meditation, and introduction of unpleasant events calendar. Five Yoga, awareness of breathing meditation, formal pain meditation, being with baby meditation, mindfulness exercises for activities of daily living, and informal pain meditation. Six Sitting meditation, advance pain meditation using ice cubes, being with baby meditation, mindfulness exercises for activities of daily living, informal pain meditation, selection of a care provider or/and doula, determination of delivery place (i.e., hospital and birth center), introduction of common books about medications, procedures, technology, and physiology of childbirth. Session of silence. Walking meditation, sitting meditation, yoga, body scan meditation, mindful eating, and mindful speaking and listening inquiry practice regarding fears and joys around childbirth. Seven Sitting meditation, body scan meditation, yoga, awareness of

breathing meditation, loving-kindness meditation, being with baby meditation, mindfulness exercises for activities of daily living, informal pain meditation, and reading a book about breastfeeding. Eight Siting meditation, loving-kindness meditation, no technology day meditation, mindfulness exercises for activities of daily living, being with baby meditation, informal pain meditation, selection of a lactation consultant, formal pain meditation, and closing

ceremony. The program was adapted from the intervention developed by Bardacke.

**Ghomshe 2023 (Iran)** For the experimental group, eight group sessions, each in 90 minutes, once a week were held to train self-compassion.

**Guney 2019 (Turkey)** The researchers trained only the pregnant women in the experimental group to count fetal movements on an individual basis. The training was given for ~15–20 min in a single session. The pregnancy training classes of the FHCs were used. After the training was completed, the fetal movement monitoring chart was

given to each pregnant woman. The fetal movement monitoring chart includes a table that will help pregnant women to record their fetal movements daily. The researcher called the pregnant women 2 weeks later to invite them to the FHC to check for the accurate interpretation and regular performance of counting.

**Hajure 2025 (Ethiopia)** This was a non-peer reviewed pre-print. The intervention group received brief CBT sessions. The protocol for CBT was designed by reviewing different manual, guidelines and consultation with the experts in the field of psychiatry and adapted into Ethiopian context. The treatment was offered to participants free of charge. CBT sessions were delivered in a group therapy format and the sessions were offered in successive weeks by trained mental health counsellor. The protocol was then administered to the intervention group in a group format (8 groups, each group consists of 6-8 members),and the sessions were offered once per week for 8 weeks successively, each session lasting from 15-30 minutes in length. Participants in the control condition will visit the antenatal unit based on their scheduled appointments. The first session: Introduction: Beginning with a mental health counselor, getting to know the respondents, describing the fear of birth giving, prenatal attachment and its impact on pregnancy and successful outcomes, summarizing the first session, assigning tasks, and collecting comments. The second session: Reviewing assignments and examining the previous session, familiarizing subjects with cognitive-behavioral training, introducing common mental conditions related to childbirth, identifying respondents anticipations for the intervention and addressing them if needed, educating each phase of labor, assessing and assigning assignments, summarizing the session, and soliciting feedback. Figuring out and overcoming negative or distorted ideas, views, and perceptions related to the session topic. The third session: Evaluating tasks and assessing prior sessions, teamwork, practicing behavioral techniques such as distraction, deep breathing, and progressive muscle relaxation, listening to spiritual songs (mezmur), music, and Quran recitation, cultural approaches for maternal resilience (Qanafa for those practicing the culture and similarly for others also, talking to the unborn child), and behavioral approaches to dealing with childbirth anxiety and sleep problems, summarizing the session, assigning tasks, and providing feedback. The fourth session: Reviewing tasks provided so far and skimming past sessions, final summaries, providing assignments, and receiving feedback.

**Hasanzadeh 2025 (Iran)** In each session from 6 session, the group training was discussed and the mothers were evaluated in group discussions to ensure that they had completely learned the training. First session: Familiarity with anatomical, physiological and hormonal changes in pregnancy, familiarity with fetal growth and development,

counseling about risks in pregnancy and ways to deal with it. Second session: Strategies for adapting more to changes in pregnancy, including familiarity with maternal and fetal attachment behaviors and their benefits to maternal and fetal health, and practical implementation of these behaviors, including attention to fetal movements, fetal imaging, fetal touch, talk. Making the mother with the fetus. Third session: Practicing attachment behaviors that include the mother touching the abdomen, the mother talking to the fetus, calling the fetus by a pseudonym, paying attention to the fetus’s movements on the abdomen, the image of embracing and breastfeeding the baby, exclusive breastfeeding training and how to hold Take the baby. Fourth session: Effects of anxiety on mother and fetus, familiarity with the birth process and its stages. Fifth session: Benefits of happiness in pregnancy and its effects on mother and fetus, training in maintaining the physical and mental health of the mother during pregnancy and beyond, practical attachment behaviors including touching the baby from the abdomen, imagining embracing the fetus, imagining the appearance of the fetus. Sixth session: Overview of previous topics in the form of ROLE PLAY, questions and answers.

**Hassan 2021 (Egypt)** The researcher provided the MFAB booklet (the supportive material) to the participants and discussed the booklet content and answered the questions individually. After that the participants trained and practiced those behaviors: Listening to Quran sound (recited by abdulbasit through mobile phone). Practicing the abdominal palpation to perceive fetal position. Practicing imagining breastfeeding the baby, caring, positive imaging of fetus appearance. Counting fetal movement. Immediately after that, the women’ attachment regarding their fetuses was reassessed via CMFAS and FPAS (post intervention1). Then the researcher provided the participants her contact information and asked them for their phone number. The researcher informed the participants that contact information would be used to contact with her on a private group at what’s up application to remind them of date, place, and time of antenatal visit for collecting follow up post intervention data and for sending videos materials translated into Arabic related to behaviors and benefits of maternal fetal attachment.

**Hoseini 2020 (Iran)** The experimental group received four training sessions based on cognitive-behavioral counseling for 4 weeks.

**Huang 2024 (USA)** This study used two deidentified data files provided by Healthy Birth Day Inc., the provider of the CTK app. The first data file contains records of kick count sessions. Each session is comprised of 10 kick counts. The records show the start and end times of each session as well as the exact time of each count. The second data file contains data from the end of pregnancy survey which CTK users receive 14 days after their due date..

**Jangjoo 2021 (Iran)** The intervention was conducted in groups of 6–8 people. Individuals from the intervention group participated in group counselling, which consisted of four sessions of 60 minutes during the four weeks in the third trimester of pregnancy (weeks 28–34). Sessions Objective Content. First session Getting to know each other and the counsellor, completing the pre-test questionnaires and knowledge about the fertility system and attachment.

(1) Getting to know each other and the counsellor. (2) Familiarization with the rules of the sessions. (3) Explaining and expressing the research objectives for the participants. (4) Familiarization with the changes in anatomy, physiology and hormones, and the effect of these changes in pregnancy, the definition of attachment and its benefits. Second session Getting to know fetal, fetus development stages and factors affecting it, creating attachment.

(1) Familiarity with the development of the fetus in different months of pregnancy. (2) Effect of nutrition and pregnancy care on MFA. (3) Imagining the fetus’ appearance positively. (4) Counting the movements of the fetus

and recording them and giving homework. Third session Improving attachment, identification of signs of pregnancy risk and its treatment. (1) The way MFA is formed. (2) Timing of the beginning of attachment and signs.

(3) Symptoms of risk in pregnancy and ways to deal with and cure them. (4) Talking with the fetus, naming it with an alias. (5) Writing a letter to the fetus. (6) Examining homework. Fourth session Getting to know the stages of delivery and. afterwards, getting to know the infant and its needs, improving enhancement. (1) The way to focus on the fetus and recognize it as an independent entity. (2) Familiarity with the process of delivery and its stages.

(3) Postpartum care. (4) Touching the fetus from the abdomen and guessing the placement of its limbs.

(5) Imagining hugging and breastfeeding the infant

**Jussila 2020 (Finland)** Three interactive 4D ultrasound sessions were offered for the intervention group at 24, 30, and 34 wks. The mothers participated in the sessions alone, but the father of the baby or the mother’s close companion was invited to look at the baby together with the mother, after the session. The sessions lasted approximately 30 min and were performed by an experienced obstetrician and an infant mental health professional working in collaboration. The interactive 4D ultrasound sessions were built on a previous method called “ultrasound consultation”, where the idea is to observe the fetus together with the parent(s); the fetal features, position, sleep-awake rhythm, personal characteristics, activities in the uterus, and responses to mother’s initiatives for interaction.

During the sessions, the pregnant woman was encouraged to explore her thoughts, experiences, and emotions related to watching the fetus on the screen. The aim was to evoke the mother’s active interest in this child and his/her individual perspective, to enhance parental mentalization and maternal-fetal attachment, and hence to support the mother’s emerging parenthood. The Pregnancy Diary To also keep the child more actively in the mother’s mind between the ultrasound sessions, a mentalization-focused pregnancy diary was designed and given to the women in the intervention group. Three prenatal meetings with the infant mental health professional were offered to give an opportunity for the mother to go through her experiences and possible questions that arose while using the diary. The diary meetings were arranged within 2 weeks after each ultrasound session. For each pregnancy week, the diary contained short sections of up-to-date information about the pregnancy phase, fetal development, and health-promoting practices. The key elements were the mentalization-focused questions and tasks inspiring the pregnant woman to think of her experiences of this pregnancy and becoming a parent, to observe the fetus more carefully, and to consider situations from the child’s perspective.

**Kartal 2018 (Turkey)** Within the scope of the 6-week birth preparation training, pregnant women were taught about the anatomy of the reproductive system and the formation of pregnancy, the adaptation of the expectant mother to pregnancy, changes that occur during pregnancy and coping methods, danger signs during pregnancy. Training was given on exercises covering pregnancy and the post pregnancy period, breathing exercises, and relaxation techniques to be used during birth, labor, puerperium and newborn care and family planning.

**Khalili 2020 (Iran)** The intervention group received four sessions of supportive-educational intervention twice a week. Session Content. 1 Getting acquainted with each other, establishing relationships, and discussing domestic violence and its types, violence cycle, and violence during pregnancy. 2 Training and practicing emotional disclosure and emotional release, providing psychological support based on the client-centered theory.

3 Management of marital conflicts, training problem-solving as well as conflict-resolution skills, and reducing the risk of victimization. 4 Maintaining communication with the fetus and training attachment behaviors, sum-up, and review. The intervention was held in a quiet room at the comprehensive care centers. The time of each session varied 60-90 minutes based on the content of each session. At the beginning of each session, the objective was

described. Each session was initiated by reviewing the previous meeting, continued with presenting of the specified

content, answering questions, and clarifying ambiguities and finally, session ended with setting up the next session.

**Kilic 2023 (Turkey)** Study participants in the intervention group were informed about how to use VR. After

performing all adjustments on the headset, the women were asked only to watch the image. It was also informed that each pregnant woman in the intervention group would watch a total of six videos three times a day for 2 days, and watching a video would last at least 5 min, or the women could watch the image at a higher rate if they wanted. All participants in the intervention group watched nature images accompanied by nature sounds with VR headset. In the video, the scenery includes a forest and a lake at the outskirts of a mountain accompanied by the sounds of birds, bees, and water. While watching nature images, the woman feels herself as if she walked in the video.

**Kim 2019 (Korea)** In terms of contents, first, each session was conducted through psychological education and

LKCM training to understand and acquire the subject of each session. This is a form that many meditation practice programs take to convey their purpose and inspire their motivation through pre-meditation psychological education. Second, fetal movement, MFA, maternal acceptance, and healthy childbirth were added in the psychological education to understand and acquire the subjects of each session besides the contents related to stress, mindfulness, and self-compassion during pregnancy. As a result, the subject of each session is composed as follows: pregnancy and a healthy mind (session 1), pregnancy and MFA (session 2), fetal movement and MFA (session 3), acceptance of motherhood (session 4), expansion of love (session 5), healthy birth (session 6). The object of each session is self-compassion meditation training for healthy pregnancy, baby LKCM training for improving MFA, fetal movement LKCM training for improving MFA, imagery LKCM training for acceptance of motherhood, for expansion of LKCM training for pregnant women, and LKCM training for the extension of love for other pregnant women. the

body scan and the banding meditation were combined to be trained each session. A type of visualization (which is characteristic of LKCM was further actively used.

**Kim 2020 (Korea)** Supportive program; Classification (days after hospitalization) Learning objectives Lesson contents Methods Tools/lecturer Time (minute) 1. Information provision (1st, 4th) Understanding the hospital rooms for high-risk pregnant women Information on hospital rooms in the ward for high-risk pregnant women.

1. Providing an educational video and brochure. 2. Q&A- Video- Tablet PC- Brochure/nurse for high-risk pregnant woman 10–30 Obstetrics: the participant can understand and state her diagnosis, cause, treatment, and prognosis. 1. Definition of high-risk pregnancy 2. Treatment related to diagnosis: preterm labor, premature rupture of membrane, placenta previa, gestational diabetes, gestational hypertension 1. Providing materials for the “Maternity school” class 2. Providing Q&A cards for doctors’ rounds- PowerPoint- Tablet PC- Brochure/obstetrics specialist 30–60 Newborn: the participant can understand and state a definition, related diseases, and how to care of premature baby. 1. Definition of premature babies and normal newborn babies 2. Introduction to the method of caring for a premature baby 3. Kangaroo care 1. Providing materials on premature babies 2. Providing Q&A cards for doctors’ rounds- PowerPoint- Tablet PC/pediatrics specialist 30–60 Breastfeeding: the participant is trained in breastfeeding skills and knows the advantage of breastfeeding. 1. Understanding why breastfeeding is important 2. Understanding how to breastfeed 1. Providing an educational video & brochure 2. Lecture 3. Q&A- Video- Tablet PC- Brochure/international breastfeeding certified nurse 30–60 2. Nutritional care (2nd, 4th, 6th). The participant can understand and state a pregnant woman’s needs for nutritional management. 1. Understanding the importance of nutritional management during pregnancy 2. Introduction of proper caloric intake and essential nutrients 1. One-on-one consultations with a nutritionist 2. Watching educational video- Video- PowerPoint/nutritionist 30–60 3. Emotional care (2nd to 10th) Art therapy: the participant can express her feelings and can get emotional relaxation and support. 1. Drawing one’s thoughts about the present state 2. Expressing one’s feelings through consultation 3. Reducing stress through color therapy 1. One-on-one consultation with an art therapist 2. Using a coloring book- Sketchbook- Colored pencil- Coloring book/art therapist 30–60 Music therapy: the participant can reduce her stress and can receive emotional relaxation and support. 1. Listening to live classic music Classification (days after hospitalization) Learning objectives Lesson contents Methods Tools/lecturer Time (minute) 1. Information provision (1st, 4th) Understanding the hospital rooms for high-risk pregnant women Information on hospital rooms in the ward for high-risk pregnant women 1. Providing an educational video and brochure 2. Q&A- Video- Tablet PC- Brochure/nurse for high-risk pregnant woman 10–30 Obstetrics: the participant can understand and state her diagnosis, cause, treatment, and prognosis. 1. Definition of high-risk pregnancy 2. Treatment related to diagnosis: preterm labor, premature rupture of membrane, placenta previa, gestational diabetes, gestational hypertension 1. Providing materials for the “Maternity school” class 2. Providing Q&A cards for doctors’ rounds- PowerPoint- Tablet PC- Brochure/obstetrics specialist 30–60 Newborn: the participant can understand and state a definition, related diseases, and how to care of premature baby. 1. Definition of premature babies and normal newborn babies 2. Introduction to the method of caring for a premature baby 3. Kangaroo care 1. Providing materials on premature babies 2. Providing Q&A cards for doctors’ rounds- PowerPoint- Tablet PC/pediatrics specialist 30–60 Breastfeeding: the participant is trained in breastfeeding skills and knows the advantage of breastfeeding. 1. Understanding why breastfeeding is important 2. Understanding how to breastfeed 1. Providing an educational video & brochure 2. Lecture 3. Q&A- Video- Tablet PC- Brochure/international breastfeeding certified nurse 30–60 2. Nutritional care (2nd, 4th, 6th) The participant can understand and state a pregnant woman’s needs for nutritional management. 1. Understanding the importance of nutritional management during pregnancy 2. Introduction of proper caloric intake and essential nutrients 1. One-on-one consultations with a nutritionist 2. Watching educational video- Video- PowerPoint/nutritionist 30–60 3. Emotional care (2nd to 10th) Art therapy: the participant can express her feelings and can get emotional relaxation and support. 1. Drawing one’s thoughts about the present state 2. Expressing one’s feelings through consultation 3. Reducing stress through color therapy 1. One-on-one consultation with an art therapist 2. Using a coloring book- Sketchbook- Colored pencil- Coloring book/art therapist 30–60 Music therapy: the participant can reduce her stress and can receive emotional relaxation and support. Classification (days after hospitalization) Learning objectives Lesson contents Methods Tools/lecturer Time (minute) 1. Information provision (1st, 4th) Understanding the hospital rooms for high-risk pregnant women Information on hospital rooms in the ward for high-risk pregnant women 1. Providing an educational video and brochure 2. Q&A- Video- Tablet PC- Brochure/nurse for high-risk pregnant woman 10–30 Obstetrics: the participant can understand and state her diagnosis, cause, treatment, and prognosis. 1. Definition of high-risk pregnancy 2. Treatment related to diagnosis: preterm labor, premature rupture of membrane, placenta previa, gestational diabetes, gestational hypertension 1. Providing materials for the “Maternity school” class 2. Providing Q&A cards for doctors’ rounds- PowerPoint- Tablet PC- Brochure/obstetrics specialist 30–60 Newborn: the participant can understand and state a definition, related diseases, and how to care of premature baby. 1. Definition of premature babies and normal newborn babies 2. Introduction to the method of caring for a premature baby 3. Kangaroo care 1. Providing materials on premature babies 2. Providing Q&A cards for doctors’ rounds- PowerPoint- Tablet PC/pediatrics specialist 30–60 Breastfeeding: the participant is trained in breastfeeding skills and knows the advantage of breastfeeding. 1. Understanding why breastfeeding is important 2. Understanding how to breastfeed 1. Providing an educational video & brochure 2. Lecture 3. Q&A- Video- Tablet PC- Brochure/international breastfeeding certified nurse 30–60 2. Nutritional care (2nd, 4th, 6th) The participant can understand and state a pregnant woman’s needs for nutritional management. 1. Understanding the importance of nutritional management during pregnancy 2. Introduction of proper caloric intake and essential nutrients 1. One-on-one consultations with a nutritionist 2. Watching educational video- Video- PowerPoint/nutritionist 30–60 3. Emotional care (2nd to 10th) Art therapy: the participant can express her feelings and can get emotional relaxation and support. 1. Drawing one’s thoughts about the present state 2. Expressing one’s feelings through consultation 3. Reducing stress through color therapy 1. One-on-one consultation with an art therapist 2. Using a coloring book- Sketchbook- Colored pencil- Coloring book/art therapist 30–60 Music therapy: the participant can reduce her stress and can receive emotional relaxation and support. 1. Listening to live classic music 2. Explanation of stories about each piece of music: the meaning, background, and composer 1. Performance of classical music for a small group- Classical music/talent donation group 60 Fetus diary: the participant is encouraged to improve maternal-fetal attachment and can tell how much she is anticipating the delivery and baby. 1. Looking back on one’s feelings and thoughts about the fetus and oneself in a day 2. Sharing one’s feelings about the delivery and fetus 1. Writing a fetus diary 2. One-on-one consultation- Diary/nurse in high-risk pregnant woman 30–60 4. Exercise care (3rd to 10th) Education on bed exercise; the participant can activate her physical abilities and engage in muscular strengthening before delivery. 1. Understanding the importance of exercise during pregnancy 2. Introduction to the bed exercise program for high-risk pregnant woman 3. Exercise types are divided into neck, arm (hands, wrists, elbows, shoulders), and leg (foot, ankles, knees) exercises. 1. Following the bed exercise video 2. Providing compression stockings 3. Q&A 4. The participants were given exercise types and times adjusted according to their condition. - Video- Compression stockings/ nurse for high-risk pregnant women 30 1. Listening to live classic music 2. Explanation of stories about each piece of music: the meaning, background, and composer 1. Performance of classical music for a small group- Classical music/talent donation group 60 Fetus diary: the participant is encouraged to improve maternal-fetal attachment and can tell how much she is anticipating the delivery and baby. 1. Looking back on one’s feelings and thoughts about the fetus and oneself in a day 2. Sharing one’s feelings about the delivery and fetus 1. Writing a fetus diary 2. One-on-one consultation- Diary/nurse in high-risk pregnant woman 30–60 4. Exercise care (3drd to 10th) Education on bed exercise; the participant can activate her physical abilities and engage in muscular strengthening before delivery. 1. Understanding the importance of exercise during pregnancy 2. Introduction to the bed exercise program for high-risk pregnant woman 3. Exercise types are divided into neck, arm (hands, wrists, elbows, shoulders), and leg (foot, ankles, knees) exercises. 1. Following the bed exercise video 2. Providing compression stockings 3. Q&A 4. The participants were given exercise types and times adjusted according to their condition. - Video- Compression stockings/ nurse for high-risk pregnant women 30 2. Explanation of stories about each piece of music: the meaning, background, and composer 1. Performance of classical music for a small group- Classical music/talent donation group 60 Fetus diary: the participant is encouraged to improve maternal-fetal attachment and can tell how much she is anticipating the delivery and baby. 1. Looking back on one’s feelings and thoughts about the fetus and oneself in a day 2. Sharing one’s feelings about the delivery and fetus 1. Writing a fetus diary 2. One-on-one consultation- Diary/nurse in high-risk pregnant woman 30–60 4. Exercise care (3rd to 10th) Education on bed exercise; the participant can activate her physical abilities and engage in muscular strengthening before delivery. 1. Understanding the importance of exercise during pregnancy 2. Introduction to the bed exercise program for high-risk pregnant woman 3. Exercise types are divided into neck, arm (hands, wrists, elbows, shoulders), and leg (foot, ankles, knees) exercises. 1. Following the bed exercise video 2. Providing compression stockings 3. Q&A 4. The participants were given exercise types and times adjusted according to their condition. - Video- Compression stockings/ nurse for high-risk pregnant women.

**Koh 2021 (Korea)** A couple-centered antenatal education program was developed to help couples’ adaptation to become parents and their relationship. The environmental interaction model of the process of becoming a mother was used as a theoretical basis for the educational program. A couple-centered psychoeducation method was selected. Two main content areas: adaptation of the couple relationship and becoming parents for couples. The content on couple-relationship adaptation included emotional intimacy, support, respect, empathy, gratitude, and emotional exchange between couples from pregnancy to the postpartum period as ways to deal with marital problems and difficulties. The content dealing with becoming parents included promoting well-being in response to physical and mental changes, discomfort, depression, and stress, self-care, parental-fetal attachment, preparation for normal delivery, and preparation for the parenting role (e.g., newborn baby care and breastfeeding). Each session set 2 to 3 specific goals for couples, such as social-role preparation, instructions on infant caregiving, and the promotion of fetal attachment, self-care, and well-being. Educational program in four sessions, each of which lasted for 1 hour in the childbirth education room of the hospital. The size of each intervention group was decided based on couples’ needs, with the education provided to one to three couples at a time. The teaching method consisted of face-to-face education and counseling. Throughout the session, we provided education and information to help the couples discuss these topics, allocated time to practice skills such as expressing one’s own emotions and effective conversation techniques and provided time for questions and answers. In addition, a small gift was presented to encourage participants to continue participating in the program, and periodic text messages were sent as reminders before scheduled appointments and to express appreciation for the participants’ participation. Goal based on the ecological interaction model Methods Content Session 1: Becoming happy parents (1 hour by a midwife) Social-role preparation Education, information, practice, Q&A Happy environment, relieving stress, and participation of the husband Attachment promotion: parental-fetal attachment Talking and singing with finger movement for the fetus.

Self-care promotion: preparation for a normal delivery Education, information, practice, Q&A Normal delivery process and husband roles Method of how to breathe, relax, and massage to relieve the pain Session 2: Becoming competent parents (1 hour by a midwife) Social-role preparation Education, information, Q&A Newborn care in growth and development, normal reflex reactions, and getting used to baby signals, father–child care Infant-caregiving instruction Education, information, Q&A Advantages of breastfeeding (for mother and baby), and breastfeeding method, baby’s signals, and deep breastfeeding (latch and positioning) Practice, Q&A

Breast massage and breastfeeding, holding a baby, feeding, and burping, soothing a crying baby, changing diapers, bathing, and managing the umbilical cord Session 3: Becoming a happy couple and family (1 hour by a women’s health nurse) Well-being promotion Education, information, conversation, Q&A 1. Physical and mental changes in the pregnant woman and her husband 2. Check the mother’s and father’s situations during the perinatal period and talk about each other’s difficulties and how to help, and how to maintain the marital relationship Experience

1. Husbands wear pregnancy simulator vest Conversation practice 1. Identify usual communication patterns, and practice positive self-assertive communication Self-care promotion Education, information, Conversation, Q&A

1. Physical changes, and discomfort of pregnant women, and caution and self-care methods, importance of spousal support during the third trimester 2. Prenatal and postpartum depression, stress related to the parental transition in couples, the importance of mutual support for couples, and self-care for couples, express their feelings with each other on parental transition Session 4: Becoming a happier couple (1 hour by a women’s health nurse)

Well-being promotion Education, information, conversation practice, Q&A 1. Understanding and empathy between spouses, and the effects of the virtuous cycle of expressing gratitude and empathy 2. Identifying common marital problems in parents and discuss current marital problems or difficulties 3. Characteristics of well-adapting parents, the positive effects of hugging, and consideration for each other Conversation practice 1. Thank-you messages (conversations and letter-writing) 2. Talk about what they want from others Social-role preparation Education, information, conversation Q&A 1. Importance of accepting and adapting to parenthood in a positive manner

2. Talking about the difficulties, burdens, and fears of childbirth.

**Kordi 2016 (Iran)** The experimental group received one session of guided imagery regarding maternal role in

34th week of gestation in groups of four and seven. Guided imagery was taught through training classes for the mothers at health centers that had comfortable chairs and were sufficiently quiet. The techniques and the effects of guided imagery were taught to the subjects. Afterwards, the mothers were asked to take a few deep breaths, switch off their phones, and focus their attention on the CD of maternal role imagery and imagine their maternal role throughout the mental imagery, which lasted for 20 minutes. Thereafter, the researcher gave the guided imagery CDs to the mothers, and they were asked to perform guided imagery at home twice a week for two weeks. The researcher followed the subjects through phone calls in terms of performing guided imagery.

**Laurent 2025 (USA)** Adapted Mindfulness-Based Stress Reduction (MBSR), MBCP includes the core elements and structure of MBSR modified for the perinatal context, in addition to psychoeducation components to prepare parents for the demands of childbirth and parenting a newborn. Over the course of 9 weeks, groups of child-bearers and their partners meet with a birthing and mindfulness teacher for weekly three-hour classes and to engage in a full-day retreat, for a total of 33 h of instruction. In addition, participants are invited to engage in about an hour of daily home practice. Course content includes perinatal psychoeducation, childbirth and parenting skills development, group discussion, informal “mindfulness in everyday life” practices; and four types of formal meditations—focused attention, open monitoring, movement, and loving-kindness. Participants in the MBCP condition were assigned to one of four in-person and/or online Zoom MBCP classes. All community classes included psychoeducation content

on prenatal health, childbirth, pain management during labor, and postpartum and newborn care. Most classes also included brief psychoeducation on perinatal mental health. While most community classes included instruction on relaxation-based pain management techniques and some included visualizations and/or meditations, classes were screened by a research coordinator to ensure that they did not explicitly incorporate mindfulness into their curriculum. Methods of delivery ranged from one-on-one in-person or Zoom classes, group in-person or Zoom classes, and online platforms that provided participants access to videos and other educational materials that participants could consume at home at their own pace. Total instruction time ranged from 2–20 h.

**Lavi 2015 (USA)** During pregnancy, treatment focuses on woman’s experience of her pregnancy and her fantasies, fears, attributions, and hopes for her unborn child. Throughout treatment, the following intervention strategies are used: psychoeducation on infant development and the impact of intimate partner violence on the fetus/baby, body-based and mindfulness-promoting strategies to help women to be aware of and tolerate negative body sensations, reflective developmental guidance, insight-oriented interpretation, and concrete assistance with problems of living and crisis intervention. Fidelity of the treatment was monitored through weekly supervision with one of the treatment developers based on adherence to the perinatal CPP protocol. Participants assigned to the community class condition were provided a list of online and in-person birthing classes available locally and nationally to choose from.

**Lee 2002 (Korea)** providing visual information by ultrasound.

**Lee 2023 (Korea)** All participants underwent 3D fetal ultrasonography and were shown the fetal images on an

ultrasonography monitor: however, only the VR intervention group was shown fetal images that merged ultrasonographic data to produce VR images; these were viewed in a headset. All participants received the prenatal-coaching mobile app when they were enrolled. This prenatal-coaching app provides services that enable submitting results for maternal weight and blood pressure and checking changes with intuitive graphs, and it includes a glucose-monitoring diary for participants with gestational diabetes mellitus or pregestational diabetes mellitus. Relevant information from authoritative guidelines on helpful activities (e.g., yoga, exercise, and stretching) and a recommended diet were supplied according to gestational age. All participants viewed 3D ultrasonography images; however, the VR experience and the fetal images produced in VR were provided only to the VR intervention group. All the images generated in VR were sent to the prenatal-coaching mobile app, so that the users could see the images whenever they wanted. The users could share the saved pictures of their fetus with their family members and monitor growth via measurements of fetal body parts. Progress in fetal growth at each ultrasonography examination was demonstrated in graphs that were saved in a private library folder. The users could compare the fetal image with everyday objects such as apples to help understand the actual size of the fetus (Figure 4A). In addition, the VR experience allowed the participants to actively engage their imagination about their expected baby by modifying specific structures of the fetal face, such as the eyes, nose, mouth, and cheeks, by touching the screen.

**Loughnan 2019 (Australia)** Participants allocated to iCBT logged in to their individual Virtual Clinic account, completed baseline questionnaires and started Lesson 1. All three lessons were required to be completed within four weeks. The MUMentum Pregnancy program is a brief unguided iCBT intervention tailored specifically to women experiencing generalized anxiety and depressive symptoms in the antenatal period. The program was adapted from our validated six-lesson, clinician-guided iCBT program for mixed anxiety and depression. course content was condensed and presented over three lessons rather than six, with a key focus on introducing women to core CBT

skills to help manage anxiety and depressive symptoms. Content for this program was presented in the form of a shortened illustrated story, in which two fictional characters experiencing anxiety and depression during their pregnancy learn to self-manage their symptoms which improve with CBT skills practice. Each lesson consisted

of a set of lesson slides depicting the characters’ stories and introduction to core CBT skills (e.g., thought challenging); a brief lesson summary and action plan to implement skills; and a range of supplementary resources. Lesson Skills Extra resources 1 •. Psychoeducation: o. About anxiety and depression o. Identifying symptoms

o. Cognitive behavioral model o. Prioritizing self-care o. Physical symptoms o. Partners and supporters

•. Controlled breathing •. Progressive muscle relaxation •. Medication for anxiety and depression during pregnancy and breastfeeding •. Sleep hygiene •. Fight-or-flight response •. Pleasant activities •. FAQs •. Further skill examples

2 •. Psychoeducation: o. About thoughts o. Identifying unhelpful thoughts o. Shifting unhelpful thoughts

o. Accepting uncertainty •. Thought challenging •. Coping cards •. Structured problem-solving •. Understanding intrusive thoughts and images •. FAQs •. Further skill examples 3 •. Psychoeducation: o. Unhelpful behaviors (low

activity; avoidance) o. Facing your fears •. Activity planning and monitoring •. Graded exposure •. Assertive communication •. Relapse prevention •. Self-care plan •. FAQs •. Further skill examples

**Mahmoudi 2021 (Iran)** The trained group included the pregnant mothers who received the MFA-based training plus the routine prenatal care. Ninety minutes of training once every 3 days for a total of three sessions was performed in small groups (3–5 mothers). For performing the training at home, the booklet of MFA-related behaviors along with necessary explanations provided in a CD including the recorded fetal heart sound,

animation film of fetal development and relaxation guide, and a check list to follow performance regarding attachment-related behaviors at home were given to the mothers. The researcher’s phone number was also given to them to ask any questions and probable problems. The mothers were asked to perform the training program

daily for 4 weeks and the trainer called them weekly and reminded them regarding the practices. Sessions Training content 1 Anatomical and physiological changes in pregnancy and effects of changes, the concept of attachment to

the fetus and its advantages, the concept of relaxation and its benefits and relaxation training (breathing

pattern) 2 Stages of fetal development, process of labor, performing attachment behaviors including listening to the sound of fetal heart, talking to the fetus, calling the fetus with a name, looking at the abdomen and paying attention to fetal movements, drawing the fetus and preliminary relaxation training 3 Principles of nutrition in pregnancy, performing attachment behaviors including touching the fetus and caressing it from the abdomen, counting fetal movements, looking at the fetus in the sonographic photo, imagining hugging and breastfeeding the baby and relaxation training through imagination about the fetus.

**Maleki 2025 (Iran)** WhatsApp, was considered as a training platform. Mothers were required to study the educational files for 3 days after uploading. Throughout this period, the researcher monitored the progress of each session’s study file and the completion of homework by the mothers. Feedback from the educational sessions was collected, and once it was confirmed that the materials from the previous session were studied and homework was completed, the materials for the next session were uploaded on a weekly basis. Additionally, besides creating a group on WhatsApp for uploading educational files offline, the researcher established another group for discussions, allowing mothers to engage in conversations, provide feedback, and communicate easily with the researcher. The researcher utilized various methods, including telephone communication, to maintain constant contact with research participants, addressing their questions and resolving issues within the WhatsApp group. Mindfulness training generally followed the program known as MBSR. Sessions were shortened to 90–120 min over the course of 8 weeks. Teaching materials started with an emphasis on attention and concentration and then were followed by practical exercises such as deep breathing so that focused attention was also used in breathing. Session 1: During this session, instruction focused on the characteristics of a wandering mind, outlining mindfulness assumptions and definitions, and highlighting the significance of spiritual health education during pregnancy. The overarching theme of momentary awareness and acceptance of desires and their effects (e.g., stress, anxiety) was introduced and consistently reinforced throughout the training. Session 2: During this session, techniques for managing thoughts and increasing awareness of negative thoughts were explained. It was emphasized that it is not possible to completely stop negative thoughts or control them forcefully. Instead, the focus was on observing thoughts and feelings without judgment and accepting them as they are, recognizing that they are merely mental events. These thoughts and feelings come and go and do not necessarily reflect reality. Participants learned how to avoid falling

into rumination patterns and how to change their behavior in constructive ways to improve their lives. Additionally, the session covered topics related to happiness, positive thinking, and foresight in the spiritual development of mothers. Session 3: In this session, participants were taught the technique of using judgment criteria and how to

determine a personal timeline, including the distant future, near future, present time, distant past, and recent past. It was explained that constant and nonjudgmental observation of unpleasant thoughts and feelings, such as stress and anxiety, without attempting to escape or avoid them, can reduce emotional reactions. Participants learned that

focusing too much on the past or future can increase negative emotions and thoughts, causing a loss of focus on the present moment. Additionally, the session covered the topics of hope, having a sense of purpose, and fostering connection with God, all within the context of the mother’s spiritual development. Session 4: In this session, participants were taught the techniques of body scanning and mindful eating, specifically eating raisins with conscious attention. The session also covered topics related to self-knowledge and modifying behaviors or thoughts that indicate pessimism, instability in life, or incompatibility between values, interests, and attitudes, all of which contribute to the mother’s spiritual growth. Session 5: Participants were encouraged to use mindfulness

practice in everyday life, including “awareness of breath” meditation and mindful walking (“four modes of walking”), during which the individuals practiced systematically noting objects they see and then objects they hear, then objects they smell, and then tactile objects such as the pressure of their feet on the ground. Believe in God, self-sacrifice, and trustworthiness related to mothers’ spiritual growth were also instructed in the fifth session, too. Sessions 6–8: These sessions focused on several key techniques and principles aimed at the spiritual development of the mother and her family. Participants were trained in a 3-min breathing exercise, referred to as a “mini meditation.” This exercise, incorporating elements of full meditation sessions, aimed to integrate mindfulness into

everyday life. By redirecting awareness to the breath at any moment of the day, individuals could increase self-awareness and insight, reducing habitual and automatic behaviors. This exercise involved sitting on a chair, closing the eyes if possible, and paying full attention to the body’s position and sensations. Participants then focused on their breathing, inhaling, and exhaling in succession. Breathing was emphasized as a passage to the present moment, promoting awareness and relaxation. Finally, attention was expanded to the whole body, leveraging the sensations created during breathing to enhance the overall experience. Engaging in worship and avoiding sinful behaviors was emphasized for their roles in spiritual practice. Cultivating attachment to the child and observing halal nutrition were highlighted for their significance in the mother’s spiritual growth. Additionally, the technique of accepting both pleasant and unpleasant experiences was taught. Topics related to the spiritual growth of the family included respect and intimacy with the spouse, gratitude, honesty, and forgiveness. Conscious attention to the wise mind was explained using metaphors. Participants practiced these exercises once a day at home for 40 min, with the 3-min breathing exercises practiced three times a day. Mindfulness training was increased from daily sessions of 10

min to 40 min from the fourth session onwards. From the fourth session, 10min of daily breathing

concentration and then formal mindfulness exercises in a sitting position were introduced for more concentration.

The seventh session focused on the concept of the “wise mind,” discussing the importance of consciously paying attention to this inner wisdom. It also covered the importance of social responsibility and cultivating love for

others as part of the spiritual growth of society. By incorporating these techniques and principles, participants were encouraged to integrate mindfulness and spiritual practices into their daily lives, enhancing their personal and

family well-being. The tools and ways of maintaining things learned in the future were taught. Feedback was

received from mothers after each session, and assignments were also used as a combination of formal Meditation Training meditations to reduce habitual mind-wandering and strengthen their attentional capacities, as well as loving-kindness and awareness of breath. Focusing attention on the breath is intended to help individuals retrain their minds from habitually engaging in self-related preoccupations to more present-moment awareness, as well as informal practices (four modes of walking, mindfulness of daily activities, mindfulness RAIN). The RAIN exercise is a brief audio-guided mindfulness exercise. The participants practiced the techniques of the previous session and

could ask their questions, which were discussed in the group.

**Marzouk 2015 (Egypt)** The Intervention group was provided with the training program. It entailed training on palpating the fetal parts using Leopold's Maneuvers and counting the fetal movements. The program was provided through two sessions. Session one It was theoretical; using a power point presentation MFA concept, benefits of improving MFA and the different skills for enhancing MFA were clarified. During the same session, a videotape about applying Leopold's Maneuvers and counting the fetal movements had been shown. This session was conducted in a separate room in the clinic for approximately 30-45 minutes on small groups (n= 3-6). Session two It was started immediately after the first session in the same room and took approximately 10-15 minutes for each participant. During this session each participant was individually trained by the nursing researcher on performing the Leopold's Maneuvers through the following steps: instructing the client to void and lie in supine position, then the researcher took the hand of each participant to help in touching the fetal buttocks and back together with one hand and fetal limbs with the other hand, so that expectant mothers were actually able to touch the fetal body parts. The enrolled pregnant mothers were asked to perform this procedure once weekly. During the same session the enrolled pregnant mothers were trained on counting the fetal movements using Cardiff method, that consists of instructing the pregnant woman to empty her bladder, drink a cup of juice and immediately lie on left side and start counting the fetal movements. The enrolled pregnant mothers were asked to perform this procedure once daily, the findings were recorded on fetal movements counting chart and provided to the researcher at the next antenatal clinic visit to monitor clients' compliance with the skill performance.

**Masroor 2008 (Pakistan)** They were then briefed on fetal development, and maternal-fetal interaction and usefulness of ultrasound as a diagnostic modality. Both groups were shown the fetus during their ultrasound

examinations and important anatomical structures were pointed out.

**Mesgarzadeh 2020 (Iran)** After training the fetal movement counting to the intervention group, they all received fetal movement counting form and a ribbon. They were asked to count and record fetal movements every day for two weeks and tie the ribbon.

**Mikhail 1991 (USA)** Both the Sadovsky chart (counting three times a day after meals) and Cardiff chart (counting the first 10 movements each morning) were used.

**Mohapatra 2021 (India)** Fetal movement count training was implemented on experimental group. The training includes both instructions and demonstration. Booklet contained the steps of fetal movement count and 28 days counting chart was provided to the pregnant women to make them adhere to the count. Mothers were followed through phone calls.

**Mojahed 2019 (Iran)** The groups participated in the six 90-minute consecutive weeks in morning and evening. Session Content of session 1 Introducing the participants, completion of the questionnaire, explanation of the details of the project, such as the primary and secondary objectives of the training, providing knowledge on the physiological and hormonal changes in pregnancy, and its impact on the mind of pregnant women, describing the mother's adaptation to the changes during the pregnancy. Practical exercise: Touching the abdomen of the mother and paying attention to movements of the fetus on mother's abdomen Home exercises: Touching and recording the number of fetal movements at three times of morning, evening, and night for a total of one hour at home and recording them on a designed chart. 2 Providing knowledge on the growth of fetus and explaining the maternal-fetus attachment and the way of understanding these dimensions and using them to communicate with the fetus, teaching and practicing attachment techniques (drawing pictures, etc.) Practical exercise: touching the abdomen by the mother, counting the movements of the fetus by the mother on her abdomen, guessing the position of the fetus and imagining the appearance of the fetus. Home exercises: Touching and recording the number of movements and determining the position of the fetus at three times of morning, evening, and night for a total of one hour at home and recording them on a designed chart. 3 Group counseling on pregnancy stress and pregnant women's experiences during this period, explaining marital relationships during pregnancy, counseling about risk symptoms in pregnancy and ways to cope with them and training the ways of removing the stress and reduce the anxiety. Practical exercise: touching the abdomen by the mother, mother's communication with the fetus, calling the fetus with allonym, paying attention to the abdomen and movements of the fetus on her abdomen Home exercises: Touching and recording the number of movements and determining the fetus position at three times of morning, evening, and night for a total of one hour at home and recording them on a designed chart. 4 Investigating the pregnancy stress and teaching methods for coping with it and teaching the ways of removing the stress and reducing the anxiety and intellectual errors Practical exercise: Performing stress removing exercises, touching the fetus on her abdomen, guessing the position of fetus on her abdomen, mother's communication with the fetus, calling the fetus with allonym, paying attention to the abdomen and movements of the fetus on her abdomen Home exercises: Touching and recording the number of movements and determining the fetus position at three times of morning, evening and night for a total of one hour at home and recording them on a designed chart, guessing the position of the fetus and imagining the appearance of the fetus 5 Practical exercise: Performing stress removing exercises, touching the fetus on her abdomen, guessing the position of fetus on her abdomen, mother's communication with the fetus, calling the fetus with allonym, paying attention to the abdomen and movements of the fetus on her abdomen, counting the movements of the fetus, relaxing the fetus by touching the abdomen, imagining hugging the neonate and breastfeeding Home exercises: Daily touching and recording the number of movements and recognizing the fetus position at three times of morning, evening and night for a total of one hour at home and recording them on the designed chart, as well as guessing the position of the fetus and imagining appearance of the fetus 6 Summing up of the attachment discussion, providing a report on the implementation of maternal-fetal attachment by pregnant women.

**Mokaberian 2021 (Iran)** The experimental group underwent 30-minute sessions of progressive muscle relaxation as well as mental imagery-based relaxation exercises twice a week for a total period of 8 weeks. In the first 20 minutes, for the physical relaxation purpose, the mother was placed in a comfortable position and then started the exercises by entering concentration in the form of deep and slow abdominal breathing. This step involves contracting and then consciously releasing the muscles. Then, relaxation was done through mental imagery in the final 10 minutes. At this point, the mother was imagining a scene in which she felt safe, relieved of tension, and anxiety. Then, with appropriate music and the therapist’s voice, a pleasant atmosphere was established being of interest to the patient.

**Mokaberian 2021 (Iran)** The fathers and mothers of the experimental group underwent prenatal care training by midwives during 8 sessions of 20 - 30 minutes, and the women in the control group underwent the same training without the presence of their husbands.

**Monickaraj 2020 (India)** Individualized video-assisted maternal training for 07 minutes was given. Pregnant women in the interventional group were educated to lie down in the left lateral position for half an hour thrice a day (i.e., morning, noon, and evening) to concentrate on the perceived fetal movements by engaging in the maternal-fetal interactional activities such as talking to the baby, reading for the baby, singing for the baby, playing music for the baby and caressing their abdomen to promote MFA. Documentation of the maternal fetal interactional activities practiced was done in the calendar to monitor activities of MFA that was provided to the pregnant women. Review and reinforcement of individualized maternal training was given to the experimental group through telephonic follow-up once a week for three consecutive weeks.

**Murphy-Tighe 2025 (Ireland)** The intervention group attended four 1-hour weekly online MuM sessions. Each session followed a thematic approach with supplementary resources emailed after each session. Open communication was encouraged through weekly check-in emails and an option to arrange a phone call with a member of the MuM team if any sensitive issues arose during sessions. First session: Relaxation and Wellbeing: managing perinatal stress, anxiety, and physical discomfort. Live music for relaxation. Recorded music for relaxation/ mindfulness/ regulation. How to choose and use music to relax, self-care, and manage stress/anxiety during pregnancy. Creating a customized playlist. Discussion re: future hopes. Session 2: Bonding and lullabies. Live music for relaxation. Songs for connecting with baby. Lullabies. Attachment theory and bonding. Discussion about bonding with baby. Benefits of singing lullabies during pregnancy. Session 3: Preparation for childbirth. Live music for relaxation. Using music to support breathing & relaxation. Guided meditation. Thinking about own tools and resources for childbirth. Practical information on preparing playlists and equipment. Creating different playlists for different stages of childbirth. Tips on expressing participants’ wishes to their healthcare team. Session 4: Preparing for the parenting journey. Live music for relaxation. Toning/humming. Singing. The good enough parent. Discovering, allowing, and accepting feelings/ emotions. Thoughts about parenthood.

**Muzik 2012 (USA)** Each 90-min session focused on a variety of poses, all taught specifically for the pregnant

body, with awareness of the baby and modified for any level of experience or gestational age. Classes met once per week. Two 10 session mindfulness yoga classes ran consecutively, each with 10 participants who completed the full series of sessions; one class took place during evening hours and the other during the day. The M-Yoga curriculum differed from typical prenatal hatha yoga classes by highlighting mindfulness practice, with targeted instructions,

reminders, and readings. Participants were taught mindfulness techniques including breathing, guided visualization, and relaxation. The instructors consciously made it a part of class to continually remind women to focus inward toward the sensations of their body, listen to the feedback of body during asana, and be aware of how their bodies are changing to support their growing baby. A significant aspect of the intervention was being "mindful" of the baby, to sense its unique persona, which in turns facilitates the attachment process. In addition, having the word "mindfulness" in the title of the class/ study continually brings that awareness to the forefront. Sessions opened with a 10-15 min check-in, allowing each woman to share how she was feeling physically and emotionally with the group. Teachers customized the poses and instruction to address participants’ current issues, cultivating a supportive atmosphere. For example, if irritability was an issue, instructions included finding compassion towards oneself as the body experiences the hormonal changes of pregnancy. If heartburn was an issue, poses were taught with an emphasis on creating more space between the chest cavity and throat. Each class included seated, kneeling, standing, and restorative poses. The instructors demonstrated each pose while including descriptions of seven qualities of mindfulness: allowing presence, non-judging, patience, beginner’s mind, non-striving, acceptance and letting go. The instructors used directives, such as “Practice the pose for your body without judgment” and “Bring your attention to your breath, always practicing with awareness of your growing baby”. Techniques supported women’s comfort with their changing body, prepared the body and mind for the birthing experience, and focused awareness on the fetus. To ensure women’s safety, modifications for all poses were offered and participants were reminded throughout the practice to listen to the body’s cues, when to move deeper in a pose and when to slow down. Classes ended with a 15-20 min restorative pose, including a full-body relaxation exercise, along with a reading from a text such as Everyday Blessings: The Inner Work of Mindful Parenting. Finally, sessions concluded with a 5-10 min informal interaction while participants prepared to leave. Instructors and participants did not interact outside the class context, and we did not facilitate peer interactions among participants outside the M-Yoga class. Women were encouraged to also practice at home, but compliance was not assessed.

**Nasab 2023 (Iran)** Then, the intervention group, in addition to routine healthcare, received attachment counseling sessions. Along these lines, based on their convenience for attendance, the intervention group members were divided into 3 groups of group counseling (one 10 and two 11 member groups) based on the proposed time to attend counseling sessions. Four weekly one-hour cognitive-behavioral counseling sessions were held for each group.

**Nwogu 2011 (Nigeria)** The patient lies on the couch for the ultrasound examination. The measurement of the fetal parts was then taken as well as the rest of the information needed by the obstetrician/ clinician that referred her for the scan. The fetal ultrasound image was then explained and discussed with the mother familiarizing her with the basic anatomy of the fetus by pointing out some of the physical features and organs of the fetus like the head, face, limbs, heart etc. Conversation between the mother and the sonographer is encouraged by the latter allowing the mother-to-be (and father-to-be, if he was there) to ask questions. This extra procedure took about 3 minutes for each participant. It is this learning process the mother undergoes about the fetus and the scan in general that we call the „Educational Process‟. This educational process was standardized in the sense that the explanation and discussion of the fetal image to the mother was the same for all the subjects, to ensure uniformity.

**Ohman 2010** **(Sweden)** ultrasound examination including screening for DS was offered in gestational weeks 12–14.

**Okyay 2025 (Turkey**) Training was given to the expectant fathers in the experimental group using visual materials on how to perform the first and second Leopold maneuvers. At the beginning of the practice, how to perform the maneuvers was explained theoretically with materials prepared using colored pictures, and then, it was explained practically on the expectant mothers. In the first phase of the practical demonstration, the first and second Leopold maneuvers were applied by the researcher, and immediately afterward, the practices were carried out with the expectant father. Each couple was provided with a stethoscope to listen to fetal heartbeats, and they were instructed to listen to fetal heartbeats for 5–10 min together (using the same stethoscope alternately, first the father, then the mother), at least once a day, for 15 days, by performing the Leopold maneuvers. To record the number of fetal heartbeats they listened to, a fetal heartbeat recording chart prepared by the researchers was given to the couples. At the end of the first meeting, an appointment was made for the second stage (15 days later), and the first stage of the study was completed in approximately 10–15 min for each couple.

**Ozbek 2022 (Turkey)** pregnant women included in the experimental group were called before each home visit so that haptonomy could be done at home. All home visits, including haptonomy, lasted 60–90 min. Three applications of the sessions were applied one-on-one with the pregnant woman. Afterwards, training brochure prepared by the researcher and training video shot on the model were given to the pregnant woman, and she did the last 4 applications with her spouse at home. Haptonomy was performed by individual home visits to pregnant women. At the beginning of home visit, information was given about definition, purpose, and importance of haptonomy. It has been informed that haptonomy will be applied once a week for at least 30 min on the day and time they want for 7 weeks, for the first 3 weeks, with the researcher, and for the next 4 weeks, with the help of the haptonomy training brochure and video. The pregnant woman’s questions were answered, and the environment for haptonomy was prepared (a calm, quiet room was chosen, cushions were placed on the floor opposite to each other, back of the pregnant woman was supported so that she could be comfortable). The environment was prepared in the same way for each application. Stages of application: 1. For the application, a face to face sitting position was taken. 2. To be used as a standard throughout the research, a piece of music was started to be played, after which expert opinion was taken. 3. The application was made with the eyes of the pregnant woman closed. 4. The practice started with breathing exercises. With abdominal and chest breathing, the body was relaxed. 5. Energy work and dreaming phase was started. The researcher and pregnant woman put their left hands on each other’s hearts. The goal is to feel the energy, feel the heart, open the third eye, and prepare the hands. In this study, it was aimed to increase the confidence of the pregnant woman in her own feelings and body by getting to know her feelings and body. Then, the researcher and pregnant woman placed their right hands on their foreheads while their left hands were on their hearts. Thus, it was ensured the pregnant woman visually felt the presence of the baby. It was ensured that her communication with her baby is in spiritual dimension as well as the physical. 6. Then, the stage of touching and communicating with the baby was initiated. Hands of the researcher and pregnant woman were placed on the pregnant woman’s womb. Thus, it was ensured that the baby could recognize the warmth and energy of the pregnant woman’s hands. Then, the first call of the pregnant woman to her baby was provided. The aim is to enable the baby to recognize the mother’s voice. This call to the baby was repeated 2–3 times. 7. By touching the womb of the pregnant woman with her, exercises to adapt to the fetus were applied. The aim is to increase awareness of pregnant woman’s feelings and to enable her to play intrauterine games with her baby (such as calling out to the baby, shaking her abdomen, and making the baby move to the touched area with the mother’s voice). Thus, she was supported to carry and communicate with her baby with love and encouraged. With touching, mother’s reactions to the baby and

baby’s reactions to the mother were followed. In this way, the bond between pregnant woman and baby was strengthened, helping the mother to recognize baby’s movements and her own feelings. 8. Haptonomy was terminated with breathing exercise.

**Park 2018 (Korea)** Experience-focused prenatal program. Session Subjects Contents Method Time/min 1st week Normal pregnancy ․Introduction the program ․Communication each for stress, anxiety ․Normal pregnancy process Pretest, Lecture: PPT 50 Rest ․Offer of beverage 10 Experience-focused education ․Fetus nicknames & self-introduction ․1 stage of labor slow respiration & relaxation practice Practice Discussion 30 10 2nd week Naturalism birth care ․Comprehension of birth ․Premonitory signs of labor ․Admission sign ․Breathing & position of delivery Lecture: PPT, video 50 Rest ․Offer of beverage 10 Experience-focused education ․Feedback ․1 stage of labor rapid respiration & relaxation practice ․2 stage of labor pushing & respiration practice Practice Discussion 30 10 3rd week Baby massage ․Neonatal management ․Baby massage method Lecture: PPT, baby model 50 Rest ․Offer of beverage 10 Experience-focused education ․Feedback ․Baby massage practice ․Tour to the delivery room & newborn unit ․Delivery experience practice Practice Discussion Field trip 30 10 4th week Breast feeding ․Comprehension of breast feeding method ․Comprehension of breast care & fundus massage ․Comprehension of correct breast feeding posture Posttest, Lecture: PPT, baby model, breast model 50 Rest ․Offer of beverage 10 Experience-focused education ․Feedback ․Correct breast feeding posture practice ․Process of labor respiration & relaxation practice Practice Discussion 30 10

**Parsa 2015 (Iran)** It was a week that people were divided into two groups of 27 people for group counseling. The counseling sessions were conducted by a researcher at the place of physiological childbirth classes.

**Persico 2017 (Italy)** At the first session of antenatal classes, women in the singing cohort received the text of nine lullabies. The recommended lullabies were given by the musicologist who included traditional, loving, and playful songs. Women learned and practiced all nine lullabies without accompaniment, together with the midwife, before each antenatal session. After four weeks, women were invited to choose one or two lullabies as a “leitmotiv” for their own babies and to continue singing at home, in their own time, paying attention to their emotions and their babies’ behavior. The topic of singing lullabies was not covered in standard antenatal classes attended by women in the concurrent cohort. Women in concurrent cohort were, however, left free to singing whatever type of lullabies they wanted.

**Rincy 2014 (India)** Formal instruction was given to the mothers in the study group to monitor fetal movement and maintain kick chart by primi mothers themselves, twice daily for 14 days i.e., 28 times for 14 days along with routine antenatal care. A kick chart performance sheet and a ribbon to knot, when the mother perceived fetal movements during the counting time were given to each mother in the study group.

**Saastad 2011 (Norway)** Women in the intervention group received an information brochure, including instructions on how to use and interpret fetal movement charts, and were asked to count fetal movements daily from gestational week 28. recording the time required to perceive 10 movements. To ensure correct interpretation of the instructions on the counting method, a midwife or an obstetrician from the participating hospitals or the research study group contacted women in the intervention group by telephone 1 to 2 weeks after commencement.

**Salehi 2017 (Iran)** Face to face education about counting and recording the daily fetal movement was provided by the researcher in the intervention group. They were asked to, for four weeks, lie down for half an hour after their breakfast every day on their left side to count and record the movements of the fetus. The subjects in the intervention group counted and recorded fetal movements from the 24th to the 28th week of the pregnancy in specific forms.

**Sanli 2022 (Turkey)** Musical pieces composed in Uşşak mode, which is one of the Traditional Turkish Music (Sufi Music) and facilitates the transition to sleep were played for the participants in the music group. The sound decibels

of mp3 devices to be given to pregnant women were adjusted to the volume in the range of 60–70 dB with the decibel measurement tools. For the music intervention to be effective, women were asked to listen to music for 30 min every other day (Mondays, Wednesdays, and Fridays) for two weeks before going to sleep.

**Sansone 2024 (Australia)** The Prenatal Mindfulness Relationship-Based (PMRB) program is a 9-session mindfulness-based program focused on mother-infant embodied relationship and communication during pregnancy developed specifically for pregnant women. The sessions took place online, run for two hours and occurred weekly for eight weeks during pregnancy. Modifications were made to some components, such as the mindful movement component, and other elements were introduced, such as prenatal psychology education and awareness of the unborn infant’s relational and communication abilities, to ensure they were appropriate for pregnant women. Session 1: Introduction to Prenatal Psychology and discovering the present moment. Session 2: Everything is mindfulness.

Session 3: Discovering embodiment, stress and how it affects us. Session 4: Learning acceptance and emotional availability. Session 5: Self-compassion, self-love and intentionality. Session 6: Cultivating nurturing emotions and conscious communication in the womb. Session 7: Letting go. Session 8: A mindful pregnancy, birth and life

Session 9: Postpartum reunion and birth story.

**Senapati 2023 (India)** One to One basis daily fetal movement count maintenance method for 10 days was explained to all the primi and multi gravida women i.e., twice daily after breakfast and lunch. A dairy and pen were provided to the women and asked to enter the fetal movement count in the diary when they perceive the fetal movements. The compliance toward the fetal movement counting was assured by telephonic calls and WhatsApp messages every

alternate days starting from the 1st day to 10th day.

**Shams 2021 (Iran)** The training sessions were held for the study participants in the intervention group for 6 weeks on Sundays and Tuesdays (n=12 sessions). Each session was held before noon and lasted about 45 to 50 minutes. The music tracks played in the intervention sessions were selected from an album. In this research, the music was

used as a therapy and was delivered in a group. A single 30-minute session of listening to music during TVUS examination. For the music of this research, the authors decided on “Prenatal music album with the sound of nature”

with highest sales volume in the antenatal music section after consulting music charts of online company

M, offline company K, and Y. The music was played using an MP3 player and started when women entered TVUS examination room and kept playing until they left. The volume of the music was adjusted to the

women’s satisfaction. Playing time was limited to single 30-minute sessions.

**Shen 2021 (Taiwan)** The intervention group received a DVD with a video featuring a low impact, 20-min aerobic exercise program developed by the researchers in collaboration with a qualified prenatal yoga teacher. The program

incorporated modified yoga movements suitable for pregnant women. The video opens with an illustration with the written caption: “Please drink adequate water before exercise, wear loose and comfortable clothes, and exercise in a suitable environment. Stop exercising immediately if you feel tired.” A narrator then says, “Now you are ready to

prepare a comfortable, solid chair with a chair back.” With soft music playing in the background, the exercise program proceeds in the following order: warm-up → neck → shoulder → arm → chest → waist → leg → regulated breathing. The initial 14.5 min of the exercise program is performed in a seated position, followed by 3.5 min performed in a standing position, and a final 2 min in a seated position. The series of exercises was arranged to achieve strength-conditioning, moderate-intensity exercise goals. During both the 2-min warm-up and 2-min final

regulated breathing stages of the exercise program, the yoga teacher in the video instructed the viewer to touch the center of her chest with her right hand and her abdomen with her left hand to feel the connection with her fetus. The participants were instructed to use the DVD three times per week for 12 weeks at home.

**Shreffler 2019 (USA)** three intervention groups that received either (1) fetal Doppler heartrate monitors (n=8); (2) four texts per week with mindfulness tasks designed to enhance feelings of maternal attachment (n=11); or (3) both Doppler monitors and mindfulness exercises (n=6). The participants who received fetal Doppler monitors were trained on appropriate use and instructed to listen to their baby’s heartbeat for at least 1 min per day over the two-week period. We examined compliance with the protocol by asking participants during the follow-up how many times per day that they used the Doppler during the study period. The mindfulness exercises were sent via text messages with instructions for the specific exercise assigned that day. All participants in the mindfulness groups received the same text message. Text messages were sent at the same time of day for all groups (mid-evening). Women were asked to “take a few moments alone and complete the following task”, which were different and included: deep breathing, meditation, prenatal massage, responding to kicks, nursery rhymes, telling the baby about a cherished person in their life, planning an activity with the baby, and reading a story to the baby.

**Sedgmen 2006 (Australia)** The ultrasound examinations were carried out in a friendly and relaxed atmosphere by experienced sonographers and radiographers, who provided verbal feedback about the image presented on a separate screen. The 11–14-week screen took approximately 20 min, and the 18–20-week screen took 40 min, depending on the position of the baby at the time of the ultrasound examination. 2D real-time ultrasound provided the usual

images, with movement visible if the fetus was active at the time of examination, while 3D ultrasound provided a surface-rendered image, which produced high-quality 3D ‘lifelike’ images of the fetus, especially of the facial

features18. While 3D ultrasound produces 3D images in which there is no associated movement, those women receiving 3D also received a 2D real-time ultrasound examination. Figures 1 and 2 compare the 2D and 3D images at 12- and 18-weeks’ gestation.

**Skelton 2023 (UK)** Parent expectations and experiences of pregnancy scans during the COVID-19 pandemic. Topics explored through these questions included searching for information about the scan, what might or did happen during the scan, and thoughts about the scan. A mixture of question types was used, with open-ended questions included to compliment the closed questions so that participants could further elaborate on their answers if

they wanted to. Objective parental experience was quantitatively evaluated through closed questions (e.g., “did you see images of the baby?”). Subjective parental experience was captured in the free-text responses which generated qualitative data. Participants were also asked to report their feelings of anxiety, excitement and satisfaction regarding their scan using a rating scale.

**Skelton 2024 (UK**) Exploring bonding and scan experiences in pregnancy. Ultrasound-parents were identified by clinical staff following completion of their routine first trimester screening scan and MRI-parents were identified by perinatal imaging researchers when booking their research MRI scan.

**Sugishita 2019 (Japan)** Mothers in the intervention group started an intervention program that included listening to “fetal music” and writing a “fetal diary”. ‘Fetal music’ was a relaxing music composed of string instrument (music

boxes) that lasted for about 30 minutes of gentle box music. In the fetal diary, there are provisions for space where mother could write about her fetal movements and her feelings. In the intervention group, participants listened to fetal music 2-3 times daily and wrote in a fetal diary every day.

**Thomas 2014 (Australia)** The antenatal group program comprised six 2-h sessions held on a fortnightly basis, including two sessions with participants and their partners. The program had four core components: (1) several behavioral self-care strategies; (2) a psychoeducational component focusing on mood monitoring, early detection and contingency planning for emerging anxiety and depression in pregnancy and the postpartum (including information regarding community-based supports); (3) an interpersonal therapy (IPT) component addressing social support, couple communication, role transitions and awareness between the couple of each other’s mental health warning signs; and (4) a parent-infant relationship component addressing infant attachment needs, positive parental responsiveness and bonding with infants (including discussing the role of fathers). Partners attended two sessions (the fourth and sixth sessions). The first partner session covered (1) adjustment to parenthood and changes across all life domains, (2) psychoeducation of maternal and paternal mental health, (3) mood monitoring and detection of early and late warning signs of depression and anxiety, and (4) coping plans to manage emerging and later signs of depression and anxiety and crisis plans. The second partner session addressed two main themes: (1) acknowledging changes in the couple relationship during early parenthood and how to support and communicate with each other during this adjustment phase and (2) parent-infant component focusing on the involvement of fathers right from the start. These sessions involved information sharing, group brainstorming activities and couple communication exercises. The content and format of the group program was developed using established therapy components (CBT and IPT, parent-infant interventions) and refined with consumer participation from past patients of the service who had received psychological intervention for postnatal depression. The content of the draft program was reviewed by consumers who were also asked to reflect upon what information and resources they would have found helpful having in their pregnancy. The initial group program comprised five sessions (including one partner session), which was later extended to six sessions (including two partner sessions) based on feedback from participants and their partners.

**Toosi 2014 (Iran)** The subjects in the experimental group underwent relaxation training at specified times, in addition to receiving the routine prenatal care. The first session concerned with pregnancy changes in relation to anatomy, physiology, and hormonal aspects alongside their psychological impact on physical and mental status of the pregnant women. The emphasis should be placed on the strategies for compliance with pregnancy changes. These include proper nutrition, personal hygiene, physical and mental health and recognizing the ways of improving compatibility with pregnancy changes such as relaxation and their impact on pregnancy. The second session allocated to educating pregnant women on fetal development in different months of pregnancy, the impact of nutrition and health care for mother and fetus, the effect of relaxation on reducing anxiety in pregnancy and its impact on physical and mental health of the mother and fetus. The third session of the training program was devoted

to information on ways of treating threatened signs in pregnancy, the circumstances of forming maternal-fetal attachment, the impact of relaxation on quality of sleep and nutrition of anxious pregnant women, and the effect of relaxation on maternal-fetal attachment. The fourth session of trainings comprised the impact of relaxation on delivery process and postpartum recovery, lactation, and postpartum depression and familiarization with the process and stages of birth, postpartum care, and breastfeeding. At the end of all sessions, the Benson Relaxation method was rehearsed. To ensure that participants practice the relaxation techniques, in addition to relaxation training, they were provided with compact disks (CDs) and were asked to record the daily trainings in each checklist. At the beginning of the next meeting after gathering the checklist, educational materials were reviewed and questions were answered. Practical training, role play, lectures and educational CD were used to teach relaxation. The most important practical training given by Benson method included settling in a quiet environment, mental preparation, passive attitude, and response in a comfortable position, the deep loosening of muscles from foot to the top for 10 to 20 minutes and finally reversing slowly.

**Toosi 2017 (Iran)** Mothers in the intervention group were divided into two 20-subject groups and participated in four 90-min educational classes weekly held on Saturdays. It should be mentioned that the intervention group received relaxation training in addition to the routine pregnancy care. At the end of all the educational sessions, Benson’s relaxation technique was performed. This method is very easy to learn and does not take more than 10e-0 min to perform (from the tip of toes to forehead). Benson’s relaxation technique can be done while lying down or sitting on the chair. To make sure that women did the relaxation technique at home, in addition to an educational CD, they were provided with a checklist to record their daily exercises. The mothers were required to perform the relaxation technique at least once a day. At the beginning of each educational session, the checklists were collected, the educational content of the previous session was reviewed, and the participants’ questions were answered. The most important practical trainings included staying in a calm environment, mental preparation, passive attitude, staying in a comfortable status, relaxation of all body muscles from the sole of the foot towards the upper extremities (for 10-20 min), and slowly getting out of relaxation. First session Anatomical, physiological, and hormonal changes during pregnancy, sperm and ovum, stages of IVF, physiology of fetal development, signs of anxiety, effects of anxiety on pregnancy, strategies for more adaptation with changes in pregnancy including appropriate nutrition, individual health, physical health, mental health, identification of methods for improving compatibility with changes in pregnancy such as relaxation, and effects of relaxation. Second session Development of fetus in different months of pregnancy, nutrition during pregnancy, effect of healthy nutrition and pregnancy care on maternal and fetal health, effect of relaxation on reduction of anxiety, and effect of relaxation on mother and fetus’ physical and mental health. Third session Signs of risk in pregnancy and ways to cope with them, and effect of relaxation on improvement of mother’s sleeping and nutrition, maternal-fetal attachment, and fetal growth. Fourth session Effect of relaxation on delivery process, necessity to perform relaxation during delivery, effect of relaxation on improvement after delivery, breastfeeding, and reduction of postpartum depression.

**Wahyuni 2024 (Indonesia)** The overall intervention implementation activities were carried out for 12 weeks. The implementation of assistance to pregnant women was carried out for a minimum of eight weeks. Assistance is carried out using various methods, such as gathering pregnant women in a room to be given treatment in pregnant women’s classes, discussions via WhatsApp groups, and home visits. Week 1: Pretest, then the activity continues with providing education to pregnant women and husbands about prenatal stimulation. Week 2: Assistance to pregnant women by nurses by providing further education about 1 Times 100 Minutes 1. Spiritual aspects that underlie prenatal parenting towards readiness to become mothers; 2. Spiritually based prenatal stimulation and practice and role play by each pregnant mother and her partner about prenatal stimulation. a. Auditory stimulation involved engaging in conversations with the fetus, introducing the concept of God, familiarizing the fetus with both mother and father, and participating in joint prayers for 20 minutes. b. Kinesthetic stimulation was performed using by tapping on the baby’s buttocks for 5 minutes, c. Temperature stimulation alternated between warm and cold baths for 10 minutes. d. Light-dark stimulations was administered using a flashlight for 10 minutes. Week 3-11: Independent interventions by the mother who continue to receive assistance and monitoring 8 Times 60-90 Minutes. Week 3-4: Assistance to pregnant women by nurses, with peer group couples, strengthening husbands’ support in prenatal stimulation carried out at agreed times by conducting home visits. 5 Times 60-90 Minutes. Week 5-7: Assistance to pregnant women by nurses by sharing experiences of applying spiritual-based prenatal stimulation to pregnant women in groups carried out online via the Whatsapp group. Every day 60-90 Minutes (21 days). Week 8-11: Assistance to pregnant women by nurses, with overall monitoring and evaluation of the implementation of spiritual-based prenatal care for pregnant women in groups carried out at agreed times through home visits 4 Times 60-90 Minutes.

Week 12: Posttest was performed when pregnant women were taken to the antenatal care class. 1 Times 30 Minutes.

**Weis 2012 (USA)** Eight semi structured classes lasting 11/2 hours each provided the opportunity for gravid women to process their pregnancy experiences, foster reflectiveness, promote self-understanding, facilitate coping, and enhance their capacity for social support for each other. Each session mirrored a chapter in the participant manual titled Birth of a Mother. The chapters’ content aligned with the dimensions of maternal psychosocial adaptation) as

well as the sessions of the intervention. The manual is designed to provide reflection for the participants with fill-in-the-blank questions throughout. The first chapter titled “Accepting Pregnancy and Visualizing Motherhood” focuses on a woman’s response to her pregnancy rather than the infant and her tolerance of discomforts and adjusting to body changes. It addresses concerns over role changes resulting from pregnancy. The second chapter “Internalizing Motherhood” aligns with the maternal psychosocial adaptation dimension of identification to the motherhood role. During the second session, the facilitated discussion focuses on one’s motivation for motherhood. Women were

provided an opportunity to appraise their life situation, their fantasies, dreams, and hopes for their childbirth experience and that of being a mother. This session includes discussion regarding the maternal grandmother’s support and expectations she may be placing on her daughter. The third chapter “Family Relations” aligns with the maternal psychosocial dimensions of relationship with husband and relationship with mother. These discussions

probe into family, particularly the husband or partner’s role and his support during the pregnancy. For women experiencing deployment of their spouses, discussion time is needed to explore contact and support provided from afar. The mentor/facilitator guides conservation about the transition the husband is going through during the pregnancy. The fourth session does not align with any chapter but takes concepts throughout the manual and focuses on military family adjustments, coping in the military, building one’s self-esteem and identity as a military wife and mother. During this session, the women can voice their concerns about being military wives and mothers. The mentor/facilitators answer questions the women may have regarding life in the military. This session in addition to the third session incorporates aspects of post deployment reintegration for the father/husband into the family. This lesson is particularly important due to the addition of a new family member and the wife who is possibly a mother for the first time. The fifth session aligns with the fourth chapter “Childbirth Concerns.” This chapter delves into unvoiced fears the woman may have regarding childbirth or the health of her unborn infant. Women discuss specific fears they have regarding childbirth and the safety of their unborn child. In addition to the mentors, a nurse midwife or a perinatal nurse practitioner attends the session to assist with questions and validate or

dispel concerns. Plans for labor, especially labor support during spousal deployment, are explored. This session aligns with three of the seven dimensions of maternal psychosocial adaptation, “well-being of self and baby, preparation for labor, and fear of helplessness and loss of control.” The sixth week aligns with discussion of care of the infant and postpartum concerns. This session does not specifically align with a dimension from the prenatal measures of maternal psychosocial adaptation, but elements related to the woman’s preparation for motherhood following delivery are evident within the dimensions of “acceptance of pregnancy and identification to the motherhood role.” The seventh session is focused on concerns of the multigravidas. For the multigravidas, labor and delivery are familiar events, however, they rely heavily on prior childbirth memories in formulating their expectations and the supposed challenges for the ensuing childbirth experience. For the women already in a motherhood role, the addition of another child requires a reflection and reformulation of their identity as a mother. The eighth session is designed as a farewell and a party for the women. The women are asked about their home preparations for the infant and whether they need help, particularly for the women with deployed husbands. If the women need help setting up a crib, painting, and so on, arrangements are made for somebody to help. The women are given instructions on obtaining the birth certificate and establishing their newborn information within military and medical benefits systems.

**Westerneng 2021 (Netherlands)** In the questionnaire at T2, women were asked whether they had received any ultrasounds since the completion of questionnaire T1, and if so, how many of these were initiated by their midwife (either routine or clinically indicated) and how many by means of their own request (keepsake). Based on this information and the timing of the ultrasounds registered in the ultrasonography data, the number of ultrasounds

between T1 and T2 was extracted. Women in the intervention group who received at least one midwife-initiated

ultrasound between T1 and T2, and who received an ultrasound within the period prespecified for the first routine ultrasound, were marked as having received a third-trimester routine ultrasound.

**Williams 2015 (USA)** Weekly check-ins with the participants concentrated on discussing the women’s practice of the mindfulness techniques in their everyday life outside of class. On the final day of class and four months after the completion of the program, an in-depth focus group provided the women with a chance to offer feedback regarding the effectiveness of the program including the instructor, structure of the class, class ambience and time allocated to mindfulness practice. the purpose of this study was to measure the impact of a prenatal, mindfulness-based yoga program.

**Yang 2010 (Korea)** The experimental couples were provided with a taegyo program for 4 weeks.

**Yuan 2018 (China)** We developed a 3-week paternal-fetal attachment pilot intervention, 1 session each week, with respect to another paternal-fetal attachment intervention, which has been shown to have positive effects in the occupational context. The intervention was carried out using the method of group discussion, lecture, question and answer, screening movie and educational booklet, and homework. The contents of the training intervention program include getting to know the fetal growth stages, common problems and physical and psychological changes in pregnant women, spouse's duties and father's role, the concept of attachment and paternal-fetal attachment and ways of communication with the fetus and attachment behaviors. All paternal-fetal attachment techniques taught in our intervention concentrated on 3-session range planning, focusing on the following weeks.

**Zhang 2020 (China)** There is a wide variety of prenatal education available for pregnant women. we aimed to identify the prevalence of depression in the trimester, the influence of prenatal education on women’s prenatal depression score and MFA score.
