# Supplementary material for: Prenatal attachment interventions: a comprehensive systematic review and meta-analysis
Source: Arch Womens Ment Health. 2025 Nov 8;28(6):1447–71. doi: 10.1007/s00737-025-01630-w (PMC12702810; doi:10.1007/s00737-025-01630-w)

**Supplementary Figure 4A** Risk of bias summary plots

**Risk of Bias in Randomized Studies of Interventions**

**
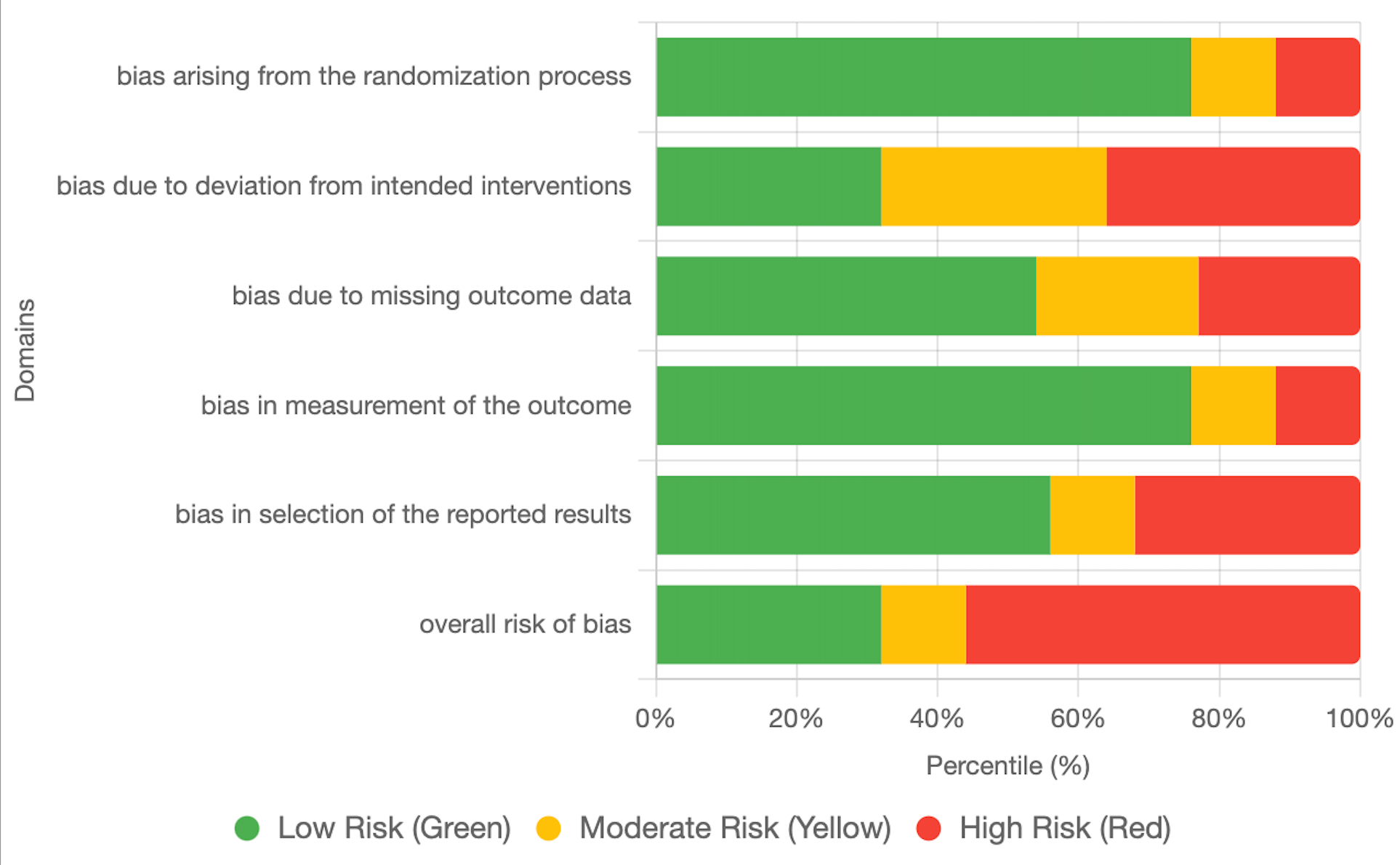
**

**Risk of Bias in Non-randomized Studies of Interventions**


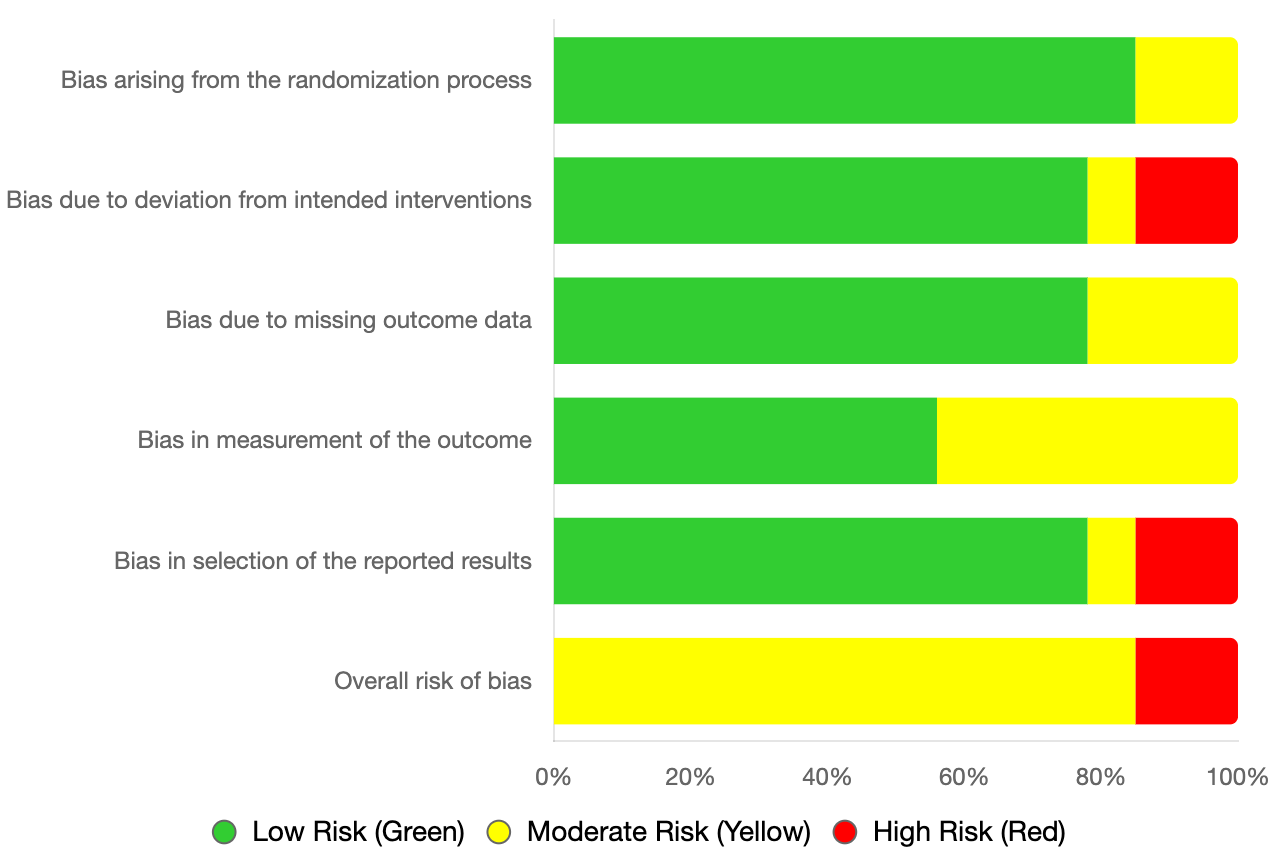

Supplement: Supplementary file 4 — Supplementary file4 (DOCX 381 KB) [file 737_2025_1630_MOESM4_ESM.docx]
