# Supplementary figures and images for: Prenatal attachment interventions: a comprehensive systematic review and meta-analysis
Source: Arch Womens Ment Health. 2025 Nov 8;28(6):1447–71. doi: 10.1007/s00737-025-01630-w (PMC12702810; doi:10.1007/s00737-025-01630-w)

**Supplemental Figure 4B.** Funnel plots showing publication/small study bias


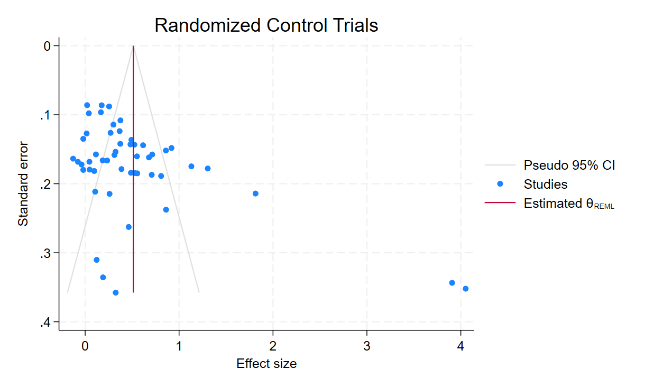

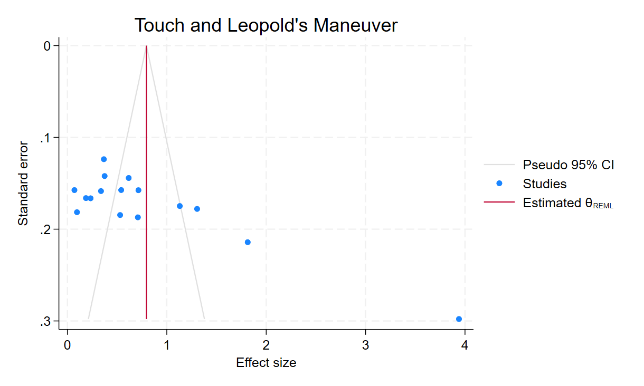


**A B**


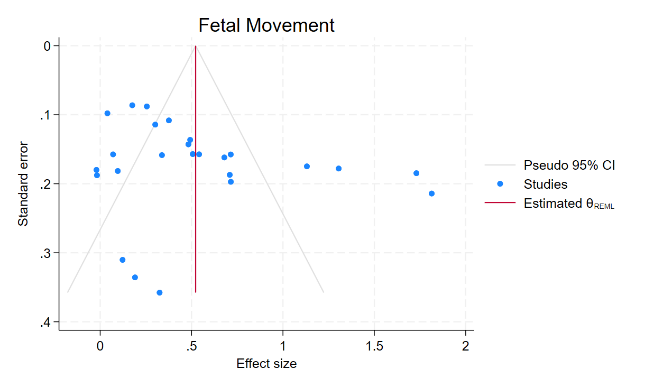

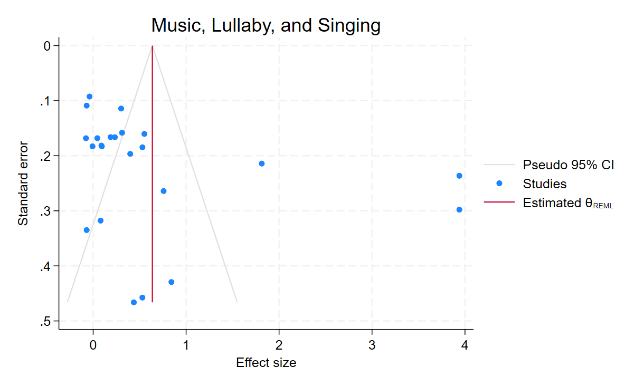


**C D**


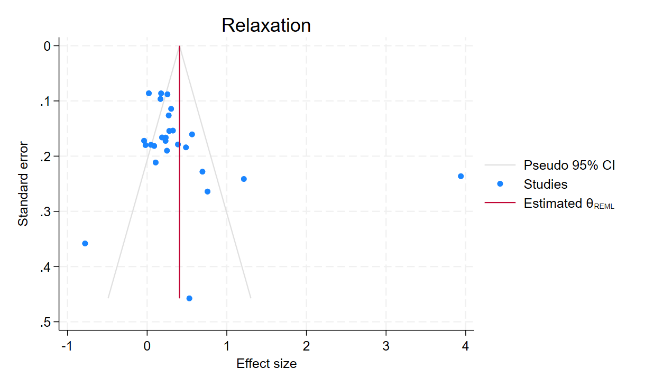

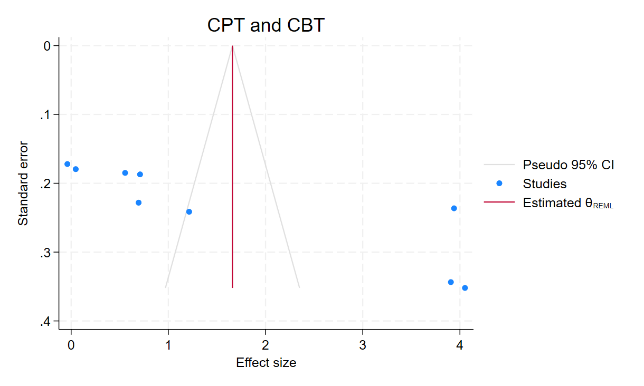


**E F**


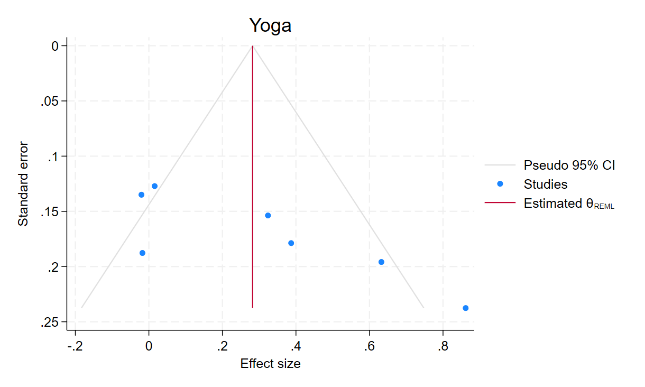

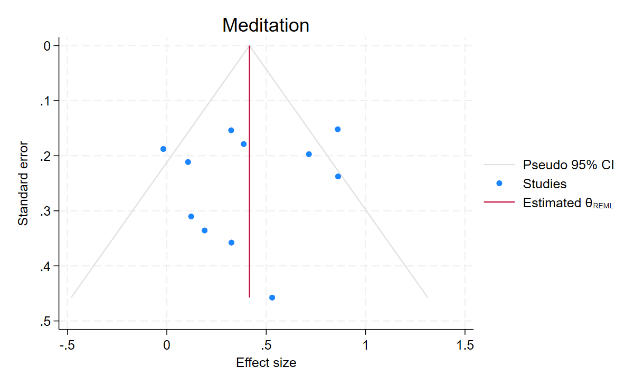


**G H**


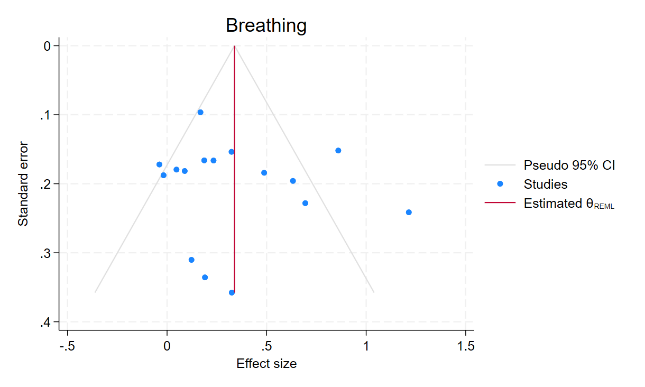

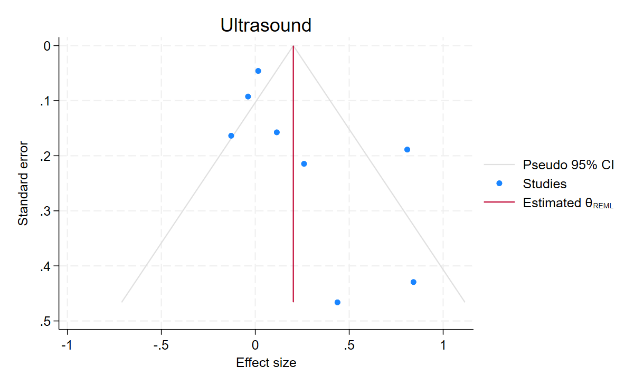


**I J**


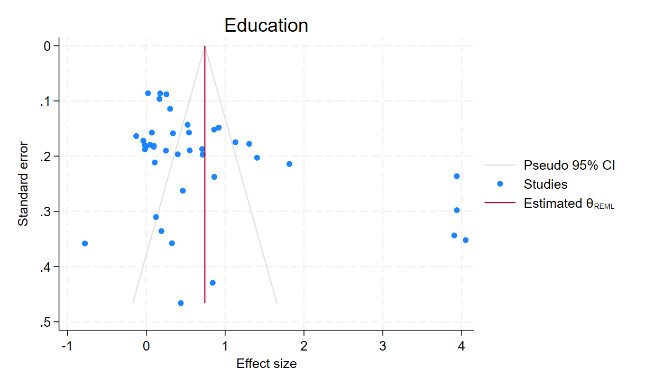

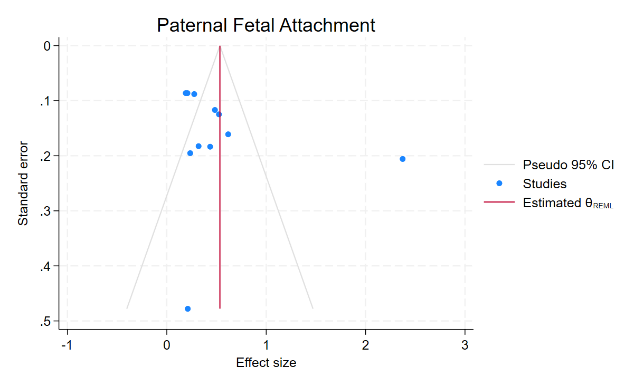


**K L**

Supplement: Supplementary file 5 — Supplementary file5 (DOCX 306 KB) [file 737_2025_1630_MOESM5_ESM.docx]
